# New Mesoporous Silica-Supported Organocatalysts Based on (2*S*)-(1,2,4-Triazol-3-yl)-Proline: Efficient, Reusable, and Heterogeneous Catalysts for the Asymmetric Aldol Reaction

**DOI:** 10.3390/molecules25194532

**Published:** 2020-10-03

**Authors:** Omar Sánchez-Antonio, Kevin A. Romero-Sedglach, Erika C. Vázquez-Orta, Eusebio Juaristi

**Affiliations:** 1 Departamento de Química, Centro de Investigación y de Estudios Avanzados, Avenida IPN # 2508, 07360 Ciudad de México, Mexico; sancheza@cinvestav.mx (O.S.-A.); sedglach13@gmail.com (K.A.R.-S.); caritovazquez23@gmail.com (E.C.V.-O.); 2 El Colegio Nacional, Luis González Obregón # 23, Centro Histórico, 06020 Ciudad de México, Mexico

**Keywords:** (S)-proline, supported organocatalysts, asymmetric aldol reaction, reusable organocatalysts

## Abstract

Novel organocatalytic systems based on the recently developed (*S*)-proline derivative (2*S*)-[5-(benzylthio)-4-phenyl-(1,2,4-triazol)-3-yl]-pyrrolidine supported on mesoporous silica were prepared and their efficiency was assessed in the asymmetric aldol reaction. These materials were fully characterized by FT-IR, MS, XRD, and SEM microscopy, gathering relevant information regarding composition, morphology, and organocatalyst distribution in the doped silica. Careful optimization of the reaction conditions required for their application as catalysts in asymmetric aldol reactions between ketones and aldehydes afforded the anticipated aldol products with excellent yields and moderate diastereo- and enantioselectivities. The recommended experimental protocol is simple, fast, and efficient providing the enantioenriched aldol product, usually without the need of a special work-up or purification protocol. This approach constitutes a remarkable improvement in the field of heterogeneous (*S*)-proline-based organocatalysis; in particular, the solid-phase silica-bonded catalytic systems described herein allow for a substantial reduction in solvent usage. Furthermore, the supported system described here can be recovered, reactivated, and reused several times with limited loss in catalytic efficiency relative to freshly synthesized organocatalysts.

## 1. Introduction

In the last two decades organocatalysis has developed into an important strategy in asymmetric organic synthesis because of its enormous potential in the efficient preparation of chiral molecules [1,2,3,4,5]. In this regard, immobilization and recycling of asymmetric organic catalysts is of significant current interest [6]. Of particular relevance is research involving immobilization and recycling of organic catalysts such as proline and proline derivatives [7,8]. In order to provide useful catalytic materials to be applied in stereoselective transformations, one important challenge with catalyst immobilization is to retain the activity and stereoselectivity of the immobilized catalysts. Furthermore, the separation and recovery of the immobilized catalysts should be readily achieved by a simple operation such as filtration.

In this context, polymeric materials are widely used as useful supports in heterogeneous organic synthesis and catalysis [9]. For example, several small peptides have been immobilized on insoluble supports such as silica in order to get easily recyclable catalytic materials [10]. Thus, immobilization of organocatalysts on insoluble supports allows recovery and recycling of organic catalysts, thus providing more sustainable synthetic protocols [11].

In this context, several representative organocatalysts present two or more functional groups that act in cooperative or bifunctional strategies [12,13,14,15,16,17,18]. Several research groups have used natural and unnatural amino acids and peptides [19,20,21,22,23,24,25,26,27,28,29,30,31], chiral ureas and thioureas [32,33,34,35,36,37,38,39,40], and chiral amides [41,42,43,44,45] as building blocks or templates in organocatalyst design. In this context, a significant number of (*S*)-proline derivatives have been developed while searching for an improvement in the catalytic efficiency and stereoselectivity [46,47,48,49,50,51,52,53].

The present work was inspired by recent studies describing novel mesoporous and nanoporous materials based on modified silicas, PEG-resins, zeolites, and MOFs that function as solid supports for the corresponding organocatalytic modules and are capable to catalyze organic reactions such as the Henry reaction, Negishi coupling, and the aldol reaction [54,55,56,57,58,59,60,61,62,63,64,65,66,67,68]. Remarkably, mesoporous and nanoporous silicas [58,62,67,69,70,71] have been widely used as hosts for organocatalytic acids or bases, which by comparison with other solid supported-materials are more affordable, versatile and more resilient in a variety of synthetic organic transformations [72,73].

On the other hand, our research group recently reported the preparation of three novel and efficient (*S*)-proline-derived organocatalysts containing a 1,2,4-triazolyl-5-thione moiety, that proved highly efficient in asymmetric aldol reactions [74]. Relevantly, this organocatalytic system does not require of the presence of any additive to accomplish the desired transformation affording an eco-friendly protocol that avoids extra purification procedures to obtain the corresponding aldol products. Furthermore, one can take advantage of the 1,2,4-triazole moiety as a hydrogen bridge inducer to potentially anchor electrophilic aldehydes used in the aldol reaction (see ref. [74] for more details about the reaction’s mechanism and the synthesis of the catalyst).

Herein we report the development of four novel supported catalysts that incorporate such (*S*)-proline-based organocatalysts on mesoporous commercial silica. Significantly, the resulting heterogeneous organocatalysts proved highly efficient providing the desired aldol products without the need of laborious purification procedures. Furthermore, the silica-supported organocatalysts can be conveniently recycled and reused in a green aldol reaction protocol.

## 2. Results and Discussion

**Scheme 1:**
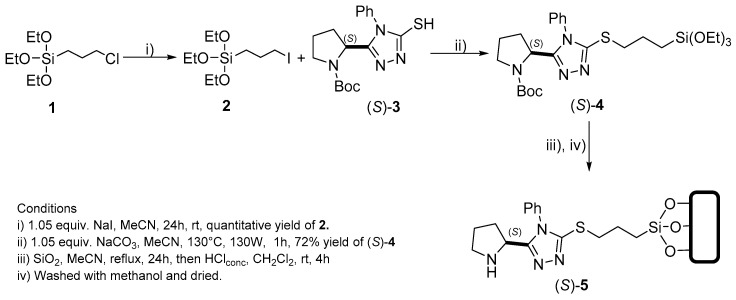
Synthetic route for the preparation of supported organocatalyst (*S*)-**5**.

Nowadays the chemical literature describes two main methodologies employed to covalently bind organic catalysts to silica. The first one is called “co-condensation method” [75,76,77], which consists of the initial synthesis of the required precursors, is followed by a second step involving the coupling or incorporation on the solid support. The second methodology is denominated “post-synthetic grafting”, which focuses on the step-by-step construction of the catalyst on the silica surface [78,79,80,81]. As it turned out, the present development required a hybrid synthetic approach starting from commercial silica and involving both co-condensation and post-synthetic grafting starting from commercial silica, as illustrated in the preparation of compound (*S*)**-5** (Scheme 1).

Thus, seeking to increase the reactivity of the halide as leaving group for SN_2_ reactions, a Finkelstein-type reaction was carried out on (3-chloropropyl)triethoxysilane **1** to obtain the corresponding iodide derivative **2** in quantitative yield [82]. Siloxyiodide **2** was subjected to an SN_2_ substitution reaction with thiol (*S*)-**3** (synthetized according to the previously described procedure [74]) under basic conditions to obtain thioether (*S*)-**4** in 72% yield. Subsequently 1.0 mmol of thioether (*S*)-**4** was exposed to 2.64 g of commercial silica and heated to reflux for 24 h. The silica-supported derivative (*S*)-**5** was filtered and treated with five drops of conc. HCl to remove the *N*-*tert*-butoxy carbonyl (*N*-Boc) protecting group. Lastly, the resulting suspension of the silica-supported catalyst was filtered, and impurities were removed under Soxhlet extraction (see general procedure 7). Finally, the solid product (*S*)-**5** was filtered, dried, and isolated. According to gravimetric analysis, 18% (0.18 mmol, 44.22 mg) of (*S*)-**4** was incorporated to 2.64 g of silica; this amount represents 1.67% *w/w* of the organocatalyst on the support.

**Scheme 2:**
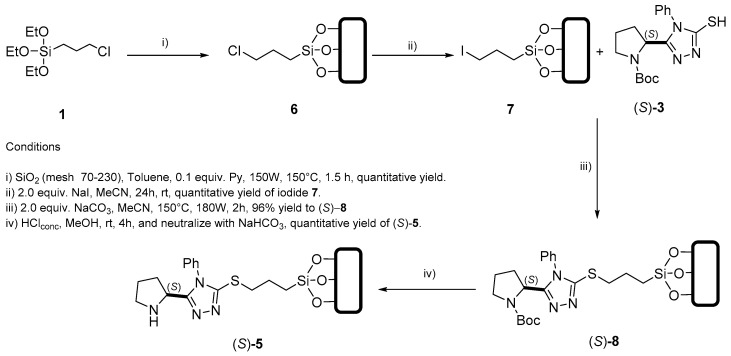
Optimized synthetic route for the preparation of silica-supported organocatalyst (*S*)**-5**.

On the other hand, an alternative, step-by-step strategy for the incorporation of the organocatalyst to the silica´s surface was explored. Scheme 2 shows the corresponding procedure: triethoxide silane **1** was reacted with silica, under microwave activation (MW) for 1.5 h in the presence of 0.1 equiv. of pyridine and toluene as solvent [79]. The crude product **6** was filtered and washed with hexane, EtOAc, acetone, water and MeOH before it was dried under vacuum to afford pure silica-supported chloride **6** in quantitative yield. Siloxy-chloride **6** was subjected to a Finkelstein reaction to exchange chloride for iodide to afford derivative **7** in quantitative yield. Subsequently, siloxy-iodide **7** was suspended in acetonitrile and treated with thiol (*S*)**-3** in the presence of base and under MW irradiation to afford silica-supported, *N*-Boc-protected organocatalyst (*S*)**-8**. Finally, removal of the *N*-Boc group was achieved with conc. HCl to give (*S*)**-5** in 96% yield.

Once the supported organocatalyst (*S*)**-5** was available in sufficient quantity, its load on the silica support was determined by TGA and gravimetric analysis (see Supplementary Materials), finding that heterocycle (*S*)**-3** had been incorporated in a ratio of 114.6 mg (0.4641 mmol) per gram of silica, which represents a 4.34% *w/w* ratio. This load is significantly higher than the one observed according to the synthetic route described in Scheme 1. For this reason, the approach described in Scheme 2 was employed in the preparation of additional silica-supported organocatalysts. In terms of composition, morphology, and organocatalyst’s distribution in different batches of material, the reproducibility of the synthetic protocol was rather good, as evidenced by the appearance of representative micrographs. In the case of particle size, conventional heating results in reduced fragmentation of the samples, relative to preparation under microwave heating; nevertheless, the difference is not significant.

**Scheme 3:**
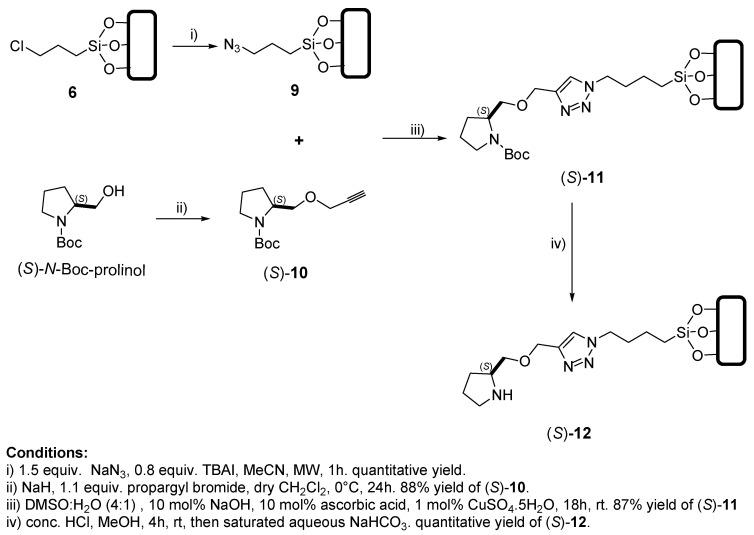
Convergent synthetic route followed for the preparation of (*S*)**-12** using the “in situ generation” approach.

Scheme 3 depicts the synthetic route followed to incorporate a 1,2,3-triazole group as a connecting block within the organocatalytic system. According to previous reports, the triazole segment could act as a good hydrogen bond acceptor at the transition state of the aldol reaction increasing the rigidity of such transition state and in this way possibly increasing the stereoselectivity of the organocatalytic system [83]. In the present system, the triazole fragment was used as spacer and connector between the silica support and the prolinol group that is involved in enamine formation, since it was anticipated that this would enable hydrogen bond interactions to anchor electrophilic substrates during the aldol reaction [83,84,85,86,87].

Firstly, chloride **6** was subjected to an SN_2_ reaction to introduce an azide group in derivative **9**. This substitution reaction proceeded in quantitative yield. On the other hand, *N*-Boc-(*S*)-prolinol, which was prepared according to previous literature reports [88], was used as starting material to generate, by means of NaH treatment followed by propargyl bromide addition, the corresponding propargyl ether derivative (*S*)**-10** in 88% yield [89,90]. Subsequently, compounds **9** and (*S*)**-10** reacted under the conditions of a Huisgen type-reaction [79,84,91], followed by a deprotection process in acidic media. Following neutralization with NaHCO_3_, a silica-anchored organocatalyst (*S*)**-12** was obtained in 87% yield (Scheme 3).

**Scheme 4:**
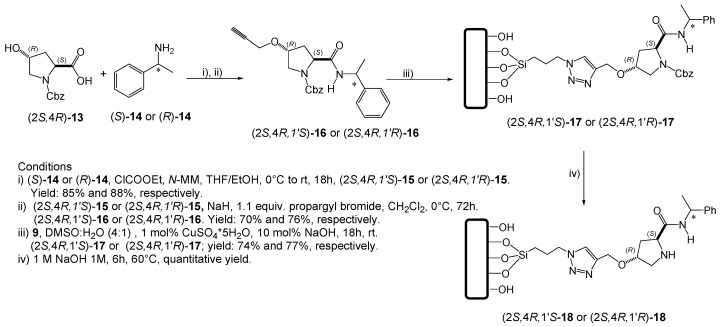
Synthetic route followed to generate (2*S*,4*R*,1′*S*)**-18** and (2*S*,4*R*,1′*R*)**-18**.

Organocatalysts (2*S*,4*R*,1′*S*)**-18** and (2*S*,4*R*,1′*R*)**-18** incorporate an amide functional group in the (*S*)-proline-framework. This structural feature has proved rather successful in other organocatalysts derived from (*S*)-proline [42,43,44]. Furthermore, the fragment of (*R*)- or (*S*)-phenylethylamine was introduced to examine the potential effect of an additional center of chirality in the organocatalytic system. The synthetic route is outlined in Scheme 4. Firstly, *trans*-(2*S*,4*R*)-hydroxyproline was *N*-protected with benzyl chloroformate to provide the substrate (2*S*,4*R*)-**13**, which was activated with ethyl chloroformate before treatment with (*R*)- or (*S*)-phenylethylamine [(*R*)-**14** or (*S*)-**14**]. Then the coupling products were treated with propargyl bromide to afford the propargylic ethers (2*S*,4*R*,1′*S*)**-16** and (2*S*,4*R*,1′*R*)**-16** in 67% overall yield. These terminal alkynes were subjected to a Huisgen-type protocol [79,84,91] with azide **9** and deprotected under basic conditions to afford (2*S*,4*R*,1′*R*)**-18** or (2*S*,4*R*,1′*S*)**-18** in 77% of yield in both cases.

**Figure 1:**
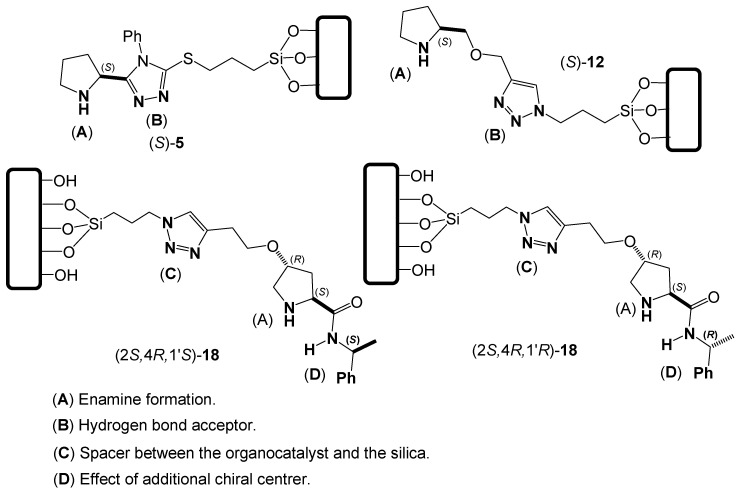
Novel silica-supported organocatalysts synthesized in this work.

Figure 1 presents the four silica-supported organocatalysts developed in this work to carry out asymmetric aldol-type reactions.

### Evaluation of Organocatalysts (S)-5, (S)-12, (2S,4R,1′S)-**18** and (2S,4R,1′R)-**18**

With the four novel silica-supported organocatalysts at hand, their catalytic efficiency in the enantioselective aldol reaction between cyclohexanone and 4-nitrobenzaldehyde was assessed. Table 1 summarizes the outcome of the reactions that were carried out initially. First, and according to previous work [74] it was assumed that best results would be observed under neat conditions or in the presence of water. However, as it can be appreciated in entries 1 and 2, although reaction yields were quite high, the observed stereoselectivities turned out to be low. Therefore, the potential beneficial effect of various additives that are commonly used as adjuvant to increase the strength of non-covalent interactions in the transition state of the catalytic cycle [92,93,94,95] was examined (entries 3–6 in Table 1).

Best results, in terms of yield (99%) and enantioselectivity (92% ee) were recorded with silica-supported organocatalyst (*S*)**-5**, employing an amount of silica equivalent to 10 mol% of catalyst and 10 mol% of *p*-nitrobenzoic acid as additive, under solvent-free conditions (Table 1, entry 6). Nevertheless, the diastereoselectivity was only moderate.

In the case of organocatalyst (*S*)**-12**, although the reaction yield is comparable to those observed with (*S*)**-5**, the catalyst has the capacity to give moderate-to-high diastereoselectivity in the presence of water as additive (see entry 10 in Table 1). Similar stereoselectivity was observed with diastereomeric organocatalysts (2*S*,4*R*,1′*S*)**-18** and (2*S*,4*R*,1′*R*)**-18**. This observation suggests that the α-phenylethyl group is not sufficiently close to the reacting site to provoke noticeable stereoinduction.

Once the best reaction conditions had been established (Table 1, entry 6), substrate screening was performed in aldol-type reactions. Several aromatic aldehydes were tested as electrophiles obtaining excellent yields in all cases and moderate diastereo- and enantio-selectivities (Table 2).

As seen in Table 2, rather high yields are recorded in all cases, although the observed diastereomeric and enantiomeric values are moderate. With these results on hand it was decided to extend the reaction scope using acetone as enamine precursor. It is well known that it is difficult to control the selectivity of enantioselective aldol reaction employing acetone [96,97,98,99,100,101] owing to its high symmetry. Initially, an optimization of the reaction conditions to obtain (*R*)-**27** was carried out finding that best results are obtained under solvent-free reaction conditions, employing (*S*)-**5** as organocatalyst and at a temperature of 1 °C (Table 3).

Table 3 shows the results achieved in the asymmetric aldol reaction employing acetone as enamine precursor and several aromatic aldehydes as electrophiles. In a control experiment, the reaction does not proceed in the absence of organocatalyst. By contrast, excellent reaction yields were recorded when catalyzed by (*S*)-**5**, although the enantioselectivity changed from low to moderate. In entries 4, 6, 9 and 10, a purification process was necessary to isolate the pure aldol products because of minor impurities; nevertheless, in the rest of the cases, no purification was necessary. The product was simply filtered and dried, which represents a great advantage in terms of sustainability of the process, because of the minimal amount of solvent required to purify the products [102,103,104,105].

A silica-supported organocatalyst (*S*)-**5** was also evaluated in the aldol reaction between the enamine derived from cyclohexanone and relevant isatins. Table 4 summarizes the results that exhibit excellent yields and moderate diastereo- and enantio-selectivities. It is worthy of mention that recovery of the catalyst required a reactivation process (see Material and Methods section).

One important advantage when using immobilized catalysts and supported organocatalytic systems is that the catalyst can be recovered and reused. The reuse of organocatalyst (*S*)**-5**, which is covalently bonded to the supporting material-silica in the present study, is no exception. Indeed, recovery and reuse of the organocatalyst was feasible up to 15 catalytic cycles in the aldol reaction between the enamine derived from acetone and 4-nitrobenzaldehyde, as can be appreciated in Table 5. Although the stereoselectivity was slightly diminished with each cycle, the high reaction yield was maintained.

**Figure 2:**
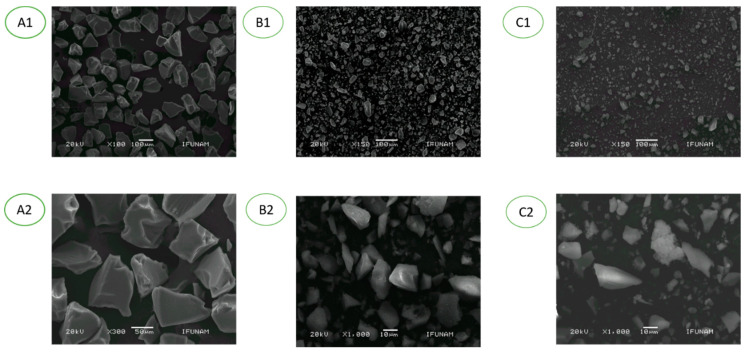

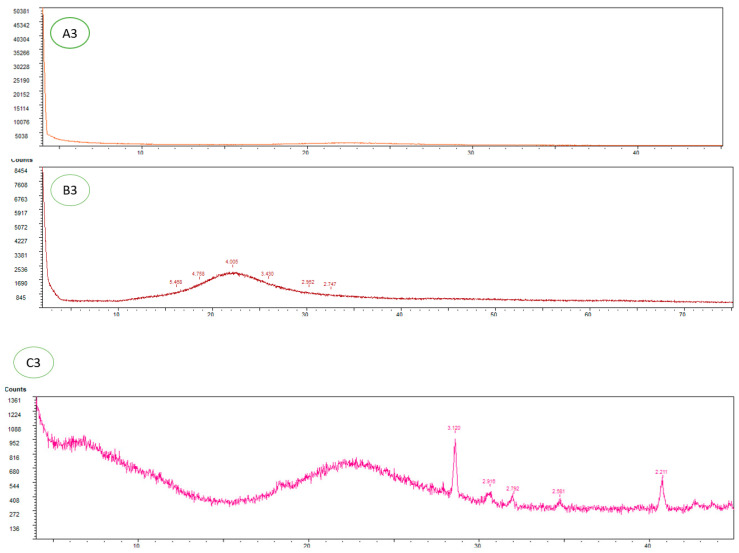
SEM and XRD imaging: **A1**–**A3** commercial silica. **B1**–**B3** Silica-supported organocatalyst (S)-5. **C1**–**C3** Silica-supported organocatalyst (*S*)-**5** after 10 catalytic cycles.

The decrease in catalytic efficiency suggests that the silica support possibly suffers a contamination provoked by an impurity that could be generated during each reaction process. Indeed, comparison of XRD and SEM images of fresh and reused catalyst (*S*)-**5,** shows that both materials present an irregular shape with a smooth surface characteristic of mesoporous amorphous solids (see Figure 2, A1 to C2). Although the particle size corresponds to a mesoporous solid, the micrographic analysis exhibits significant fragmentation of the material apparently provoked by microwave irradiation. Furthermore, comparison of recovered catalyst with commercial silica shows that the former material does not present the characteristic roughness exhibited in commercial silica. In Figure 2, C2 shows the formation of some clusters that can be formed by the accumulation of additive used in each reaction. It is also worthwhile to point out that TGA analysis of a reused catalyst showed that rather than decreasing, the amount of organic material increased (see Supplementary Materials).

As for an XRD analysis, commercial silica Figure 2(A3) exhibits the anticipated diffractogram characteristic of mesoporous material; that is, no peaks are visible. By contrast, in the case of (*S*)-5 Figure 2(B3) a broad signal at ca. 22.2° is recorded, with absence of other peaks confirming that our material conserves its mesoporous structure and suggest that our organocatalyst is well-distributed. Finally, for the recycled (*S*)-5 material Figure 2(C3), one encounters an intense diffraction pattern that apparently is caused by the presence of additive (*p*-nitrobenzoic acid), arising from condensation with active Si-OH groups. Additionally, in order to expand the study of the composition and distribution of the organocatalyst on silica, an FT-IR analysis exhibited some characteristics bands (3500, broad signal from 3300 to 3000 cm^−^^1^ and 2200 to 1900 cm^−^^1^). These signals appear to confirm the presence of the organocatalyst in the material (*S*)-5 (see Supplementary Materials, page S72). Indeed, TGA analysis shows that the amount of organic material present on the silica-supported catalyst (Figure 2(C3)) is greater than that found in freshly prepared (*S*)-5 (Figure 2(B3)). Furthermore, substantial weight loss takes place in the temperature range between 200 and 450 °C, which is in line with leaking of organic material, suggesting that the additive (*p*-nitrobenzoic acid) had incorporated to the silica surface. Indeed, the amount of organic material on the silica-supported material (Figure 2(C3)) is greater than that found in freshly prepared (*S*)-5 (Figure 2(B3)). Therefore, the weight loss that takes place in the temperature range between 200 and 450 °C is in line with the leaking of organic compounds [106,107].

## 3. Materials and Methods

### 3.1. Starting Materials and Their Analysis

Unless otherwise indicated, all reagents were purchased from Sigma-Aldrich and used without further purification. The progress of reactions was routinely monitored by TLC on silica gel 60 (precoated F254 Merck plates) under UV lamp (254 nm) irradiation to visualize starting materials and products. Flash chromatography was performed using silica gel (230−400 mesh).

NMR spectra were acquired on JEOL ECA 500 and Bruker 400 Avance III HD spectrometers. For the acquisition of ^1^H NMR and ^13^C NMR spectra, CDCl_3_ (7.26 and 77.0 ppm, respectively) or DMSO-d_6_ (2.50 and 39.5 ppm, respectively) were used as internal references. Chemical shifts (δ) are reported in parts per million (ppm). Multiplicity was abbreviated as follows: s = singlet, bs = broad singlet, d = doublet, dd = doublet of doublets, t = triplet, q = quartet, quint = quintet, sext = sextet, hept = heptet, m = multiplet. High resolution mass spectra were recorded on an HPLC 1100 coupled to MSD-TOF Agilent series HR-MSTOF mass spectrometer model 1969 A.

The HPLC analysis was performed in a Dionex HPLC Ultimate 3000 with a UV/Visible detector, and with diode array, at 210 and 254 nm, chiral columns such as AD-H for compounds (*R,S*)-38, (*R,S*)-39, (*R,S*)-40, AS-H for compounds (*R*)-27-37 and OD-H for (*R,S*)-26 compound, were employed. IR spectra were acquired on an ATR-IR Varian-640 and are expressed in wave number (cm^−^^1^). Optical rotations were measured on an Anton Paar MCP-100 Polarimeter with reagent grade solvents. Melting points were taken on a Büchi B-540 apparatus and are uncorrected. Microwave-assisted procedures were performed on the Biotage-Initiator microwave system. The micrographic images were taken in a JEOL SEM 5600LV apparatus with an acceleration voltage of 20 kV.

XRD analyses were carried out in a Bruker Advance eco. Goniometer: 280 mm. Axis movement: theta/theta. Detector: one-dimensional “lynx eye”. Generator: 1 kW, Samples are measured with: 25 mA, 30 KV. Radiation: Cu (Kalpha1 + Kalpha2) (1.5416 A). Sample rotation speed: 20 r.p.m. Counting time: 5 s. Step size: 0.02. Degree2-theta measurement interval: 5–55 degrees, and 5–70 degrees. Measurement software: DIFFRAC.MESUREMENT. Analysis Software: DIFFRAC.EVA.

### 3.2. Procedures and General Methods

#### 3.2.1. General Procedure 1: Finkelstein Reaction

In a 50-mL round bottom flask provided with a magnetic bar, the corresponding halide derivative (0.264 g, 1 equiv.), NaI (0.300 g, 2 equiv.) and 10 mL of MeCN or acetone were added. The resulting mixture was stirred for 24 h at room temperature, the solvent was evaporated, and the residue washed with diethyl ether (3 × 10 mL) to give the corresponding iodide product.

#### 3.2.2. General Procedure 2: Propargylation Reaction, in the Synthesis of (*S*)-**10**, (2*S*,4*R*,1′-*S*)-**16** and (2*S*,4*R*,1′-*R*)-**16**

In a 50-mL round bottom flask provided with a magnetic bar, the corresponding alcohol (1 mmol, 1 equiv.), propargyl bromide (1.1 mmol, 1.1 equiv.), NaH (2 mmol, 2 equiv.) and 5 mL of dry CH_2_Cl_2_ were added. The resulting suspension was stirred for 72 h at ambient temperature, the solvent was removed at reduced pressure and the solid residue was washed with diethyl ether (3 × 10 mL). Final purification of the organic phase was achieved on a chromatographic column using hexane-EtOAc eluent [89,90].

#### 3.2.3. General Procedure 3: Huisgen 1,3-dipolar Cycloaddition-Type Reaction (12h), in the Synthesis of (*S*)-**11**, (2*S*,4*R*,1′-*S*)-**17** and (2*S*,4*R*,1′-*R*)-**17**

In the following order, in a 25-mL round bottom flask provided with a magnetic bar, 0.1 equiv. of NaOH, 0.1 equiv. of ascorbic acid and 5 mL of DMSO:H2O (4:1) were added. The resulting mixture was stirred for two minutes before the addition of the corresponding azide derivative (1 equiv.) and CuSO_4_·5 H_2_O (0.1 mol). The reaction mixture was stirred for two minutes and then the propargyl ether derivative was added. The resulting mixture was stirred for 18 h at ambient temperature. The solid that was obtained was filtered and washed with small portions of water, methanol, ethyl acetate, and hexanes. Finally, the residue was dried in a muffle for 24 h at 65 °C [89,90,91].

#### 3.2.4. General Procedure 4: Coupling Reaction, in the Synthesis of (2*S*,4*R*,1′*S*)-**15** or (2*S*,4*R*,1′*R*)-**15**

In a 100-mL round bottom flask, previously purged with argon and provided with a magnetic stirring bar, trans-4-hydroxy-L-*N*-Cbz-proline (1 equiv. 1.0 g, 3.77 mmol) and triethylamine (1.05 equiv., 0.55 mL, 3.96 mmol) were added. Then, 16 mL of dry THF was added and the reaction flask was submerged in an ice bath before the dropwise addition of ethyl chloroformate (1.05 equiv. 0.37 mL, 3.96 mmol). Subsequently, the reaction mixture was allowed to react under vigorous stirring for 15 min at 0 °C. The insoluble Et_3_N·HCl salt that precipitated was filtered off and washed with THF (2 × 6 mL) and the combined filtrate was slowly added to a solution of (*R*)-14 or (*S*)-14 (1.05 equiv. 0.50 mL, 3.96 mmol) in 14 mL of EtOH at 0 °C. The reaction mixture was stirred for 30 min at 0 °C and then at room temperature for 12 additional hours. The solvents were removed under reduced pressure and the resulting colorless oil was dissolved in 30 mL of EtOAc and washed as follows: 5 mL of 1 M HCl, 5 mL of saturated aqueous solution of NaHCO_3_, and brine (2 × 5 mL). The crude product was dried over anhydrous Na_2_SO_4_, concentrated, and purified by flash column chromatography with EtOAc-hexanes (1:1) as eluent to afford pure (2*S*,4*R*,1′*S*)-**15** or (2*S*,4*R*,1′*R*)-**15**.

#### 3.2.5. General Procedure 5: *N*-Boc Catalyst Deprotection Under Acidic Conditions

In a round flask provided with a magnetic stirring bar and MeOH (3 mL), (S)-**4** or (S)-**11** was suspended and the resulting suspension was cooled to 0 °C before the addition of six drops of concentrated HCl (approx. 0.6 mL). The reaction mixture was left standing at room temperature for 4 h, and the product was filtered under vacuum and washed with water. Finally, general procedure 7 was followed until a constant weight of the product was achieved.

#### 3.2.6. General Procedure 6: *N*-Cbz Catalyst Deprotection Under Basic Conditions

An amount of 1 M NaOH (3 mL) and (2*S*,4*R*,1′*S*)-**17** or (2*S*,4*R*,1′*R*)-**17** was added to a round flask provided with a magnetic stirring bar and the resulting mixture was heated to 60 °C for 4 h. The crude product was filtered under vacuum and washed with 1 M HCl and water and finally general procedure 7 was followed.

#### 3.2.7. General Procedure 7: Activation of Catalysts (*S*)-**5**, (*S*)-**12**, (2*S*,4*R*,1′*S*)-**18** and (2*S*,4*R*,1′*R*)-**18**.

Previous to the first use of the corresponding catalyst, it was treated as follows: each silica-supported organocatalyst was placed in a Soxhlet distillation apparatus and washed under continuous refluxing mode with a mixture MeOH:H_2_O:EtOAc (2:1:1) for 14 h. The recovered material was placed on a heating plate for 18 h at 115 °C and finally filtered.

##### General Procedure 7a: Reactivation of Used Silica-Supported Organocatalysts

The recovered silica-supported organocatalyst was washed with methanol and dried in a muffle at 85 °C for 24 h. Once this operation was finished, the catalyst was then ready to be reused.

#### 3.2.8. General Procedure 8: Aldol Reaction

In a round bottom flask provided with a magnetic stirrer, 2.5 equiv. (0.612 mmol) of the corresponding ketone, the corresponding amount of silica-supported organocatalyst (equivalent to 5 or 10 mol%) and the corresponding aldehyde (0.24 mmol, 37 mg, 1 equiv.) were deposited. In case of the use of additive, this was added in the same proportion as the catalyst. The resulting mixture was stirred at the indicated temperature (see Table 1, Table 2, Table 3 and Table 4) until complete reaction. The solid catalyst was recovered by filtration and the filtrate was evaporated at reduced pressure. The purity of samples was evaluated using TLC, and the assessment of the aldol product was established by chromatography.

#### 3.2.9. Procedure for Functionalization of Siloxy-OH Groups on Silica; Obtention of 6

In a 20-mL Biotage^+^ initiator microwave system vial, provided with a magnetic stirrer, 2.64 g of commercial silica (previously activated to 250 °C) was placed before the addition of 0.1 equiv. of pyridine, 1.2 mmol (1.0 equiv.) of 3-chloro-triethoxide silane and 10 mL of toluene. The reactor vial was closed and placed in the microwave oven at 150 °C, 150 Watts for 1.5 h, with the cooler mode activated. The resulting white suspension was filtered and washed with EtOAc (3 × 25 mL) to remove the excess of 3-chloro-triethoxide silane, and finally dried. Yield: quantitative. With this procedure it was possible to functionalize 2.64 g of silica with 1.15 mmol of 3-chloro-triethoxide silane.

### 3.3. Procedure for SN_2_ Type Reaction. Preparation of (S)-**8**

In a 20-mL Biotage^+^ initiator microwave vial, provided with a magnetic stirrer, 2.70 g of **7** was placed, before the addition of 1.1 mmol (0.381 g, 1.0 equiv.) of (*S*)-**3**, 2.4 mmol (0.212 g, 2 equiv.) of NaCO_3_ and 10 mL of MeCN. The resulting suspension was stirred for 10 min, the vial was then closed and placed in the microwave oven at 150 °C, 180 Watts for 2.0 h, with the cooler mode activated. The resulting lightly yellow suspension was filtered and washed with EtOAc (3 × 25 mL) and methanol (3 × 25 mL) to remove the excess of **7**. Yield: 96% of the dried product. With this procedure it was possible to functionalize 2.64 g of silica with 1.12 mmol of **7**.

#### H and ^13^C NMR Spectra, Mass Spectra and Physical Properties

*(S)-tert-Butyl-2-(4-phenyl-5-thioxo-4,5-dihydro-1H-1,2,4-triazol-3-yl)pyrrolidine-1-carboxylate*, (*S*)-**3**, was synthetized according to the procedure described in reference 11 starting from (*S*)-proline. White solid, mp 210–212 °C. Yield: 1.21 g (78%) for 3 steps. [α]_D_^25^ = +13.5°, c = 0.33, CHCl3. ^1^H NMR (500.16 MHz, DMSO-d_6_, 120 °C) δ 13.33 (s, 1H, NH), 7.62–7.26 (m, 5H_ar_), 4.45 (d, 1H, *J* = 4 Hz, CH), 3.25 (s, 1H, CH*H*), 3.15 (s, 1H, C*H*H), 2.07–1.70 (m, 4H, 2 × CH_2_, 1.32 (s, 9H, 3 × CH_3_) ppm. ^13^C NMR (125.76 MHz, DMSO-d_6_, 120 °C) δ 169.3, 154.0, 134.4, 129.9, 128.9, 128.7, 79.7, 53.5, 46.8, 31.8, 28.9, 23.3 ppm. IR (neat) ν_max_ 3212, 2969, 2972, 2879, 1769, 1707, 1658, 1521, 1408, 1401, 1272, 1198, 1127, 774 cm^−1^. HRMS (MS-TOF) calcd. for C_17_H_23_N_4_O_2_S (M)^+^ 347.1536, found 347.1533.

*Triethoxy(3-iodopropyl)silane*, **2**. General procedure 1 was followed to obtain silane **2** in quantitative yield. Yellow oil. ^1^H NMR (500 MHz, CDCl_3_) δ 3.79 (q, *J* = 7.0 Hz, 6 H, 3 × CH_2_), 1.75–2.00 (m, 2H, CH_2_), 3.19 (t, *J* = 7.1 Hz, 2H, CH_2_), 1.19 (t, *J* = 7.0 Hz, 9H, 3 × CH_3_), 0.63–0.77 (m, 2H, CH_2_). ^13^C NMR (125 MHz, CDCl_3_) δ 58.3, 27.5, 18.2, 12.2, 10.5 ppm.

*(S)-tert-Butyl-2-((prop-2-yn-1-yloxy)methyl)pyrrolidine-1-carboxylate*, (2*S*,4*R*)-**10**. General procedure 2 was followed to obtain (2*S*,4*R*)-**10** as a white wax, 1.28 g (88% yield). [α]_D_^25^ = +25.9°, c = 0.33, CHCl_3_. ^1^H NMR (400.0 MHz, CDCl_3_) δ 4.16 (s, 2H, CH_2_), 3.94 (s, CH), 3.67(dd, ^1^H, *J*_1_ = 3.45 Hz, *J*_2_ = 9.04 CH), 3.55–3.24 (m, 3H), 2.03–1.61 (m, 5H), 1.48 (s, 9H, 3 × CH_3_) ppm; ^13^C NMR (125.76 MHz, CDCl3) δ 154.6, 79.9, 79.3, 74.3, 70.6, 58.4, 56.3, 46.7, 28.5, 23.8, 22.9 ppm. HRMS (MS-TOF) calcd. for C_13_H_22_NO_3_ (M)+ 240.1594, found 240.1598.

(2*S*,4*R,*1’*S*)*-Benzyl-4-hydroxy-2-(((S)-1-phenylethyl)carbamoyl)pyrrolidine-1-carboxylate*, (2*S*,4*R*,1′*S*)-**15**. General procedure 4 was followed to obtain (2*S*,4*R*,1′*S*)-**15** as a white foam. Yield: 1.18 g (85%). [α]_D_^25^ = +7.2, c = 0.33, CHCl_3_. ^1^H NMR (500.0 MHz, CDCl_3_) δ 7.48–7.01 (m, 10H, CH_ar_), 6.74 (d, 1H, NH), 5.21–5.03 (m, 1H, CH), 5.03–4.86 (dd, 2H, *J*_1_ = *J*_2_ = 4.98, CH_2_), 4.49–4.33 (m, 1H, CH), 4.26 (m, 1H, OH), 4.19–3.94 (m, 1H, CH), 3.68–3.35 (m, 2H, CH_2_), 2.26–1.82 (m, 2H, CH_2_), 1.52–1.20 (m, 3H, CH_3_) ppm. ^13^C NMR (125.76 MHz, CDCl_3_) δ 171.2, 156.0, 143.3, 136.7, 128.7, 128.2, 127.9, 127.2, 126.08, (d) 68.4, (d) 67.5, (d) 59.5, (d) 55.2, 39.7, 38.5, 22.0. HRMS (MS-TOF): calcd. for C_21_H_25_N_2_O_4_ (M)+ 369.1736, found: 369.182041.

(2*S,*4*R,*1’*R)-Benzyl-4-hydroxy-2-(((R)-1-phenylethyl)carbamoyl)pyrrolidine-1-carboxylate*, (2*S*,4*R*,1′*R*)-**15**. General procedure 4 was followed to obtain (2*S*,4*R*, 1′*R*)-**15** as a white foam, 1.22 g, yield: 88%. [α]_D_^25^ = −2.5, c = 0.33, CHCl_3_. ^1^H NMR (500.0 MHz, CDCl_3_) δ 7.39–7.07 (m, 10H, CH_ar_), 6.27–6.10 (s, 1H, NH), 5.23–5.03 (m, 2H, CH_2_), 5.03–4.92 (p, 1H, *J* = 10 Hz, CH), 4.55–4.45 (bs, 1H, OH), 3.81–3.45 (m, 2H, 2 × CH), 2.67–1.64 (m, 4H, 2 × CH_2_), 1.52–1.17 (m, 3H, CH_3_) ppm. ^13^C NMR (125.76 MHz, CDCl3) δ 170.3, 155.7, 143.4, 136.2, 128.8, 128.3, 128.0, 127.2, 126.0, (d) 68.3, (d) 60.5, (d) 54.5, (d) 49.2, 38.7, 38.5, 22.1. HRMS (MS-TOF): calcd. for C_21_H_25_N_2_O_4_ (M)+ 369.1736, found: 369.183627.

*(2S,4R)-2-Benzyl-(((S)-1-phenylethyl)carbamoyl)-4-(prop-2-yn-1-yloxy)pyrrolidine-1-carboxylate*, (2*S*,4*R*,1′*S*)-**16**. General procedure 2 was followed to obtain (2*S*,4*R*,1′*S*)-**16** as a pale-yellow foam, 0.425 g, yield: 70%. [α]_D_^25^ = +11.7, c = 0.33, CHCl_3_. ^1^H NMR (500.0 MHz, CDCl_3_) δ 7.49–7.05 (m, 10H, CH_ar_), 6.51 (s, 1H, NH), 5.17 (d, 1H, *J* = 13 Hz, CH*H*), 5.08 (d, 1H, *J* = 12.6 Hz, C*H*H), 5.01 (p, 1H, *J* = 6.72 Hz, CH), 4.49–4.32 (m, 1H, CH), 4.32–4.17 (m, 1H, CH), 4.16–3.97 (m, 2H, CH_2_), 3.82–3.56 (m, 1H, CH), 3.48 (dd, 1H, *J*_1_ = 12.6, *J*_2_ = 5.36 Hz, CH), 2.55–2.25 (m, 2H, CH_2_), 2.19–2.01 (bs, 1H, CH), 1.80–1.67 (m, 3H, CH_3_) ppm. ^13^C NMR (125.76 MHz, CDCl_3_). δ 170.2, 156.3, 143.5, 136.4, 128.6, 128.2, 128.0, 127.1, 126.0, 79.5, 75.1, 67.5, 59.2, 56.6, 51.6, 49.1, 33.8, 22.5. HRMS (MS-TOF) calcd. for C_24_H_27_N_2_O_4_ (M)+ 407.1892, found: 407.205297

*(2S,4R)-2-Benzyl-(((R)-1-phenylethyl)carbamoyl)-4-(prop-2-yn-1-yloxy)pyrrolidine-1-carboxylate* (2*S*,4*R*,1’*R*)-**16**. General procedure 2 was followed to obtain (2*S*,4*R*,1’*R*)-**16** as a pale-yellow foam. 0.450 g, yield: 76%. [α]_D_^25^ = −6.8, c = 0.33, CHCl_3_. ^1^H NMR (500.0 MHz, CDCl3) δ 7.50–7.05 (m, 10H, CH_ar_), 6.75 (s, 1H, NH), 5.1 (bs, 1H, CH), 5.06–4.91 (m, 2H, CH_2_), 4.44–4.31 (m, 1H, CH), 4.31–4.18 (m, 1H, CH), 4.11–3.96 (m, 2H, CH_2_), 3.85–3.61 (m, 1H, CH), 3.61–3.50 (m, 1H, CH), 2.53–2.38 (m, 1H, CH), 2.35–2.20 (m, 1H, C*H*H) 2.17–2.00 (m, 1H, C*HH*), 1.50–1.13 (m, 3H, CH_3_) ppm. ^13^C NMR (125.76 MHz, CDCl_3_) δ 170.6, 155.8, 143.3, 136.4, 128.7, 128.6, 128.2, 127.9, 127.2, 126.1, 79.6, 75.3, 67.4, 59.4, 56.5, 52.1, 48.9, 34.3, 22.1 ppm. HRMS (MS-TOF) calcd. for C_24_H_27_N_2_O_4_ (M)+ 407.1892, found: 407.196976.

## 4. Conclusions

In this work, four novel silica-supported organocatalysts were synthesized and characterized: (*S*)-**5**, (*S*)-**12**, (2*S*,4*R*,1′*S*)-**18** and (2*S*,4*R*,1′*R*)-**18**. These catalytic systems turned out to be efficient and versatile organocatalysts for the asymmetric aldol reaction, affording the desired aldol products in excellent yields and moderate enantio- and diastereo-selectivities usually without the need of an acidic or basic additive. In addition, these mesoporous materials could be reused and recycled with only a minor loss in their catalytic activity even after 15 or more cycles. The novel organocatalysts provide a significant advantage over other homogeneous and heterogeneous organocatalysts because they make viable sustainable aldol condensations with a drastic reduction of solvent usage.

## Figures and Tables

**Table 1:**
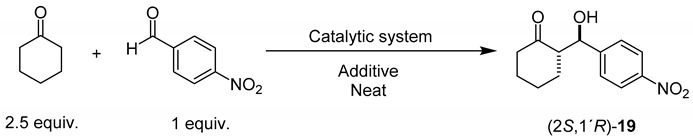
Asymmetric aldol reaction between cyclohexanone and 4-nitrobenzaldehyde in the presence of organocatalysts (*S*)**-5**, (*S*)**-12**, (2*S*,4*R*,1′*S*)**-18** and (2*S*,4*R*,1′*R*)**-18**.

Entry	Catalyst	Additive	Time (h)	*dr*^[c]^ (*anti*/*syn*)	*er*^[d]^ (*anti*/*syn*)	Yield % ^[e]^
**1**	Commercial silica	None	96	-	-	-
**2**	(S)-5 ^[a]^	None	20	78:21	65:35/60:40	98
**3**	(*S*)-**5** ^[a]^	H_2_O	24	74:25	70:30/65:35	99
**4**	(*S*)-**5** ^[a]^	*p*-nitro benzoic acid	24	70:30	85:15/60:40	99
**5**	(*S*)-**5** ^[a]^	salicylic acid	24	70:30	65:35/60:40	99
**6**	(*S*)-**5** ^[a,f]^	*p*-nitro-benzoic acid	36	76:24	96:4/70:30	99
**7**	(*S*)-**5** ^[a,f]^	salicylic acid	36	75:25	95:5/60:40	98
**8**	(*S*)-**12** ^[b]^	None	36	75:25	90:10/80:20	99
**9**	(*S*)-**12** ^[b]^	H_2_O	36	80:20	90:10/80:20	96
**10**	(*S*)-**12** ^[b,f]^	*p*-nitro- benzoic acid	36	70:30	90:10/60:40	96
**11**	(2*S,*4*R,*1′*S*)-**18** ^[a,f]^	None	22	70:30	65:35/65:30	96
**12**	(2*S,*4*R*,1′*R*)-**18** ^[a,f]^	None	24	70:30	60:40/60:40	97

^[a]^ An amount of 10 mol% of catalyst was employed; ^[b]^ 5 mol% of catalyst was employed. ^[c]^ The relative configuration was determined by ^1^H-NMR spectroscopy, *anti*: δ 4.90, *dd, J =* 8.4 Hz. *syn*: δ 5.47 ppm. ^[d]^ Absolute configuration was determined by HPLC analysis with an AD-H chiral column. ^[e]^ Following filtration. Extracted with 5 mL of EtOAc and purified in a small silica column after concentration. ^[f]^ At 1 °C. The aldehyde substrate is the limiting reagent, 37 mg (1.0 equiv.) of *p*-nitrobenzaldehyde. ^[g]^ Entry 1 is the control reaction; commercial silica was employed; the aldol reaction does not proceed in the absence of organocatalyst.

**Table 2:** Scope of the asymmetric aldol reaction between cyclohexanone and aromatic aldehydes catalyzed by (*S*)**-5**.

Entry	Product	R	Time (h)	*dr*^[a]^ (*anti*/*syn*)	*er*^[b]^ (for *anti* isomer)	Yield % ^[c]^
**1**	**20**	2-NO_2_	36	70:30	70:30	95
**2**	**21**	3-NO_2_	36	75:25	70:30	95
**3**	**22**	2-CF_3_	72	85:15	60:40	95
**4**	**23**	4-Cl	48	70:30	70:30	94
**5**	**24**	4-CF_3_	36	75:25	65:35	96
**6**	**25**	4-Br	36	80:20	80:20	99
**7**	**26**	-H	48	70:30	60:40	94

^[a]^ The relative configuration was assigned by analysis of ^1^H-NMR coupling constants. ^[b]^ The absolute configuration was determined by HPLC analysis with chiral columns (by comparison with literature data). ^[c]^ Determined after filtration, extraction with 5 mL of EtOAc, evaporation and purification through a small silica column. The aldehyde substrate is the limiting reagent: 37 mg (1.0 equiv.) of *p*-nitrobenzaldehyde.

**Table 3:**
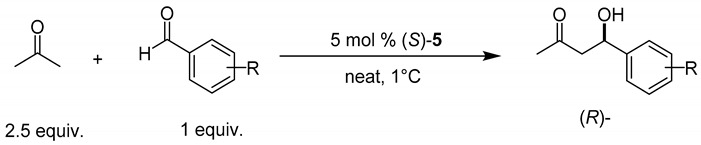
Asymmetric aldol reaction between acetone and aromatic aldehydes employing (*S*)-**5** as catalyst.

Entry	Product	R	Time (h)	*er* ^[a]^	Yield % ^[b]^
**1**	**27**	4-NO_2_	48	70:30	98
**2**	**28**	2-NO_2_	48	65:35	98
**3**	**29**	2-Cl	48	65:35	98
**4**	**30^c^**	4-CF_3_	36	60:40	98
**5**	**31**	4-Br	36	65:35	97
**6**	**32^c^**	4-Cl	72	70:30	97
**7**	**33**	4-F	48	65:35	99
**8**	**34**	-H	96	60:40	98
**9**	**35^c^**	4-CH_3_	96	60:40	98
**10**	**36^c^**	3-Br	48	60:40	99

^[a]^ Determined by HPLC analysis with chiral columns, by comparison with retention times reported in the literature. ^[b]^ Determined after filtration. Extracted with 5 mL of EtOAc and dried. ^[c]^ Determined after filtration. Extraction with 5 mL of EtOAc and purification by silica column chromatography.

**Table 4:**
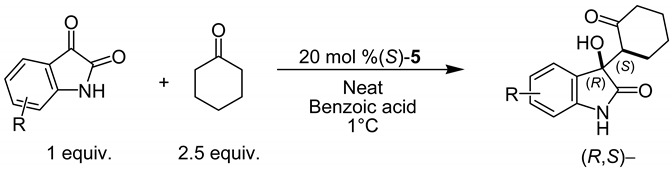
Asymmetric aldol reaction between isatin derivatives and aromatic aldehydes catalyzed by (*S*)**-5**.

Product	-R	Time (h)	*dr* (*anti*) ^[a]^	*er* (*anti*) ^[b]^	Yield % ^[c]^
**38**	-H	10	70:30	70:30	88
**39**	5-NO_2_	9	75:25	60:40	85
**40**	7-Cl	12	80:20	60:40	90

^[a]^ Determined by ^1^H-NMR spectroscopy. ^[b]^ Determined by HPLC analysis with chiral columns. ^[c]^ Determined after extraction with 5 mL of EtOAc, concentration and purification through a small silica column.

**Table 5:**
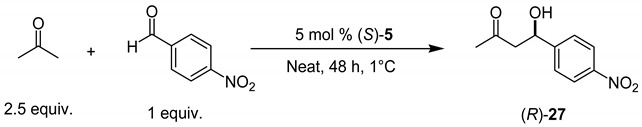
Asymmetric aldol reaction between acetone and aromatic aldehydes employing silica-supported (*S*)**-5** as organocatalyst.

Cycle	Er ^[a]^	Yield % ^[b]^
1	70:30	98
2	65:35	98
3	65:35	99
4	65:35	98
5	66:34	98
6	65:35	97
7	65:35	97
8	65:35	99
9	60:40	98
10	60:40	98
11	60:40	98
12	60:40	99
13	60:40	96
14	60:40	97
15	60:40	96

^[a]^ Determined by HPLC analysis with AS-H chiral columns. ^[b]^ Determined after filtration. Extracted with 5 mL of EtOAc and dried.

## Supplementary Materials

The following are available online. HPLC Analytical Data for aldol products, NMR Spectra for new and previous characterized compounds and aldol products, Gravimetric, TGA and DSG Analysis Data, and FR-IR.
