# Supplementary material for: New Mesoporous Silica-Supported Organocatalysts Based on (2S)-(1,2,4-Triazol-3-yl)-Proline: Efficient, Reusable, and Heterogeneous Catalysts for the Asymmetric Aldol Reaction
Source: Molecules. 2020 Oct 3;25(19):4532. doi: 10.3390/molecules25194532 (PMC7583865; doi:10.3390/molecules25194532)

New Mesoporous Silica-Supported Organocatalysts based on (2*S*)-(1,2,4-Triazol-3-yl)-proline: Efficient, Reusable, and Heterogeneous Catalysts for the Asymmetric Aldol Reaction.

Omar Sánchez-Antonio,[a] Kevin Romero-Sedglach, [a] Erika C. Vázquez-Orta,[a] and Eusebio Juaristi[a,b]

[a] *Omar Sánchez-Antonio, Kevin Romero-Sedglach, Erika C. Vázquez-Orta, and Eusebio Juaristi Departamento de Química. Centro de Investigación y de Estudios Avanzados del IPN., Av. IPN # 2508, 07360 Ciudad de México, México.*
E-mail: juaristi@relaq.mx or ejuarist@cinvestav.mx

[b] *Eusebio Juaristi. El Colegio Nacional, Luis González Obregón # 23, Centro Histórico, 06020, Ciudad de México, México.*

Table of contents

1. HPLC Analytical Data for aldol products…………………………………………………..S-2

2. NMR Spectra for new and previous characterized compounds and aldol products……….S-28

3. Gravimetric, TGA and DSG Analysis Data…………………………………………………..S-60

4. FR-IR…………………………………………………………………………………………..S-66

1. HPLC Analytical Data for aldol products.

a) (2*S*,1´*R*)-19 obtained with 4-nitrobenzoic acid as additive. Main product in a mixture of 4 diastereomers.

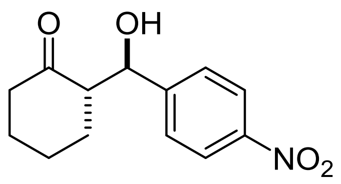

(2*S*,1´*R*)-**19**

Chromatogram and Results

Injection Details

Injection Name:	**KS-102C-1C**	*Run Time (min):*	**40.00**
Vial Number:	**RA1**	*Injection Volume:*	**20.00**
Injection Type:	**Unknown**	*Channel:*	**UV_VIS_1**
Calibration Level:		*Wavelength:*	**210.0**
Instrument Method:	**ADH 90 10 1 40**	*Bandwidth:*	**1**
Processing Method:	**EMP_aldolica**	*Dilution Factor:*	**1.0000**
Injection Date/Time:	**07/jul/18 09:57**	*Sample Weight:*	**1.0000**

Chromatogram

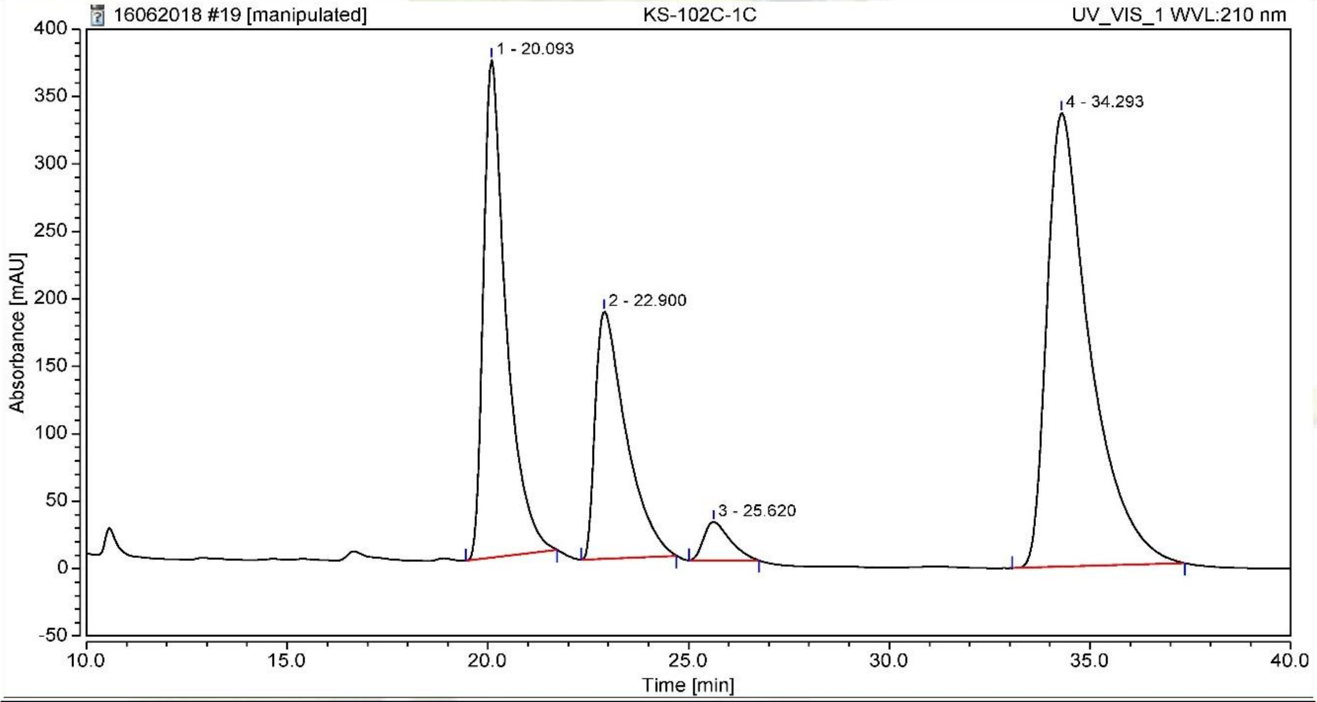

Integration Results

No.	Peak Name	Retention Time min	Area mAU*min	Height mAU	Relative Area %	Relative Height %	Amount n.a.
1		20.093	244.158	369.059	28.75	40.22	n.a.
2		22.900	163.268	183.440	19.22	19.99	n.a.
3		25.620	21.717	28.572	2.56	3.11	n.a.
4		34.293	420.124	336.523	49.47	36.67	n.a.
**Total:**			**849.266**	**917.593**	**100.00**	**100.00**	

(2*S*,1´*R*)-19 obtained with salicylic acid as additive. Main product in a mixture of 4 diastereomers.

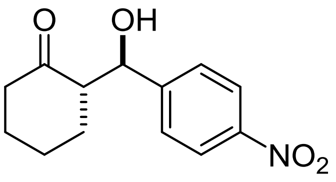

(2*S*,1´*R*)-**19**

Chromatogram and Results

Injection Details

Injection Name:	KS-102F-1C	*Run Time (min):*	40.00
Vial Number:	RA2	*Injection Volume:*	20.00
Injection Type:	Unknown	*Channel:*	UV_VIS_1
Calibration Level:		*Wavelength:*	210.0
Instrument Method:	ADH 90 10 1 40	*Bandwidth:*	2
Processing Method:	EMP_aldolica	*Dilution Factor:*	1.0000
Injection Date/Time:	07/jul/18 10:42	*Sample Weight:*	1.0000

Chromatogram

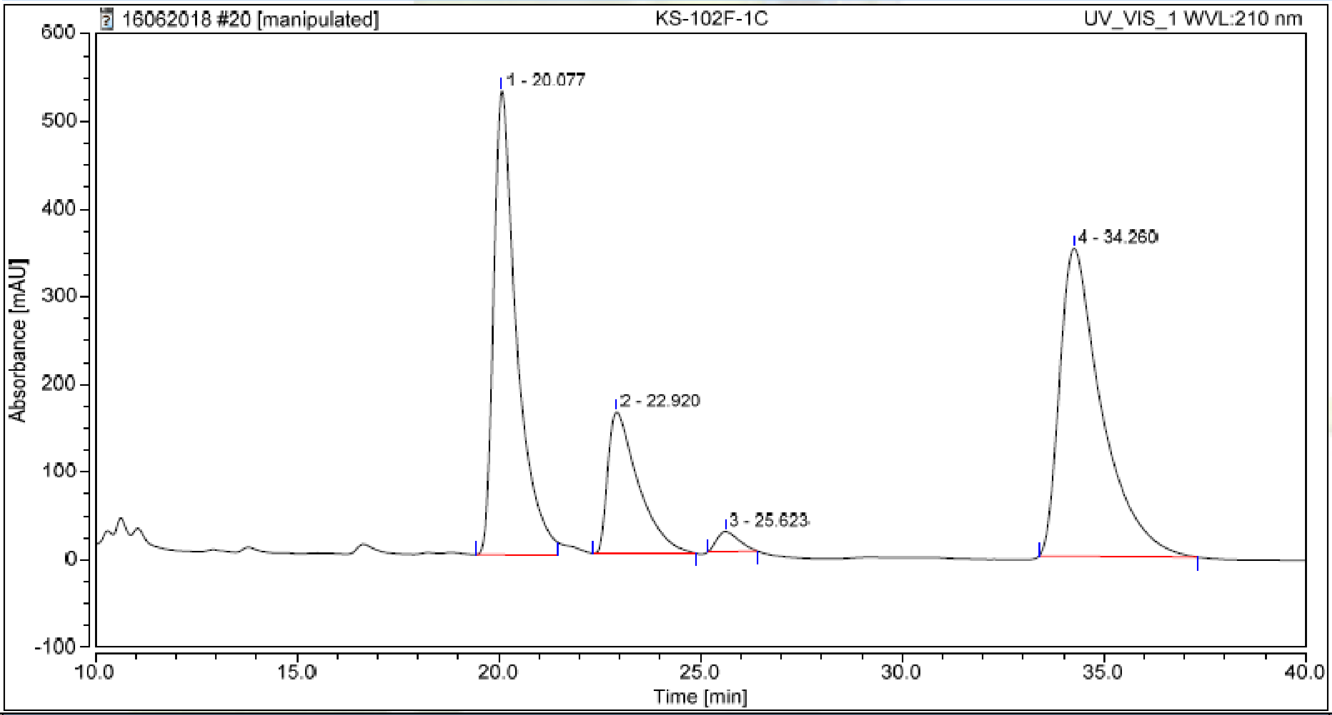

Integration Results

No.	Peak Name	Retention Time min	Area mAU*min	Height mAU	Relative Area %	Relative Height %	Amount n.a.
1		20.077	351.488	528.791	37.67	49.69	n.a.
2		22.920	141.418	161.002	15.16	15.13	n.a.
3		25.623	14.448	22.816	1.55	2.14	n.a.
4		34.260	425.788	351.545	45.63	33.04	n.a.
Total:			933.141	1064.155	100.00	100.00	

(2*S*,1´*R*)-20. Main product in a mixture of 4 diastereomers.

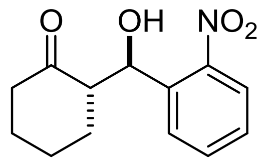

(2*S*,1´*R*)-**20**

Chromatogram and Results

Injection Details

Injection Name:	**KS-113A**	*Run Time (min):*	**45.00**
Vial Number:	**RA7**	*Injection Volume:*	**20.00**
Injection Type:	**Unknown**	*Channel:*	**UV_VIS_2**
Calibration Level:		*Wavelength:*	**210.0**
Instrument Method:	**ADH 95 05 10 45**	*Bandwidth:*	**2**
Processing Method:	**EMP_aldolica**	*Dilution Factor:*	**1.0000**
Injection Date/Time:	**07/jul/18 14:55**	*Sample Weight:*	**1.0000**

Chromatogram

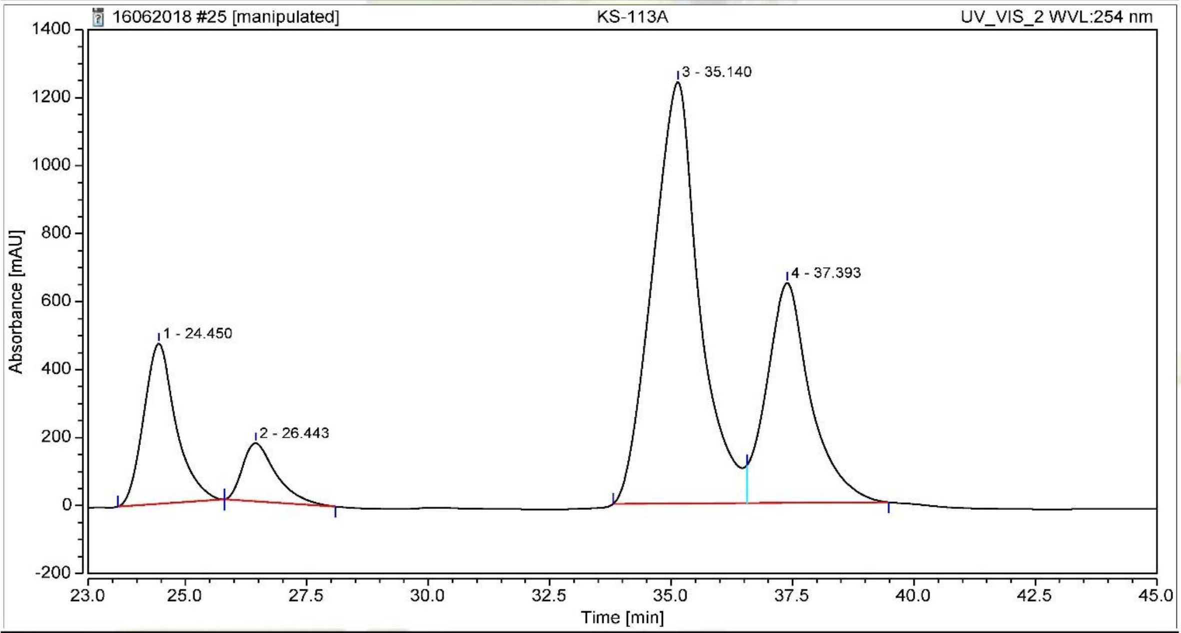

Integration Results

No.	Peak Name	Retention Time min	Area mAU*min	Height mAU	Relative Area %	Relative Height %	Amount n.a.
1		24.450	363.282	471.391	14.18	18.64	n.a.
2		26.443	137.463	171.152	5.37	6.77	n.a.
3		35.140	1385.358	1239.792	54.07	49.01	n.a.
4		37.393	675.895	647.084	26.38	25.58	n.a.
**Total:**			**2561.998**	**2529.419**	**100.00**	**100.00**	

(2*S*,1´*R*)-21. Main product in a mixture of 4 diastereomers.

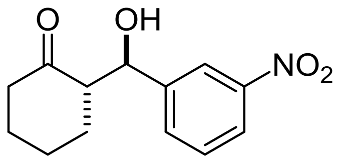

(2*S*,1´*R*)-**21**

Chromatogram and Results

Injection Details

Injection Name:	**KS-113B-02 MX**	*Run Time (min):*	**70.00**
Vial Number:	**RA7**	*Injection Volume:*	**20.00**
Injection Type:	**Unknown**	*Channel:*	**UV_VIS_2**
Calibration Level:		*Wavelength:*	**210.0**
Instrument Method:	**ADH 95 05 08 65**	*Bandwidth:*	**1**
Processing Method:	**EMP_aldolica**	*Dilution Factor:*	**1.0000**
Injection Date/Time:	**03/ago/18 16:16**	*Sample Weight:*	**1.0000**

Chromatogram

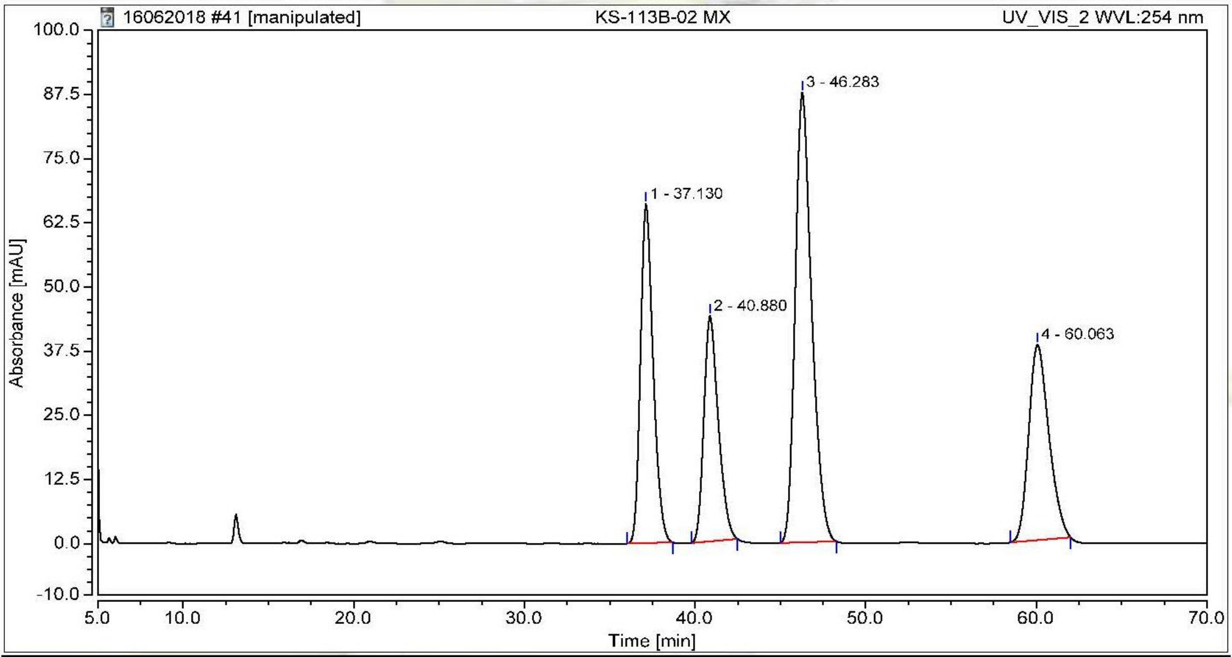

Integration Results

No.	Peak Name	Retention Time min	Area mAU*min	Height mAU	Relative Area %	Relative Height %	Amount n.a.
1		37.130	56.741	66.025	22.72	28.02	n.a.
2		40.880	42.964	43.879	17.20	18.62	n.a.
3		46.283	97.907	87.639	39.20	37.19	n.a.
4		60.063	52.120	38.092	20.87	16.17	n.a.
**Total:**			**249.733**	**235.634**	**100.00**	**100.00**	

(2*S*,1´*R*)-22. Main product in a mixture of 4 diastereomers.

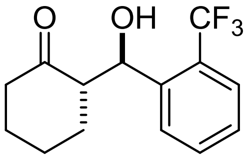

(2*S*,1´*R*)-**22**

Instrument:LABORATORIO_19 Sequence:16062018 Page 1 of 1

Chromatogram and Results

Injection Details

Injection Name:	**KS-113C**	*Run Time (min):*	**40.00**
Vial Number:	**RB3**	*Injection Volume:*	**20.00**
Injection Type:	**Unknown**	*Channel:*	**UV_VIS_1**
Calibration Level:		*Wavelength:*	**210.0**
Instrument Method:	**ADH 90 10 05 45**	*Bandwidth:*	**1**
Processing Method:	**EMP_aldolica**	*Dilution Factor:*	**1.0000**
Injection Date/Time:	**13/ago/18 08:35**	*Sample Weight:*	**1.0000**

Chromatogram

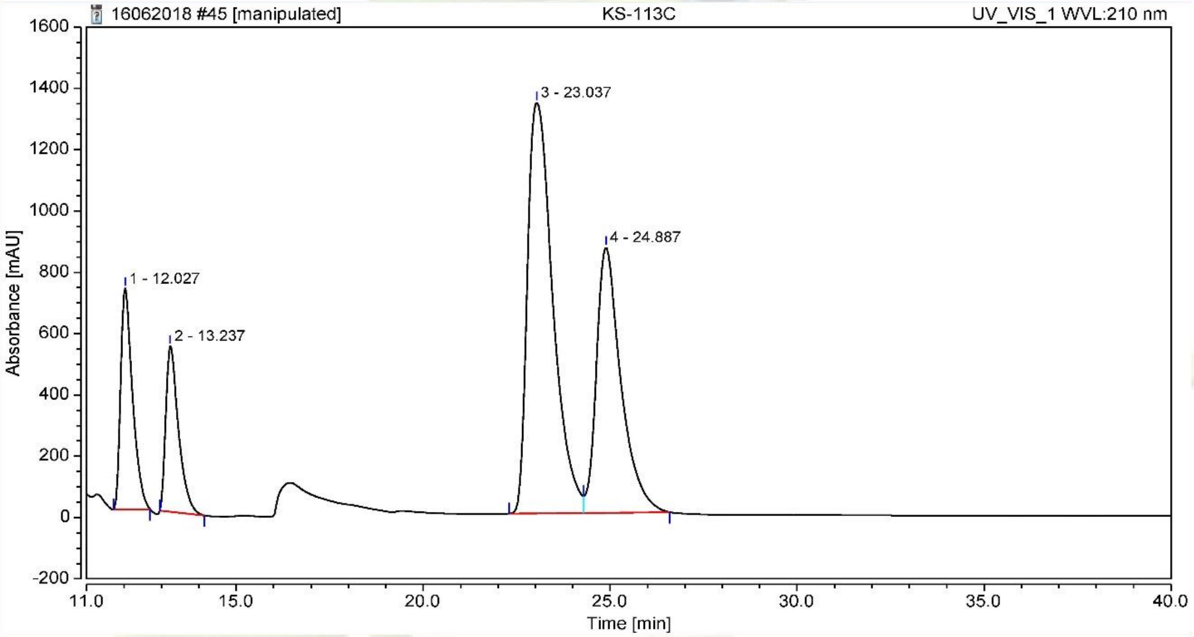

Integration Results

No.	Peak Name	Retention Time min	Area mAU*min	Height mAU	Relative Area %	Relative Height %	Amount n.a.
1		12.027	266.412	721.277	12.16	20.80	n.a.
2		13.237	212.580	540.402	9.70	15.58	n.a.
3		23.037	1054.251	1339.854	48.13	38.64	n.a.
4		24.887	657.224	866.166	30.00	24.98	n.a.
**Total:**			**2190.466**	**3467.699**	**100.00**	**100.00**	

(2*S*,1´*R*)-23. Main product in a mixture of 4 diastereomers.

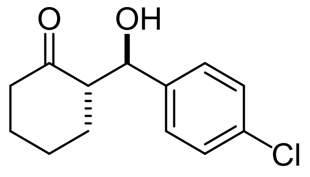

(2*S*,1´*R*)-**23**

Chromatogram and Results

Injection Details

Injection Name:	**4CL**	*Run Time (min):*	**30.00**
Vial Number:	**GB8**	*Injection Volume:*	**20.00**
Injection Type:	**Unknown**	*Channel:*	**UV_VIS_1**
Calibration Level:		*Wavelength:*	**210.0**
Instrument Method:	**AD-H 9010 05 30**	*Bandwidth:*	**2**
Processing Method:	**EMP_aldolica**	*Dilution Factor:*	**1.0000**
Injection Date/Time:	**21/feb/18 18:55**	*Sample Weight:*	**1.0000**

Chromatogram

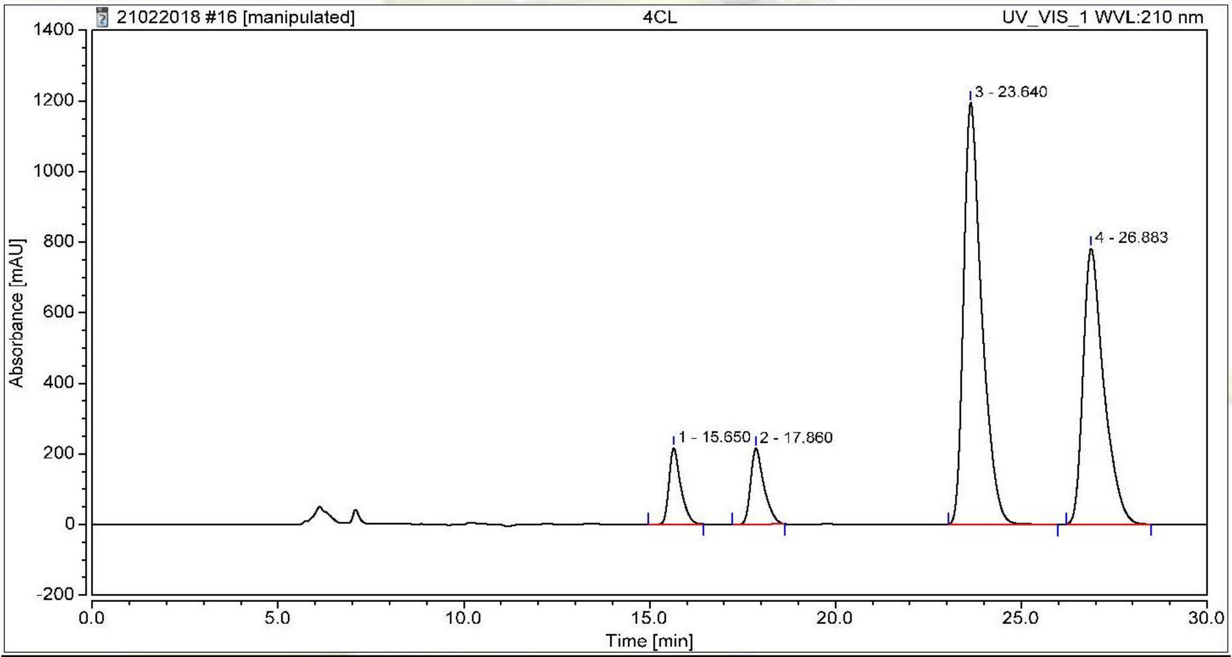

Integration Results

No.	Peak Name	Retention Time min	Area mAU*min	Height mAU	Relative Area %	Relative Height %	Amount n.a.
1		15.650	78.596	217.023	5.82	9.00	n.a.
2		17.860	89.313	215.612	6.61	8.94	n.a.
3		23.640	677.368	1195.207	60.12	59.58	n.a.
4		26.883	506.246	782.718	27.46	22.47	n.a.
**Total:**			**1351.522**	**2410.560**	**100.00**	**100.00**	

(2*S*,1´*R*)-24. Main product in a mixture of 4 diastereomers.

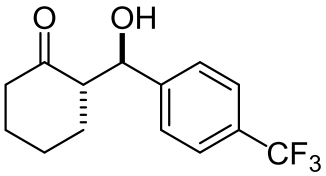

(2*S*,1´*R*)-**24**

Instrument:LABORATORIO_19 Sequence:16062018 Page 1 of 1

Chromatogram and Results

Injection Details

Injection Name:	**KS-113E**	*Run Time (min):*	**40.00**
Vial Number:	**RB6**	*Injection Volume:*	**20.00**
Injection Type:	**Unknown**	*Channel:*	**UV_VIS_1**
Calibration Level:		*Wavelength:*	**210.0**
Instrument Method:	**ADH 90 10 05 45**	*Bandwidth:*	**1**
Processing Method:	**EMP_aldolica**	*Dilution Factor:*	**1.0000**
Injection Date/Time:	**13/ago/18 09:20**	*Sample Weight:*	**1.0000**

Chromatogram

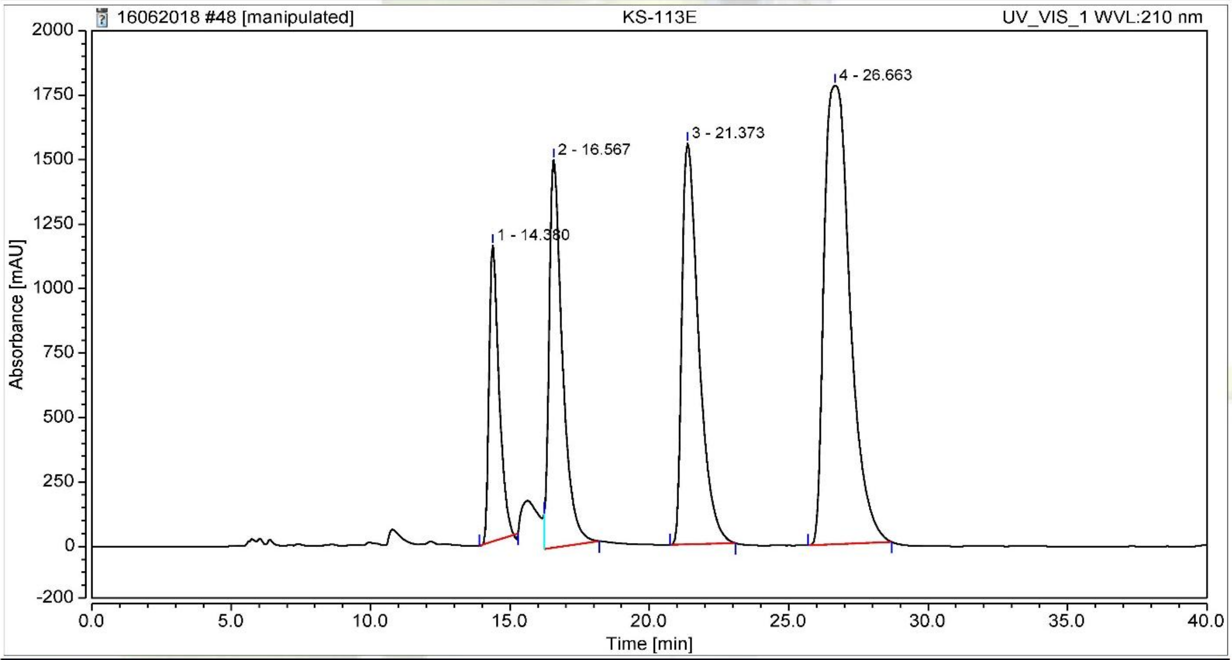

Integration Results

No.	Peak Name	Retention Time min	Area mAU*min	Height mAU	Relative Area %	Relative Height %	Amount n.a.
1		14.380	473.040	1148.085	10.94	19.18	n.a.
2		16.567	833.035	1503.527	19.27	25.11	n.a.
3		21.373	1092.995	1555.883	25.28	25.99	n.a.
4		26.663	1924.795	1779.289	44.52	29.72	n.a.
**Total:**			**4323.867**	**5986.784**	**100.00**	**100.00**	

(2*S*,1´*R*)-25. Main product in a mixture of 4 diastereomers.

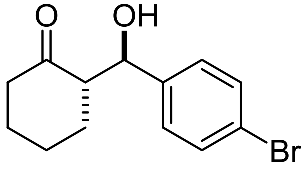

(2*S*,1´*R*)-**25**

Instrument:LABORATORIO_19 Sequence:16062018 Page 1 of 1

Chromatogram and Results

Injection Details

Injection Name:	**KS-113F**	*Run Time (min):*	**40.00**
Vial Number:	**RB4**	*Injection Volume:*	**20.00**
Injection Type:	**Unknown**	*Channel:*	**UV_VIS_1**
Calibration Level:		*Wavelength:*	**210.0**
Instrument Method:	**ADH 90 10 05 45**	*Bandwidth:*	**1**
Processing Method:	**EMP_aldolica**	*Dilution Factor:*	**1.0000**
Injection Date/Time:	**11/ago/18 10:54**	*Sample Weight:*	**1.0000**

Chromatogram

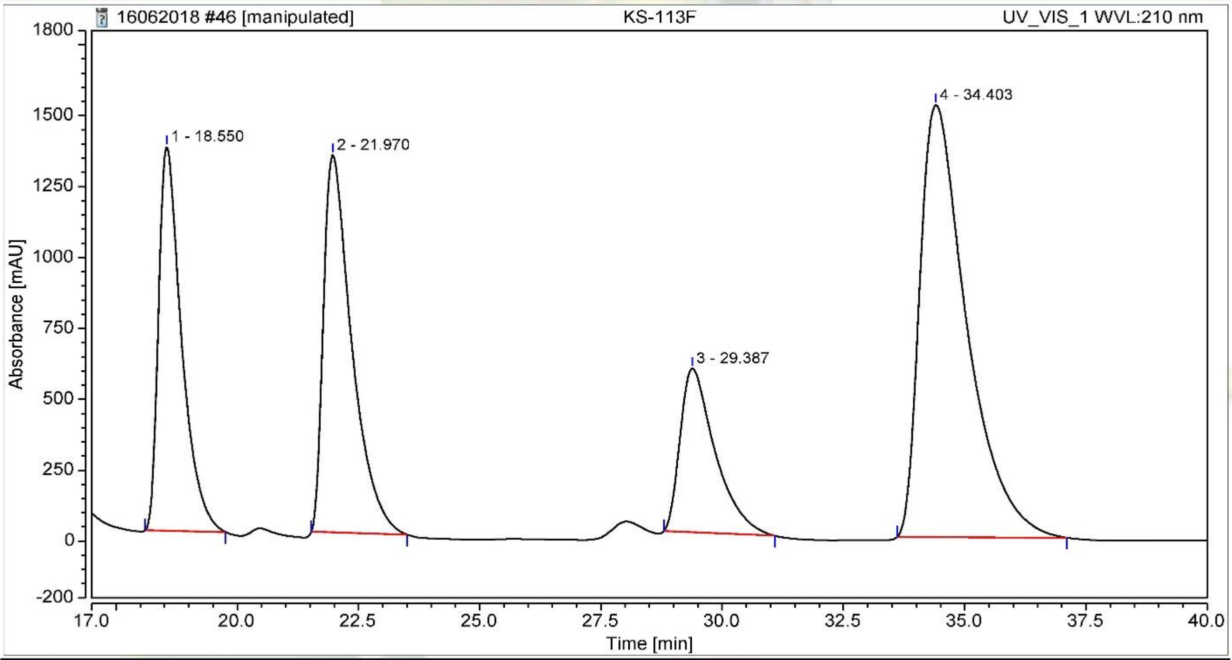

Integration Results

No.	Peak Name	Retention Time min	Area mAU*min	Height mAU	Relative Area %	Relative Height %	Amount n.a.
1		18.550	745.507	1354.396	19.43	28.30	n.a.
2		21.970	900.633	1330.649	23.48	27.80	n.a.
3		29.387	482.442	577.445	12.58	12.06	n.a.
4		34.403	1707.442	1524.083	44.51	31.84	n.a.
**Total:**			**3836.024**	**4786.574**	**100.00**	**100.00**	

(2*S*,1´*R*)-26. Main product in a mixture of 4 diastereomers.

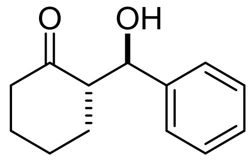

(2*S*,1´*R*)-**26**

Instrument:LABORATORIO_19 Sequence:16062018 Page 1 of

Chromatogram and Results

Injection Details

Injection Name:	**KS-113H**	*Run Time (min):*	**40.00**
Vial Number:	**RA2**	*Injection Volume:*	**20.00**
Injection Type:	**Unknown**	*Channel:*	**UV_VIS_1**
Calibration Level:		*Wavelength:*	**210.0**
Instrument Method:	**ODH 95 05 05 40**	*Bandwidth:*	**1**
Processing Method:	**EMP_aldolica**	*Dilution Factor:*	**1.0000**
Injection Date/Time:	**13/ago/18 14:56**	*Sample Weight:*	**1.0000**

Chromatogram

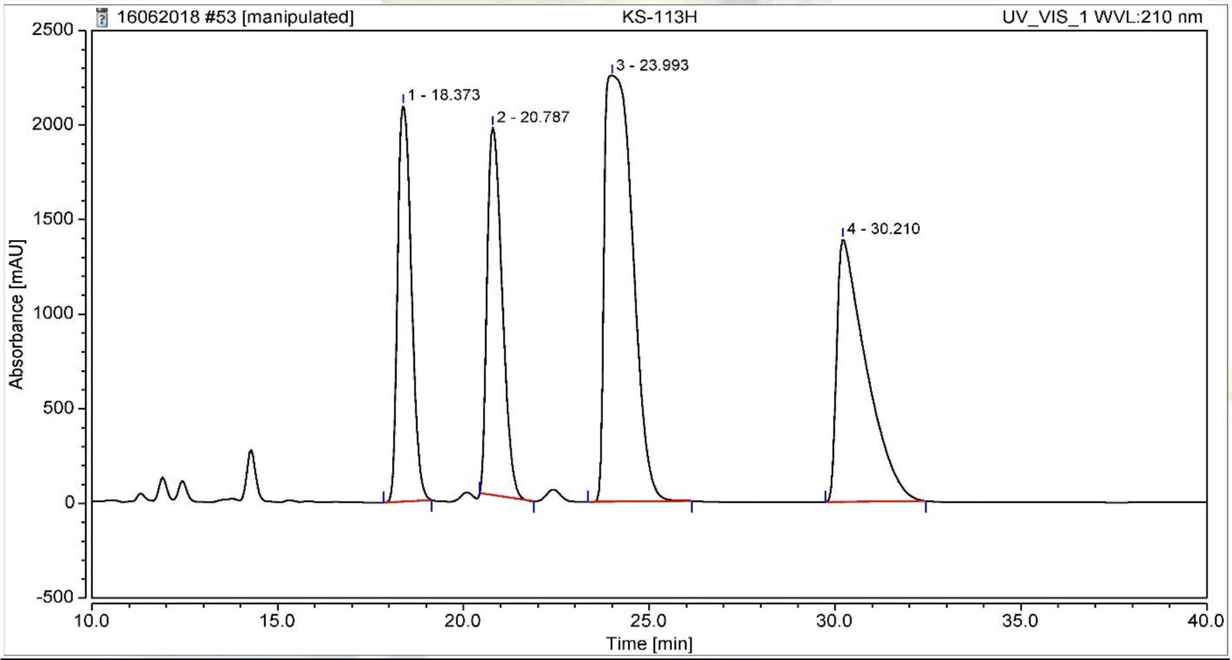

Integration Results

No.	Peak Name	Retention Time min	Area mAU*min	Height mAU	Relative Area %	Relative Height %	Amount n.a.
1		18.373	899.091	2094.422	17.76	27.28	n.a.
2		20.787	911.433	1941.520	18.00	25.29	n.a.
3		23.993	1980.299	2252.379	39.11	29.34	n.a.
4		30.210	1272.801	1389.585	25.14	18.10	n.a.
**Total:**			**5063.624**	**7677.906**	**100.00**	**100.00**	

b) Aldol products obtained with acetone and aryl aldehydes.

(*R*)-27. Main product in a mixture of enantiomers.

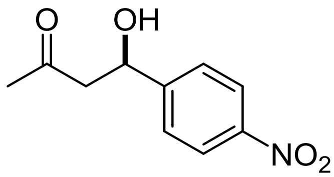

(*R*)-**27**

Instrument:LABORATORIO_19 Sequence:23062018 Page 1 of 1

Chromatogram and Results

Injection Details

Injection Name:	**KS-110A**	*Run Time (min):*	**30.00**
Vial Number:	**RA1**	*Injection Volume:*	**20.00**
Injection Type:	**Unknown**	*Channel:*	**UV_VIS_2**
Calibration Level:		*Wavelength:*	**210.0**
Instrument Method:	**ASH 80 20 1 30**	*Bandwidth:*	**2**
Processing Method:	**EMP_aldolica**	*Dilution Factor:*	**1.0000**
Injection Date/Time:	**12/jul/18 12:57**	*Sample Weight:*	**1.0000**

Chromatogram

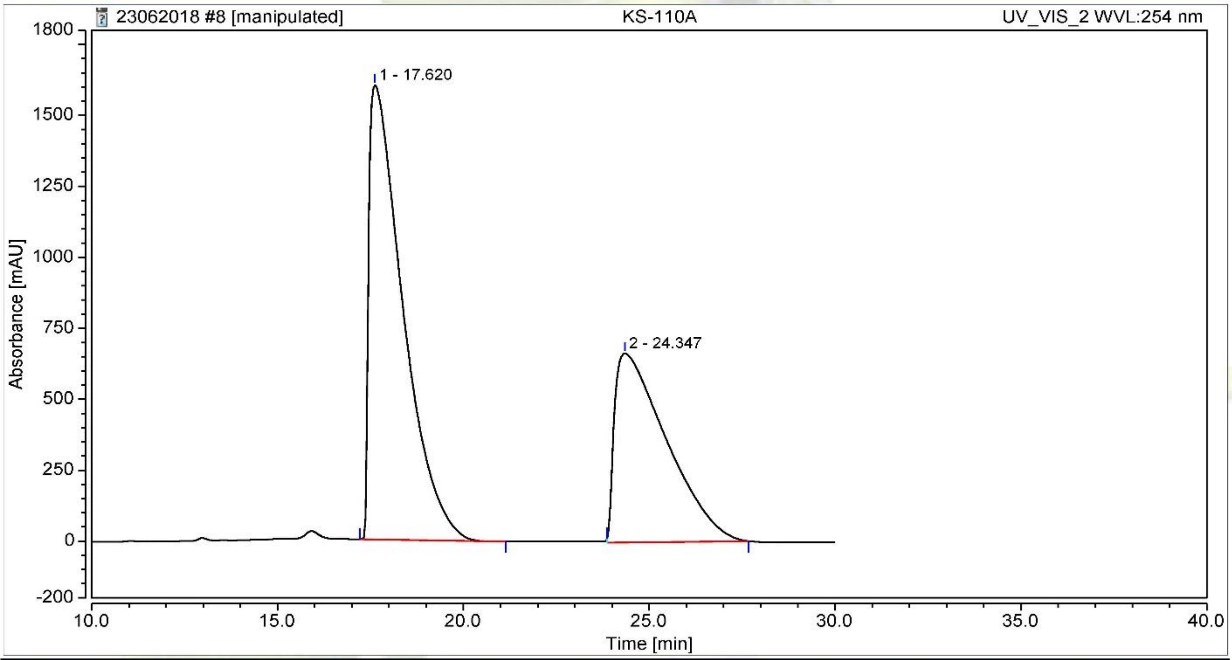

Integration Results

No.	Peak Name	Retention Time min	Area mAU*min	Height mAU	Relative Area %	Relative Height %	Amount n.a.
1		17.620	1691.654	1600.961	71.09	70.61	n.a.
2		24.347	1077.616	666.274	28.91	29.39	n.a.
**Total:**			**2769.270**	**2267.235**	**100.00**	**100.00**	

(*R*)-28. Main product in a mixture of enantiomers.

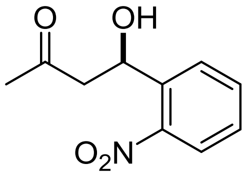

(*R*)-**28**

Chromatogram and Results

Injection Details

Injection Name:	**KS-110B-EE**	*Run Time (min):*	**30.00**
Vial Number:	**RA2**	*Injection Volume:*	**20.00**
Injection Type:	**Unknown**	*Channel:*	**UV_VIS_2**
Calibration Level:		*Wavelength:*	**210.0**
Instrument Method:	**ASH 70 30 1 25**	*Bandwidth:*	**4**
Processing Method:	**EMP_aldolica**	*Dilution Factor:*	**1.0000**
Injection Date/Time:	**04/sep/18 19:32**	*Sample Weight:*	**1.0000**

Chromatogram

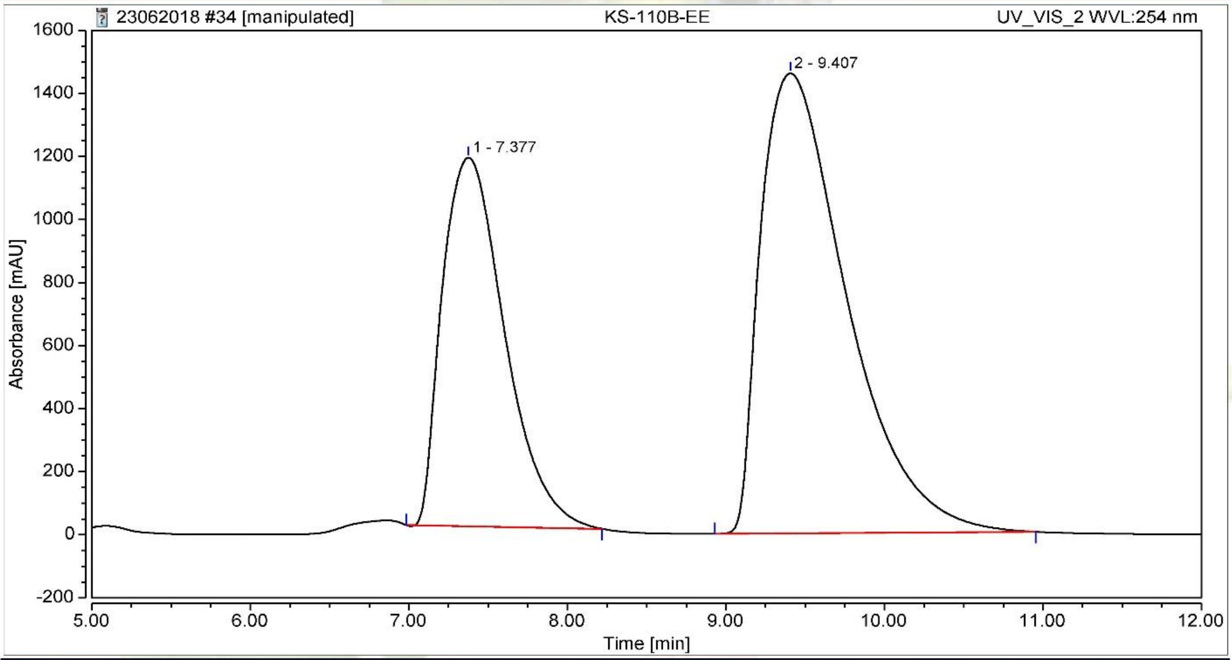

Integration Results

No.	Peak Name	Retention Time min	Area mAU*min	Height mAU	Relative Area %	Relative Height %	Amount n.a.
1		7.377	533.791	1169.967	36.71	44.49	n.a.
2		9.407	920.173	1459.897	63.29	55.51	n.a.
**Total:**			**1453.964**	**2629.864**	**100.00**	**100.00**	

(*R*)-29. Main product in a mixture of enantiomers.

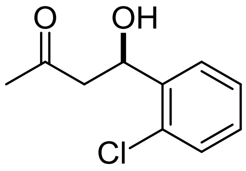

(*R*)-**29**

Chromatogram and Results

Injection Details

Injection Name:	**KS-110D-EE**	*Run Time (min):*	**25.00**
Vial Number:	**RA3**	*Injection Volume:*	**20.00**
Injection Type:	**Unknown**	*Channel:*	**UV_VIS_1**
Calibration Level:		*Wavelength:*	**210.0**
Instrument Method:	**ASH 92 08 10 25**	*Bandwidth:*	**1**
Processing Method:	**EMP_aldolica**	*Dilution Factor:*	**1.0000**
Injection Date/Time:	**04/sep/18 20:07**	*Sample Weight:*	**1.0000**

Chromatogram

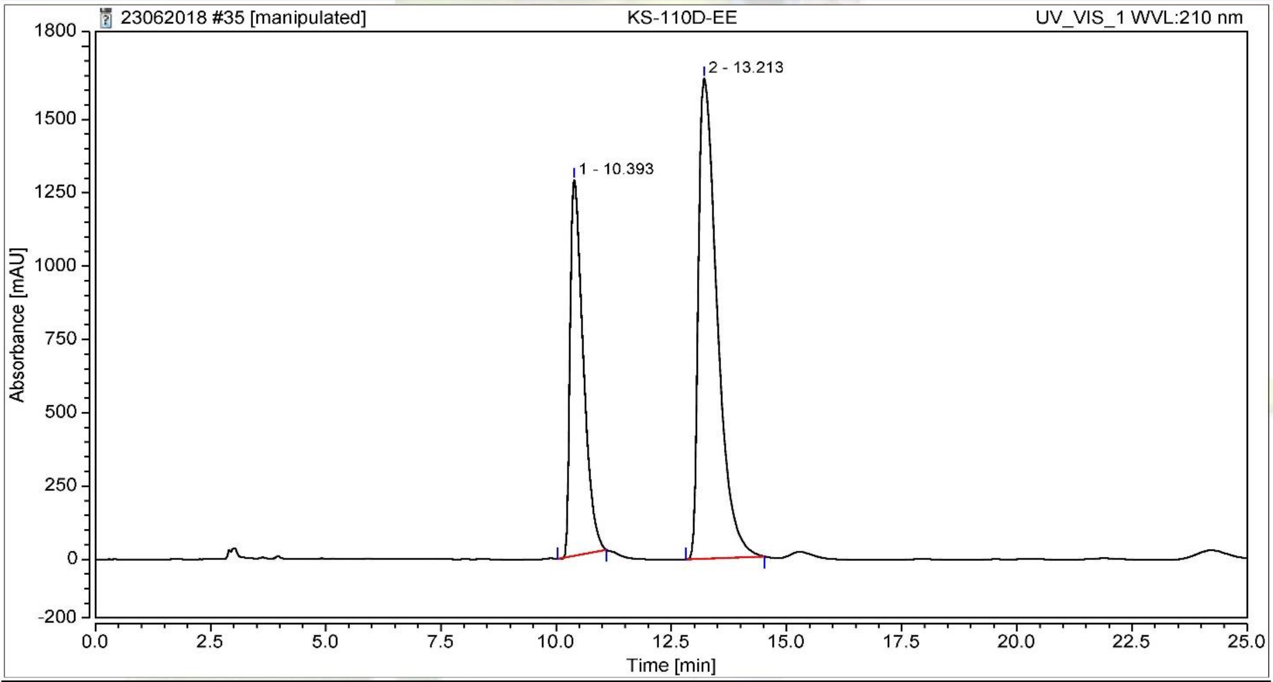

Integration Results

No.	Peak Name	Retention Time min	Area mAU*min	Height mAU	Relative Area %	Relative Height %	Amount n.a.
1		10.393	421.775	1282.369	35.23	43.90	n.a.
2		13.213	775.544	1638.684	64.77	56.10	n.a.
**Total:**			**1197.319**	**2921.053**	**100.00**	**100.00**	

(*R*)-30. Main product in a mixture of enantiomers.

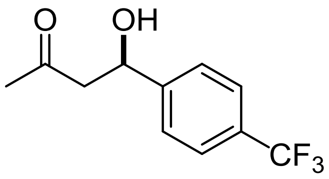

(*R*)-**30**

Chromatogram and Results

Injection Details

Injection Name:	**KS-110F-EE**	*Run Time (min):*	**20.00**
Vial Number:	**RA2**	*Injection Volume:*	**20.00**
Injection Type:	**Unknown**	*Channel:*	**UV_VIS_1**
Calibration Level:		*Wavelength:*	**210.0**
Instrument Method:	**ASH 90 10 10 20**	*Bandwidth:*	**2**
Processing Method:	**EMP_aldolica**	*Dilution Factor:*	**1.0000**
Injection Date/Time:	**06/sep/18 19:10**	*Sample Weight:*	**1.0000**

Chromatogram

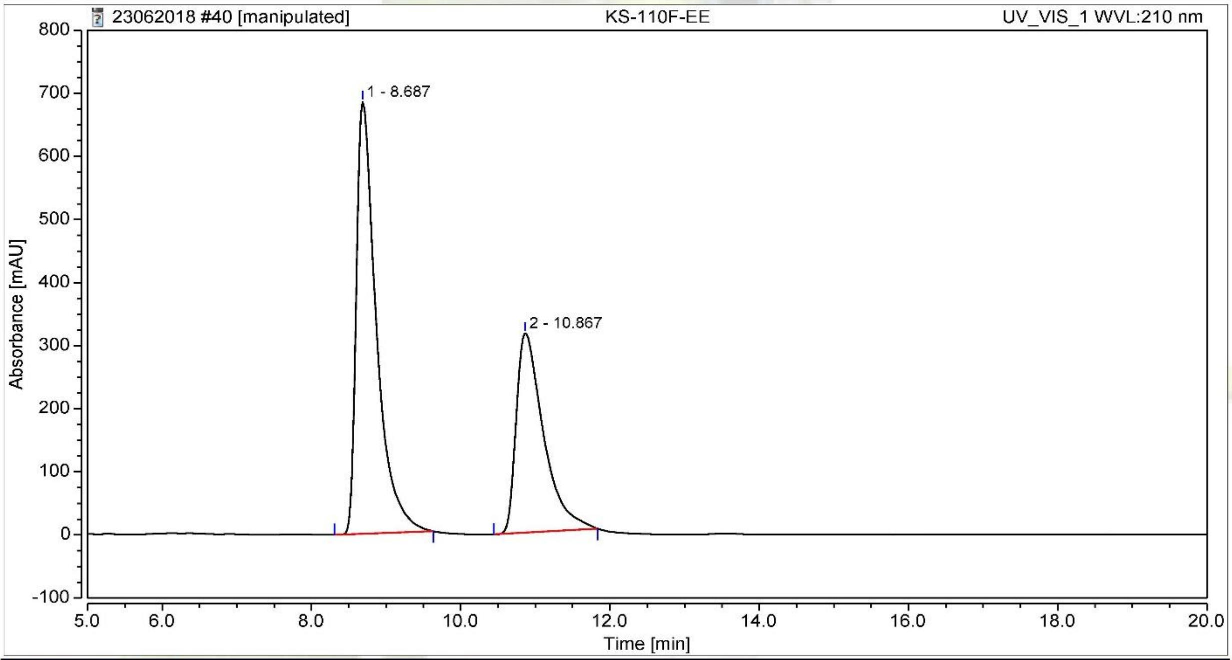

Integration Results

No.	Peak Name	Retention Time min	Area mAU*min	Height mAU	Relative Area %	Relative Height %	Amount n.a.
1		8.687	213.341	684.511	61.48	68.41	n.a.
2		10.867	133.672	316.068	38.52	31.59	n.a.
**Total:**			**347.013**	**1000.579**	**100.00**	**100.00**	

(*R*)-31. Main product in a mixture of enantiomers.

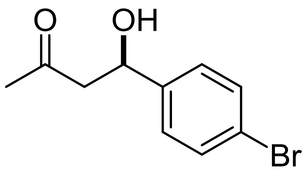

(*R*)-**31**

Chromatogram and Results

Injection Details

Injection Name:	**KS-110L-EE-00**	*Run Time (min):*	**40.00**
Vial Number:	**GC3**	*Injection Volume:*	**20.00**
Injection Type:	**Unknown**	*Channel:*	**UV_VIS_1**
Calibration Level:		*Wavelength:*	**210.0**
Instrument Method:	**ASH 90 10 05 45**	*Bandwidth:*	**1**
Processing Method:	**EMP_aldolica**	*Dilution Factor:*	**1.0000**
Injection Date/Time:	**03/oct/18 11:52**	*Sample Weight:*	**1.0000**

Chromatogram

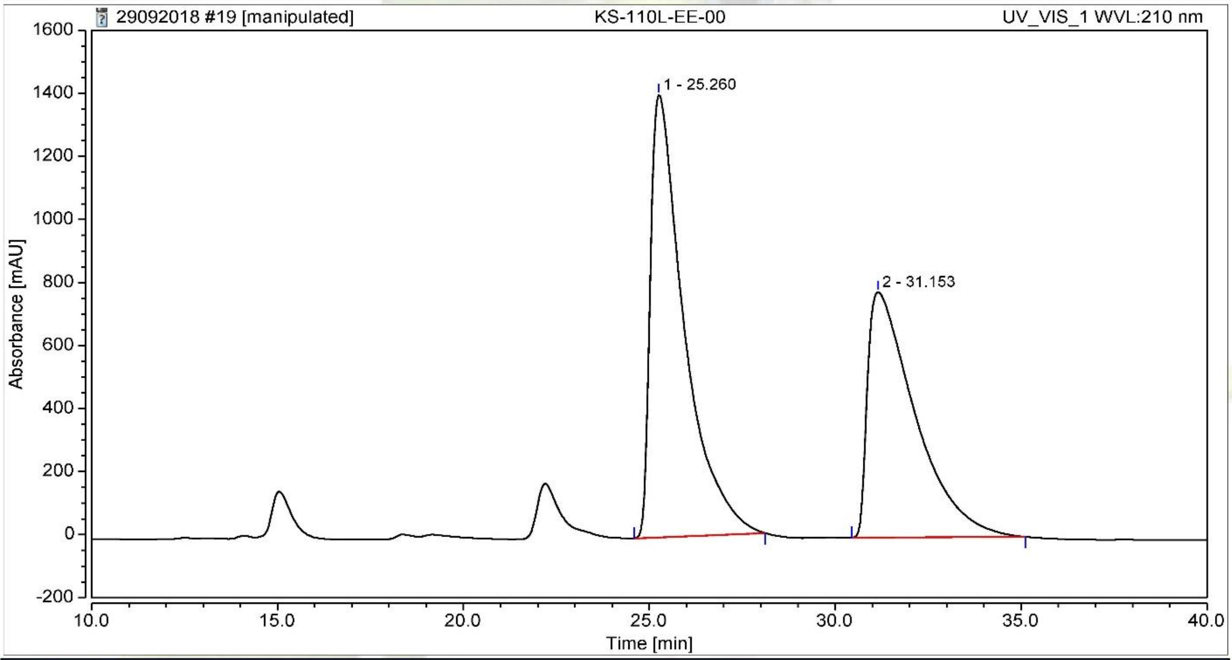

Integration Results

No.	Peak Name	Retention Time min	Area mAU*min	Height mAU	Relative Area %	Relative Height %	Amount n.a.
1		25.260	1477.992	1404.387	56.12	64.32	n.a.
2		31.153	1155.767	779.148	43.88	35.68	n.a.
**Total:**			**2633.759**	**2183.535**	**100.00**	**100.00**	

(*R*)-32. Main product in a mixture of enantiomers.

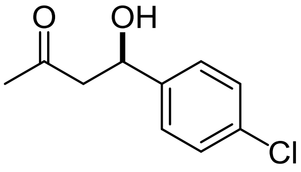

(*R*)-**32**

Chromatogram and Results

Injection Details

Injection Name:	**KS-110E-EE-02**	*Run Time (min):*	**20.00**
Vial Number:	**GA3**	*Injection Volume:*	**20.00**
Injection Type:	**Unknown**	*Channel:*	**UV_VIS_1**
Calibration Level:		*Wavelength:*	**210.0**
Instrument Method:	**ASH 90 10 10 20**	*Bandwidth:*	**2**
Processing Method:	**EMP_aldolica**	*Dilution Factor:*	**1.0000**
Injection Date/Time:	**19/sep/18 11:40**	*Sample Weight:*	**1.0000**

Chromatogram

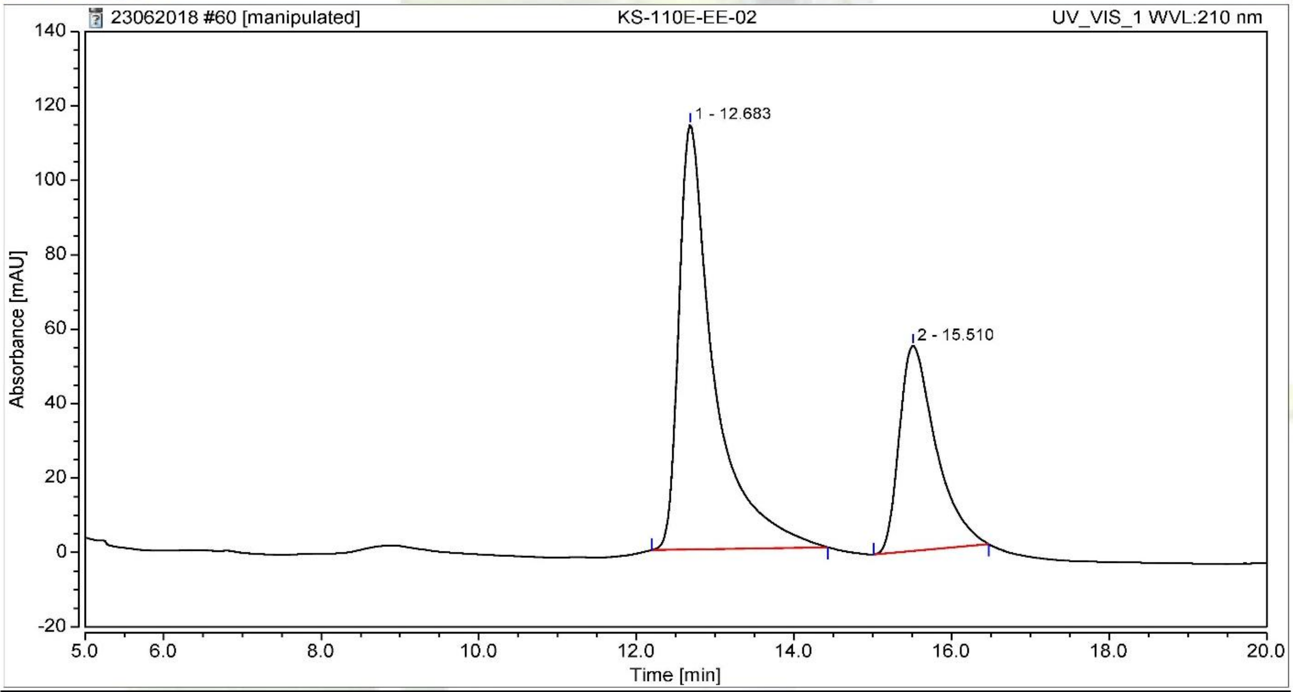

Integration Results

No.	Peak Name	Retention Time min	Area mAU*min	Height mAU	Relative Area %	Relative Height %	Amount n.a.
1		12.683	59.122	114.205	71.57	67.43	n.a.
2		15.510	29.691	55.161	29.43	32.57	n.a.
**Total:**			**88.812**	**169.366**	**100.00**	**100.00**	

(*R*)-33. Main product in a mixture of enantiomers.

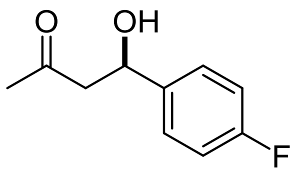

(*R*)-**33**

Chromatogram and Results

Injection Details

Injection Name:	**KS-110H-EE**	*Run Time (min):*	**20.00**
Vial Number:	**RA4**	*Injection Volume:*	**20.00**
Injection Type:	**Unknown**	*Channel:*	**UV_VIS_1**
Calibration Level:		*Wavelength:*	**210.0**
Instrument Method:	**ASH 90 10 10 20**	*Bandwidth:*	**2**
Processing Method:	**EMP_aldolica**	*Dilution Factor:*	**1.0000**
Injection Date/Time:	**06/sep/18 19:54**	*Sample Weight:*	**1.0000**

Chromatogram

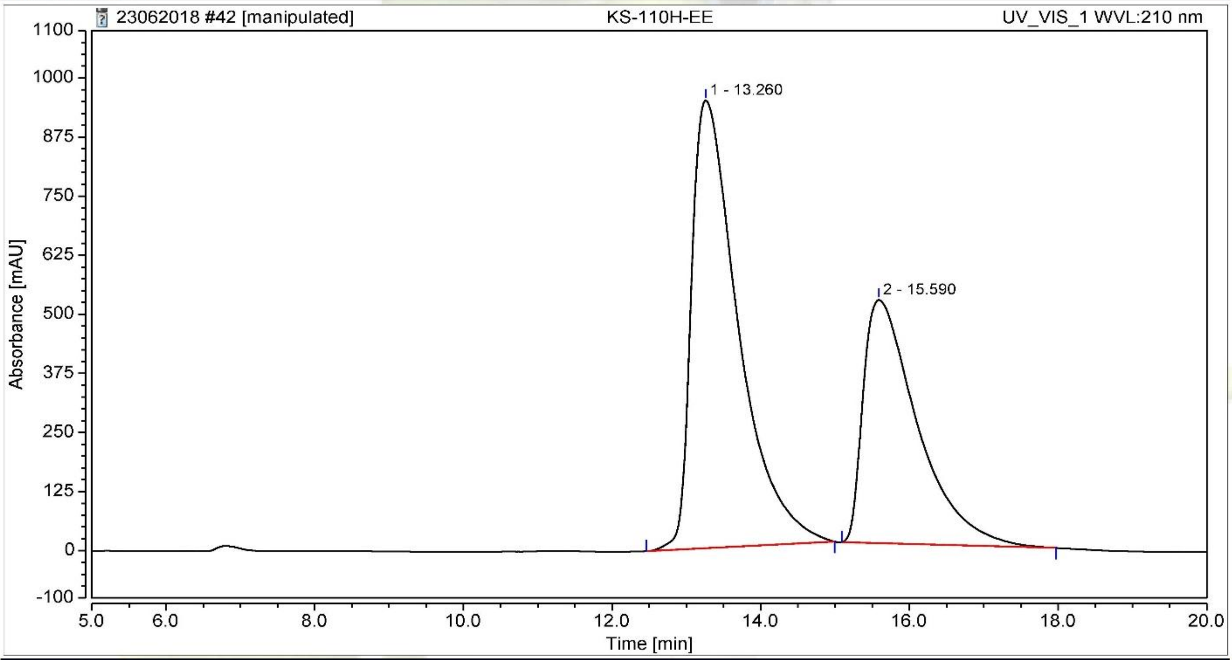

Integration Results

No.	Peak Name	Retention Time min	Area mAU*min	Height mAU	Relative Area %	Relative Height %	Amount n.a.
1		13.260	691.349	947.951	61.40	64.80	n.a.
2		15.590	434.711	514.986	38.60	35.20	n.a.
**Total:**			**1126.061**	**1462.937**	**100.00**	**100.00**	

(*R*)-34. Main product in a mixture of enantiomers.

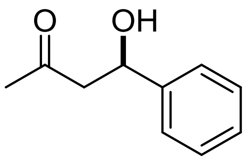

(*R*)-**34**

Chromatogram and Results

Injection Details

Injection Name:	**KS-110I-EE-00**	*Run Time (min):*	**25.00**
Vial Number:	**GB2**	*Injection Volume:*	**20.00**
Injection Type:	**Unknown**	*Channel:*	**UV_VIS_1**
Calibration Level:		*Wavelength:*	**210.0**
Instrument Method:	**ASH 90 10 10 25**	*Bandwidth:*	**2**
Processing Method:	**EMP_aldolica**	*Dilution Factor:*	**1.0000**
Injection Date/Time:	**29/sep/18 20:06**	*Sample Weight:*	**1.0000**

Chromatogram

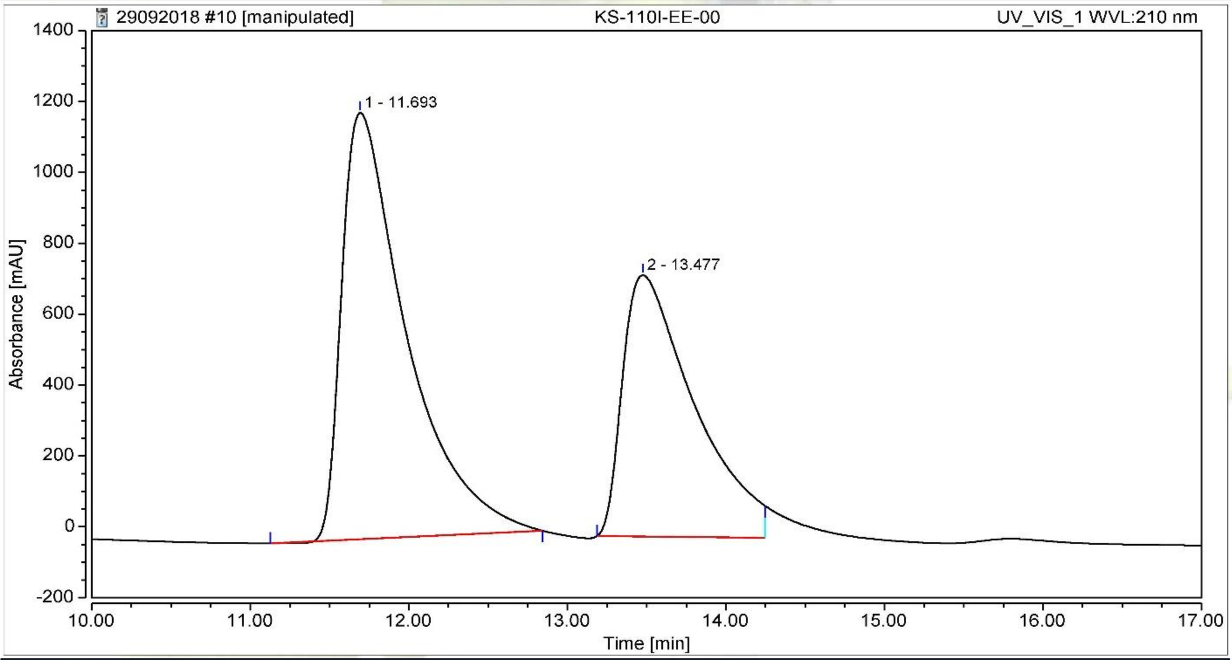

Integration Results

No.	Peak Name	Retention Time min	Area mAU*min	Height mAU	Relative Area %	Relative Height %	Amount n.a.
1		11.693	567.907	1203.351	59.66	62.00	n.a.
2		13.477	383.967	737.688	40.34	38.00	n.a.
**Total:**			**951.874**	**1941.039**	**100.00**	**100.00**	

(*R*)-35. Main product in a mixture of enantiomers.

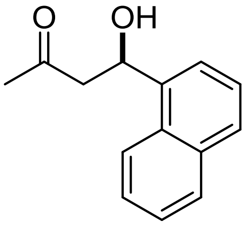

(*R*)-**35**

Chromatogram and Results

Injection Details

Injection Name:	**KS-110K-EE-00**	*Run Time (min):*	**25.00**
Vial Number:	**GC2**	*Injection Volume:*	**20.00**
Injection Type:	**Unknown**	*Channel:*	**UV_VIS_1**
Calibration Level:		*Wavelength:*	**210.0**
Instrument Method:	**ASH 90 10 10 25**	*Bandwidth:*	**1**
Processing Method:	**EMP_aldolica**	*Dilution Factor:*	**1.0000**
Injection Date/Time:	**03/oct/18 11:22**	*Sample Weight:*	**1.0000**

Chromatogram

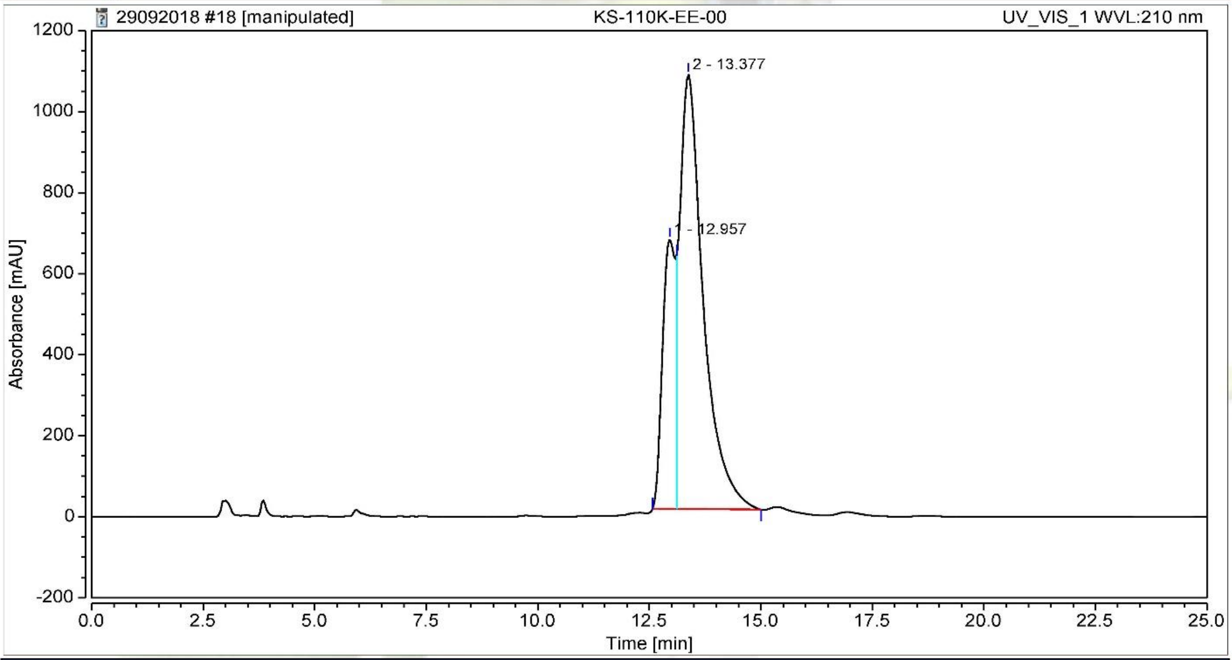

Integration Results

No.	Peak Name	Retention Time min	Area mAU*min	Height mAU	Relative Area %	Relative Height %	Amount n.a.
1		12.957	222.693	664.891	25.42	38.27	n.a.
2		13.377	653.267	1072.565	74.58	61.73	n.a.
**Total:**			**875.960**	**1737.457**	**100.00**	**100.00**	

***rac*-36, reference sample.**

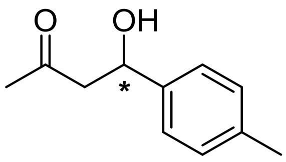

rac-**36**

Chromatogram and Results

Injection Details

Injection Name:	**KS-110J-RAC**	*Run Time (min):*	**25.00**
Vial Number:	**GA4**	*Injection Volume:*	**20.00**
Injection Type:	**Unknown**	*Channel:*	**UV_VIS_1**
Calibration Level:		*Wavelength:*	**210.0**
Instrument Method:	**ASH 90 10 10 25**	*Bandwidth:*	**1**
Processing Method:	**EMP_aldolica**	*Dilution Factor:*	**1.0000**
Injection Date/Time:	**07/sep/18 22:45**	*Sample Weight:*	**1.0000**

Chromatogram

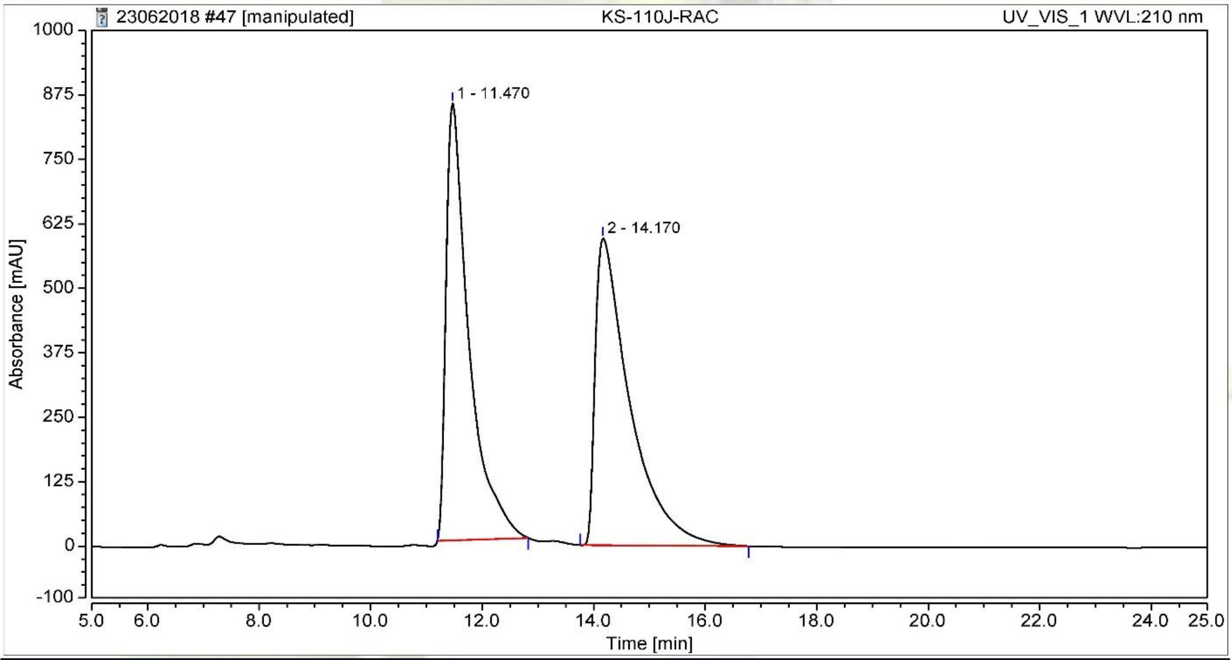

Integration Results

No.	Peak Name	Retention Time min	Area mAU*min	Height mAU	Relative Area %	Relative Height %	Amount n.a.
1		11.470	402.480	847.061	48.52	58.72	n.a.
2		14.170	426.960	595.547	51.48	41.28	n.a.
**Total:**			**829.440**	**1442.608**	**100.00**	**100.00**	

(*R*)-36. Main product in a mixture of enantiomers.

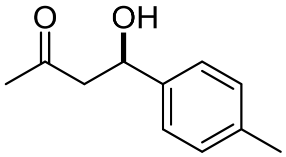

(*R*)-**36**

Chromatogram and Results

Injection Details			
Injection Name:	**KS-110J-EE**	*Run Time (min):*	**20.00**
Vial Number:	**GA7**	*Injection Volume:*	**20.00**
Injection Type:	**Unknown**	*Channel:*	**UV_VIS_1**
Calibration Level:		*Wavelength:*	**210.0**
Instrument Method:	**ASH 90 10 10 20**	*Bandwidth:*	**2**
Processing Method:	**EMP_aldolica**	*Dilution Factor:*	**1.0000**
Injection Date/Time:	**19/sep/18 13:19**	*Sample Weight:*	**1.0000**

Chromatogram

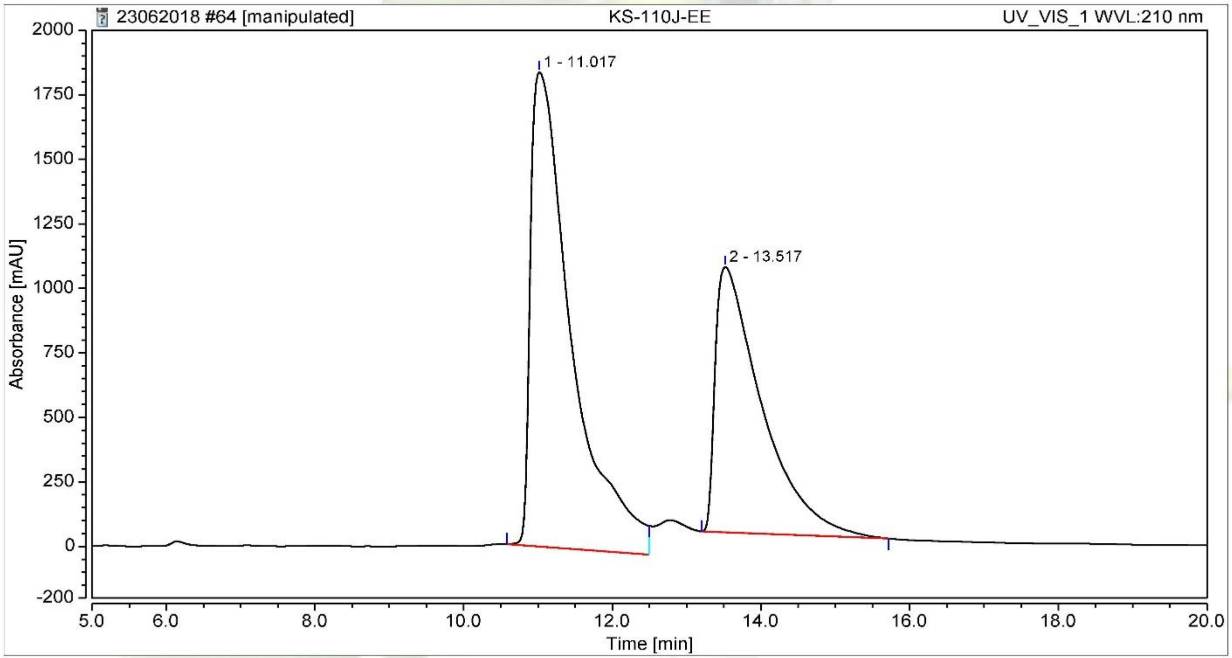

Integration Results

No.	Peak Name	Retention Time min	Area mAU*min	Height mAU	Relative Area %	Relative Height %	Amount n.a.
1		11.017	1168.378	1837.981	61.09	64.11	n.a.
2		13.517	744.192	1029.112	38.91	35.89	n.a.
**Total:**			**1912.570**	**2867.093**	**100.00**	**100.00**	

***rac*-37, reference sample.**

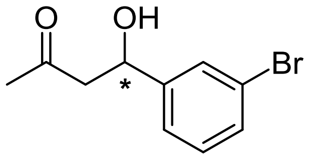

rac-**37**

Chromatogram and Results

Injection Details

Injection Name:	**KS-110M-RAC**	*Run Time (min):*	**35.00**
Vial Number:	**GA4**	*Injection Volume:*	**20.00**
Injection Type:	**Unknown**	*Channel:*	**UV_VIS_1**
Calibration Level:		*Wavelength:*	**210.0**
Instrument Method:	**ASH 95 05 08 35**	*Bandwidth:*	**2**
Processing Method:	**EMP_aldolica**	*Dilution Factor:*	**1.0000**
Injection Date/Time:	**08/sep/18 16:18**	*Sample Weight:*	**1.0000**

Chromatogram

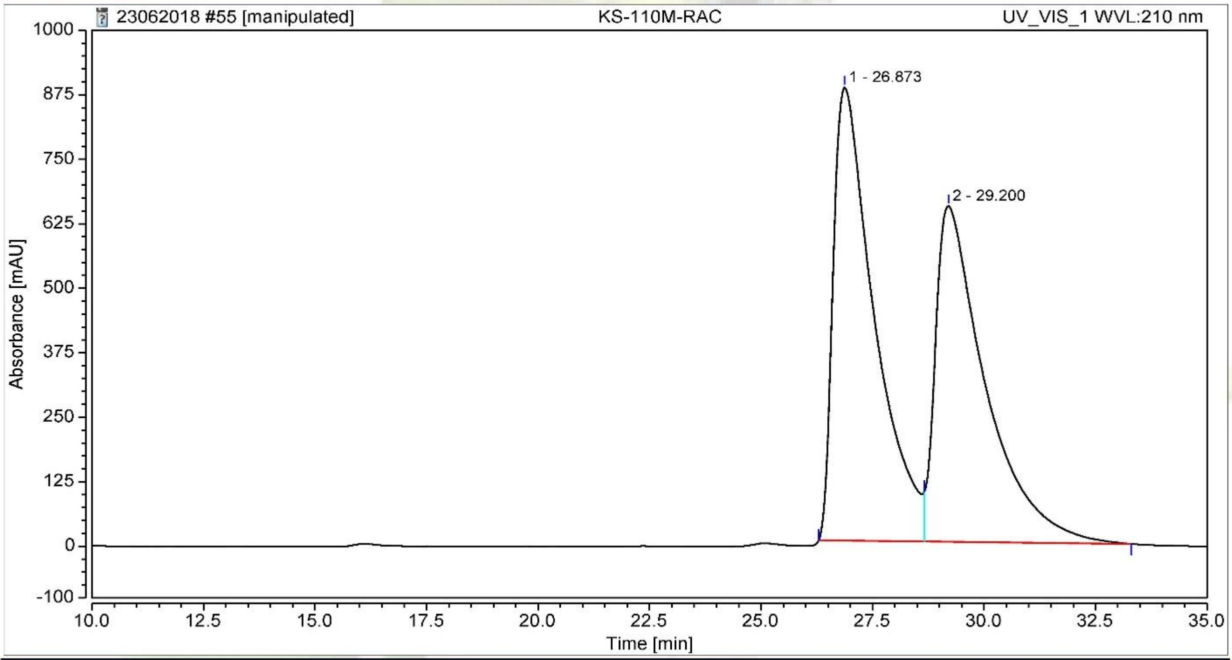

Integration Results

No.	Peak Name	Retention Time min	Area mAU*min	Height mAU	Relative Area %	Relative Height %	Amount n.a.
1		26.873	936.557	878.341	52.67	57.43	n.a.
2		29.200	841.734	650.951	47.33	42.57	n.a.
**Total:**			**1778.291**	**1529.292**	**100.00**	**100.00**	

(*R*)-37. Main product in a mixture of enantiomers.

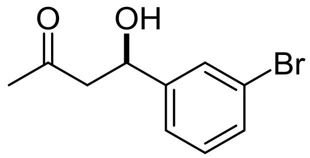

(*R*)-**37**

Chromatogram and Results

Injection Details

Injection Name:	**KS-110M-EE-00**	*Run Time (min):*	**35.00**
Vial Number:	**GB1**	*Injection Volume:*	**20.00**
Injection Type:	**Unknown**	*Channel:*	**UV_VIS_1**
Calibration Level:		*Wavelength:*	**210.0**
Instrument Method:	**ASH 95 05 08 35**	*Bandwidth:*	**2**
Processing Method:	**EMP_aldolica**	*Dilution Factor:*	**1.0000**
Injection Date/Time:	**29/sep/18 19:24**	*Sample Weight:*	**1.0000**

Chromatogram

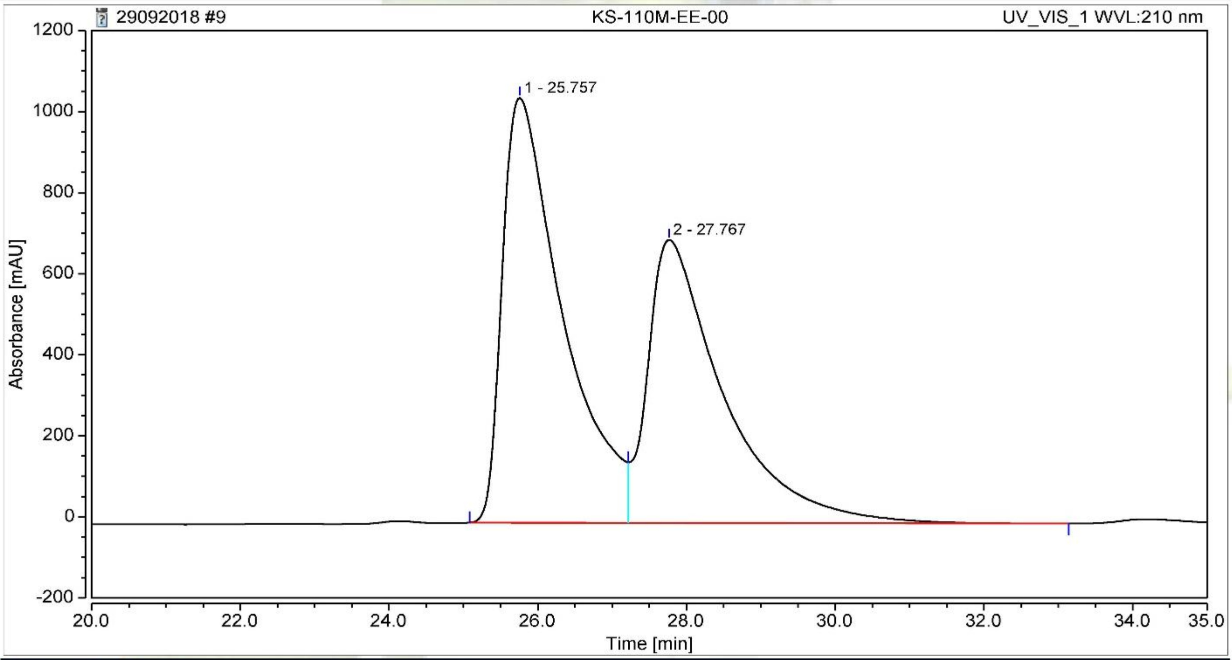

Integration Results

No.	Peak Name	Retention Time min	Area mAU*min	Height mAU	Relative Area %	Relative Height %	Amount n.a.
1		25.757	982.979	1047.751	54.56	60.00	n.a.
2		27.767	818.730	698.545	45.44	40.00	n.a.
**Total:**			**1801.709**	**1746.296**	**100.00**	**100.00**	

c) Aldol products with isatins.

(*R*,*S*)-38. **Main product in a mixture of 4 diastereomers.**

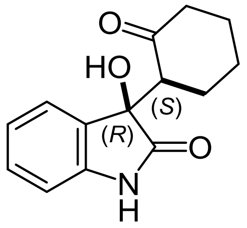

(*R*,*S*)–**38**

Chromatogram and Results

Injection Details

Injection Name:	**KS-125A-EE**	*Run Time (min):*	**100.00**
Vial Number:	**BA5**	*Injection Volume:*	**20.00**
Injection Type:	**Unknown**	*Channel:*	**UV_VIS_1**
Calibration Level:		*Wavelength:*	**210.0**
Instrument Method:	**ADH 90 10 10 100**	*Bandwidth:*	**2**
Processing Method:	**EMP_aldolica**	*Dilution Factor:*	**1.0000**
Injection Date/Time:	**27/oct/18 03:06**	*Sample Weight:*	**1.0000**

Chromatogram

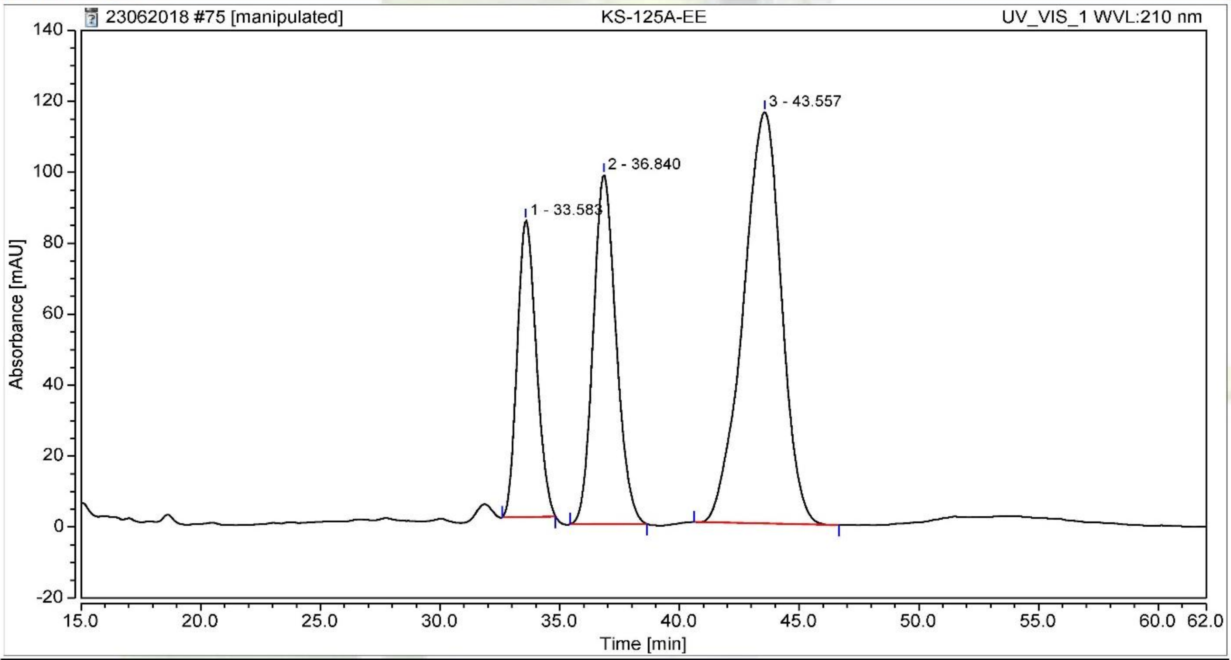

Integration Results

No.	Peak Name	Retention Time min	Area mAU*min	Height mAU	Relative Area %	Relative Height %	Amount n.a.
1		33.583	76.869	83.652	19.20	28.05	n.a.
2		36.840	109.308	98.523	27.30	33.04	n.a.
3		43.557	214.287	116.060	53.51	38.92	n.a.
**Total:**			**400.465**	**298.235**	**100.00**	**100.00**	

***Rac*-39. Reference sample.**

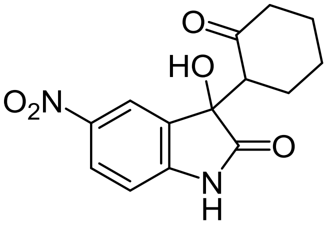

rac–**39**

Chromatogram and Results

Injection Details

Injection Name:	**KS-119A**	*Run Time (min):*	**60.00**
Vial Number:	**RA4**	*Injection Volume:*	**20.00**
Injection Type:	**Unknown**	*Channel:*	**UV_VIS_4**
Calibration Level:		*Wavelength:*	**210.0**
Instrument Method:	**AD-H 8020 05 60**	*Bandwidth:*	**2**
Processing Method:	**EMP_aldolica**	*Dilution Factor:*	**1.0000**
Injection Date/Time:	**26/jul/18 15:50**	*Sample Weight:*	**1.0000**

Chromatogram

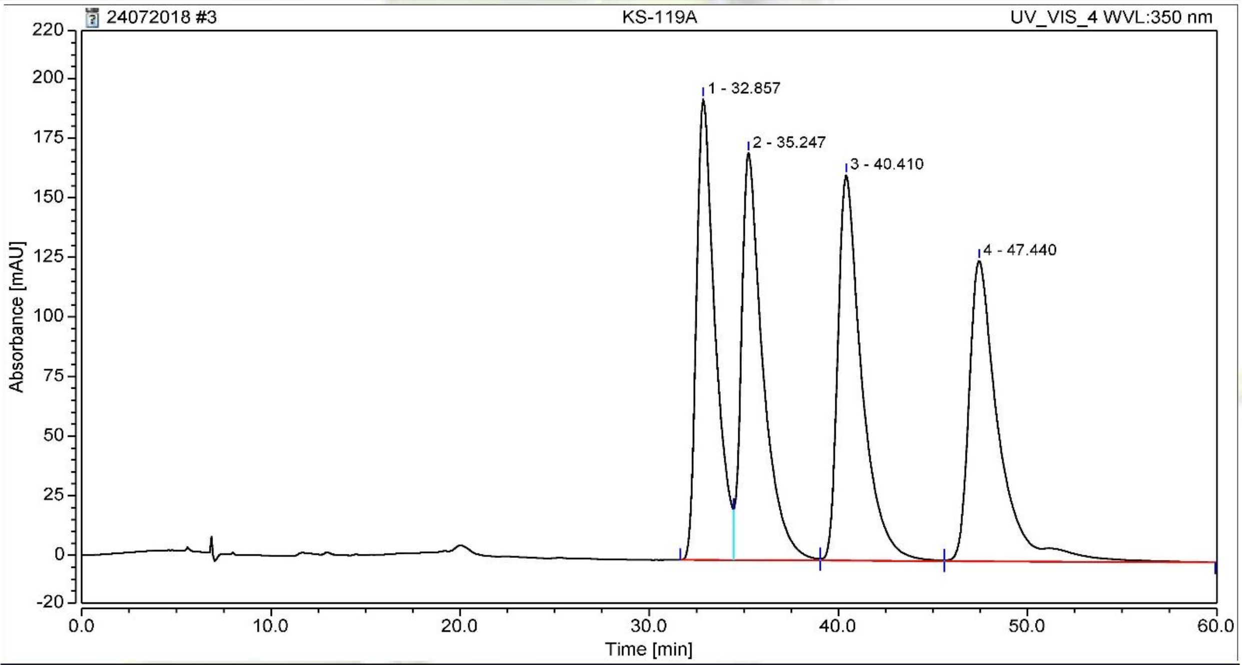

Integration Results

No.	Peak Name	Retention Time min	Area mAU*min	Height mAU	Relative Area %	Relative Height %	Amount n.a.
1		32.857	221.526	193.384	24.28	29.65	n.a.
2		35.247	225.118	170.806	24.67	26.19	n.a.
3		40.410	236.386	161.894	25.91	24.82	n.a.
4		47.440	229.412	126.178	25.14	19.34	n.a.
**Total:**			**912.442**	**652.261**	**100.00**	**100.00**	

(*R*,*S*)-39. Main product in a mixture of 4 diastereomers.

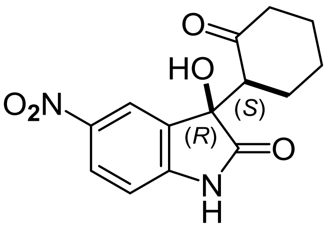

(*R*,*S*)–**39**

Chromatogram and Results

Injection Details

Injection Name:	**KS- 115 B**	*Run Time (min):*	**70.00**
Vial Number:	**RA2**	*Injection Volume:*	**20.00**
Injection Type:	**Unknown**	*Channel:*	**UV_VIS_3**
Calibration Level:		*Wavelength:*	**210.0**
Instrument Method:	**AD-H 8020 05 60**	*Bandwidth:*	**1**
Processing Method:	**EMP_aldolica**	*Dilution Factor:*	**1.0000**
Injection Date/Time:	**24/jul/18 18:01**	*Sample Weight:*	**1.0000**

Chromatogram

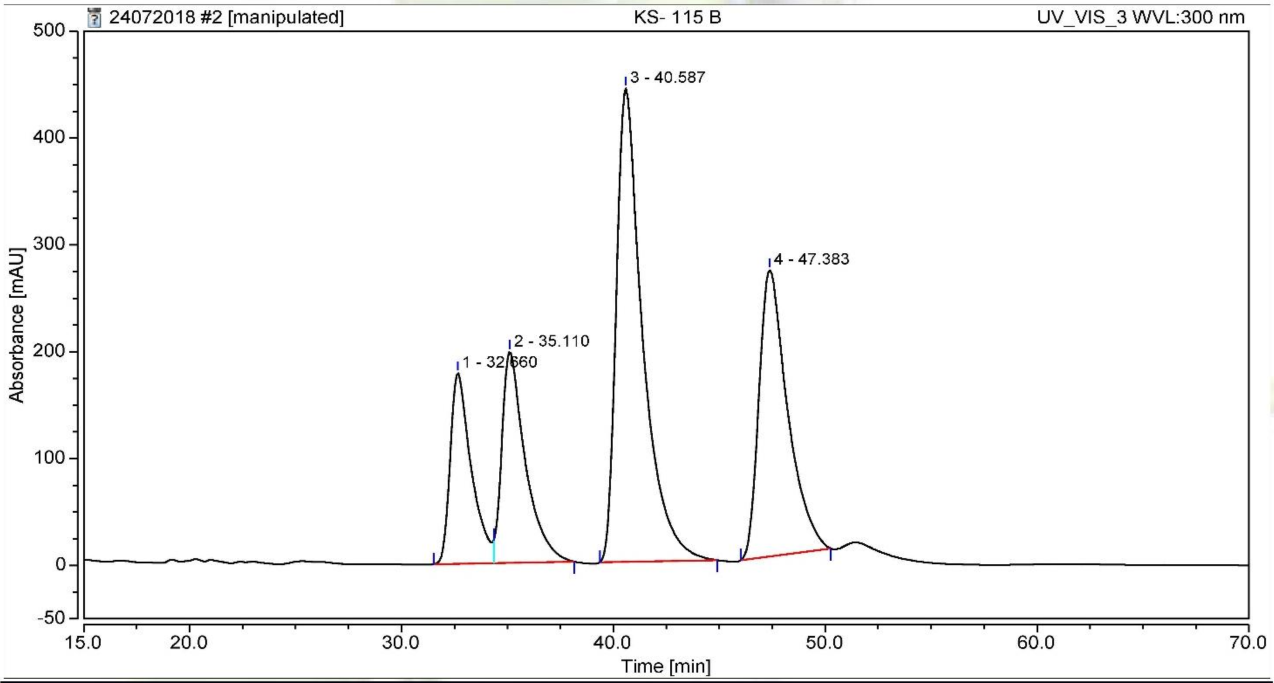

Integration Results

No.	Peak Name	Retention Time min	Area mAU*min	Height mAU	Relative Area %	Relative Height %	Amount n.a.
1		32.660	205.791	178.701	13.55	16.43	n.a.
2		35.110	253.231	197.666	16.67	18.17	n.a.
3		40.587	640.825	443.293	42.18	40.75	n.a.
4		47.383	419.244	268.080	27.60	24.65	n.a.
**Total:**			**1519.092**	**1087.739**	**100.00**	**100.00**	

(*R,S*)-40. Main product in a mixture of 4 diastereomers.

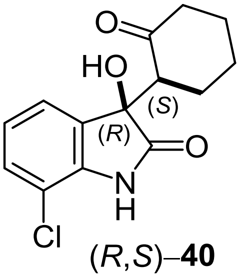

(*R*,*S*)–**40**

Chromatogram and Results

Injection Details

Injection Name:	**KS-125C-EE**	*Run Time (min):*	**115.00**
Vial Number:	**BA4**	*Injection Volume:*	**20.00**
Injection Type:	**Unknown**	*Channel:*	**UV_VIS_1**
Calibration Level:		*Wavelength:*	**210.0**
Instrument Method:	**ADH 93 07 10 100**	*Bandwidth:*	**2**
Processing Method:	**EMP_aldolica**	*Dilution Factor:*	**1.0000**
Injection Date/Time:	**27/oct/18 01:04**	*Sample Weight:*	**1.0000**

Chromatogram

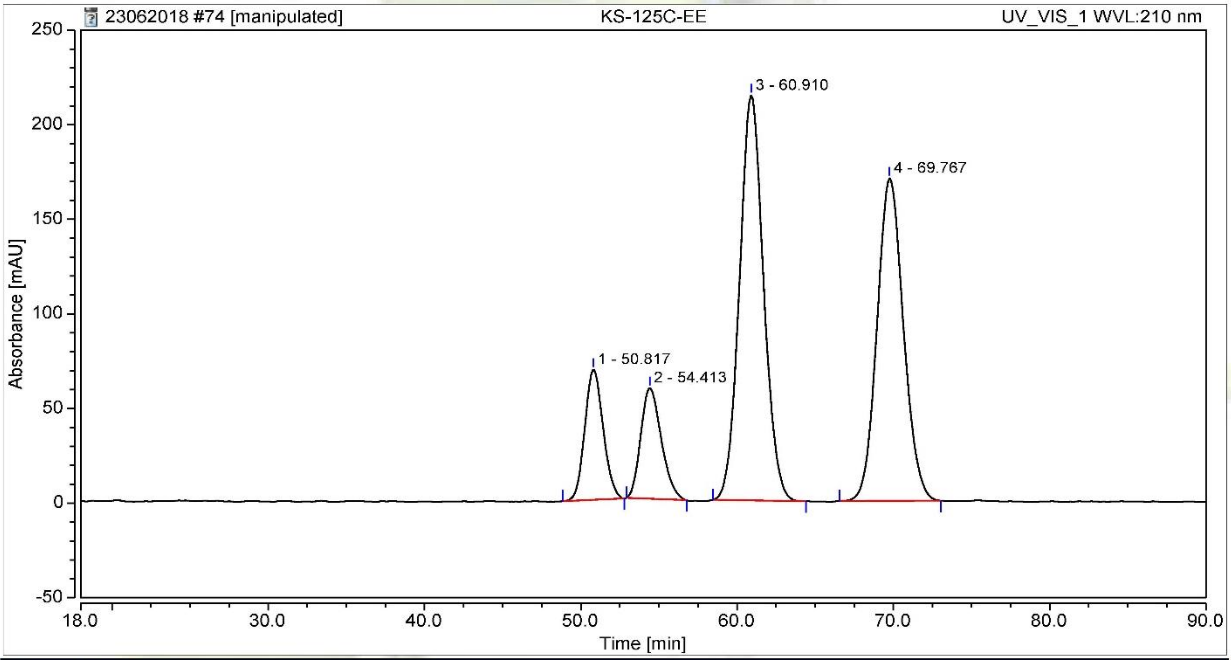

Integration Results

No.	Peak Name	Retention Time min	Area mAU*min	Height mAU	Relative Area %	Relative Height %	Amount n.a.
1		50.817	93.859	68.905	10.45	13.45	n.a.
2		54.413	89.258	58.643	9.94	11.45	n.a.
3		60.910	381.058	214.105	42.44	41.80	n.a.
4		69.767	333.705	170.501	37.17	33.29	n.a.
**Total:**			**897.880**	**512.154**	**100.00**	**100.00**	

2. NMR Spectra for previous characterized compounds and aldol products.

^{1}H and ^{13}C NMR of (*S*)-**3.**

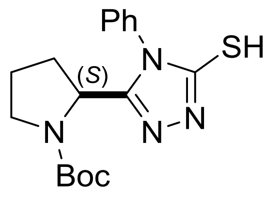

(*S*)**-3**

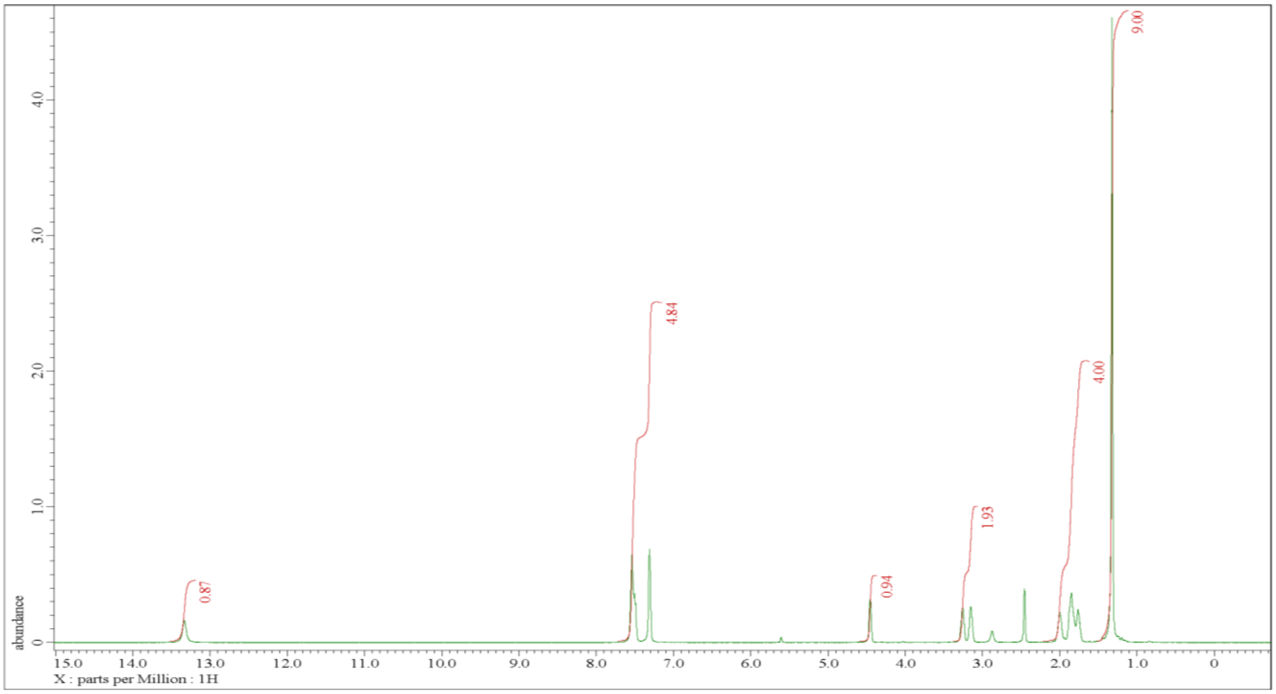

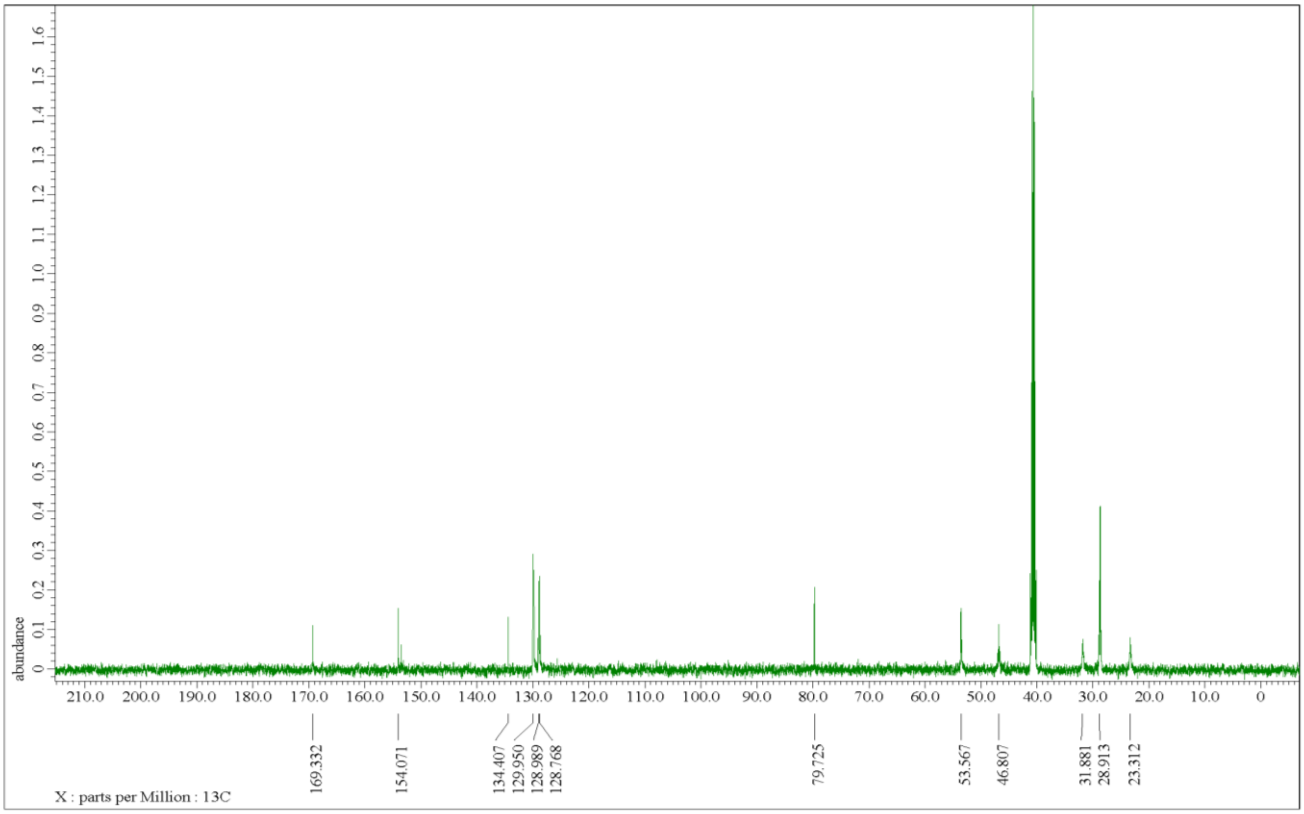

^{1}H and ^{13}C NMR of (*S*)-**10**.

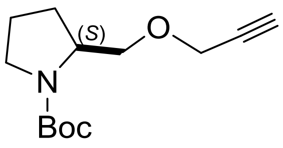

(*S*)-**10**

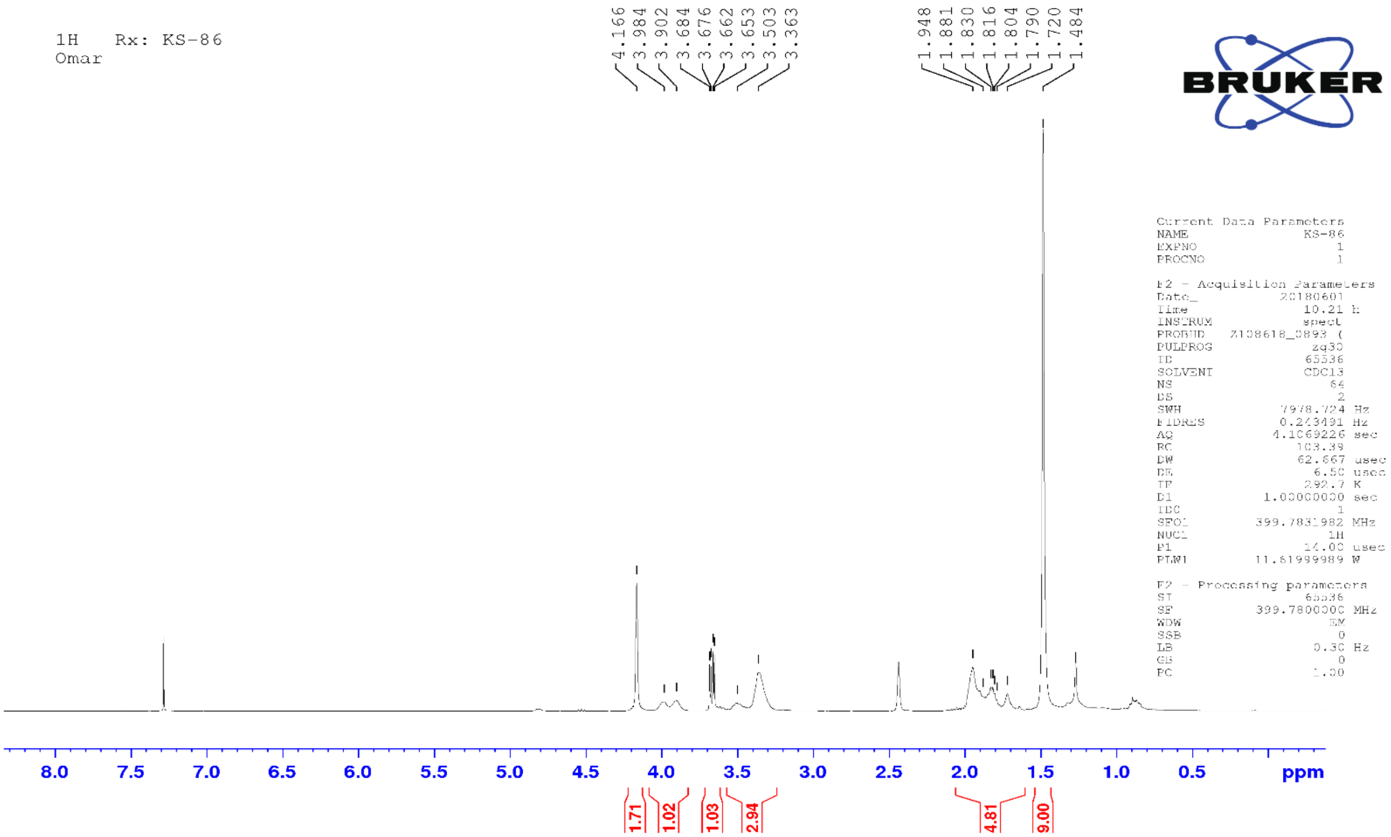

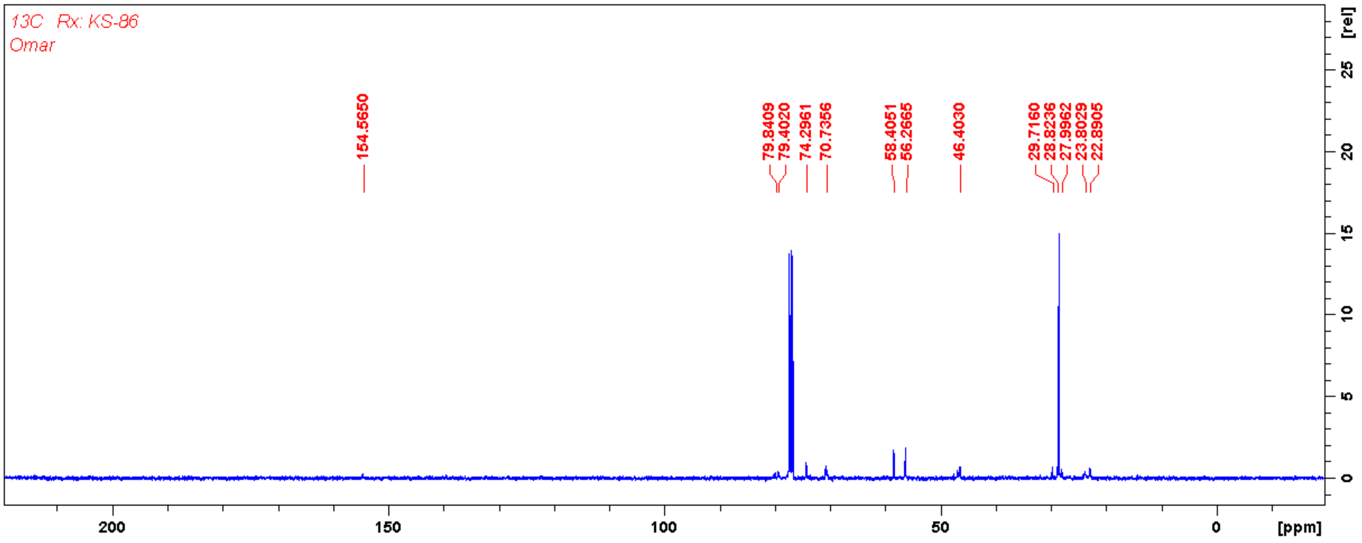

^{1}H and ^{13}C NMR of (2*S*,4*R*,*1´S*)-**15**.

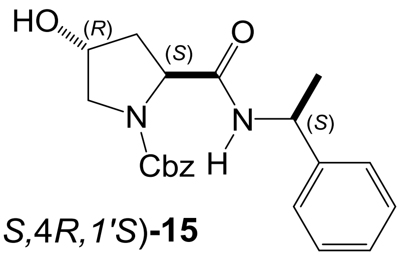

(2*S*,4*R*,*1'S*)-**15**

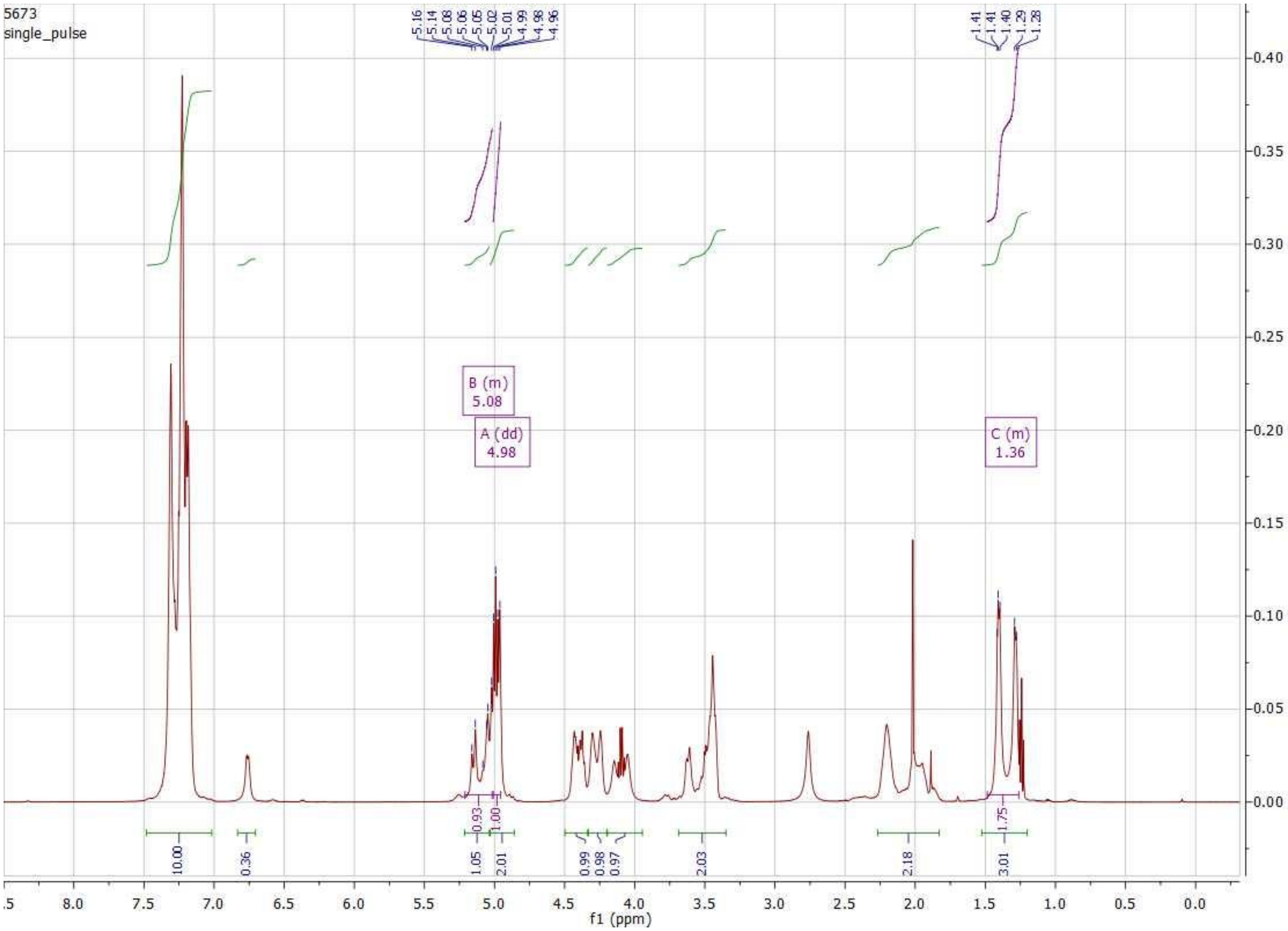

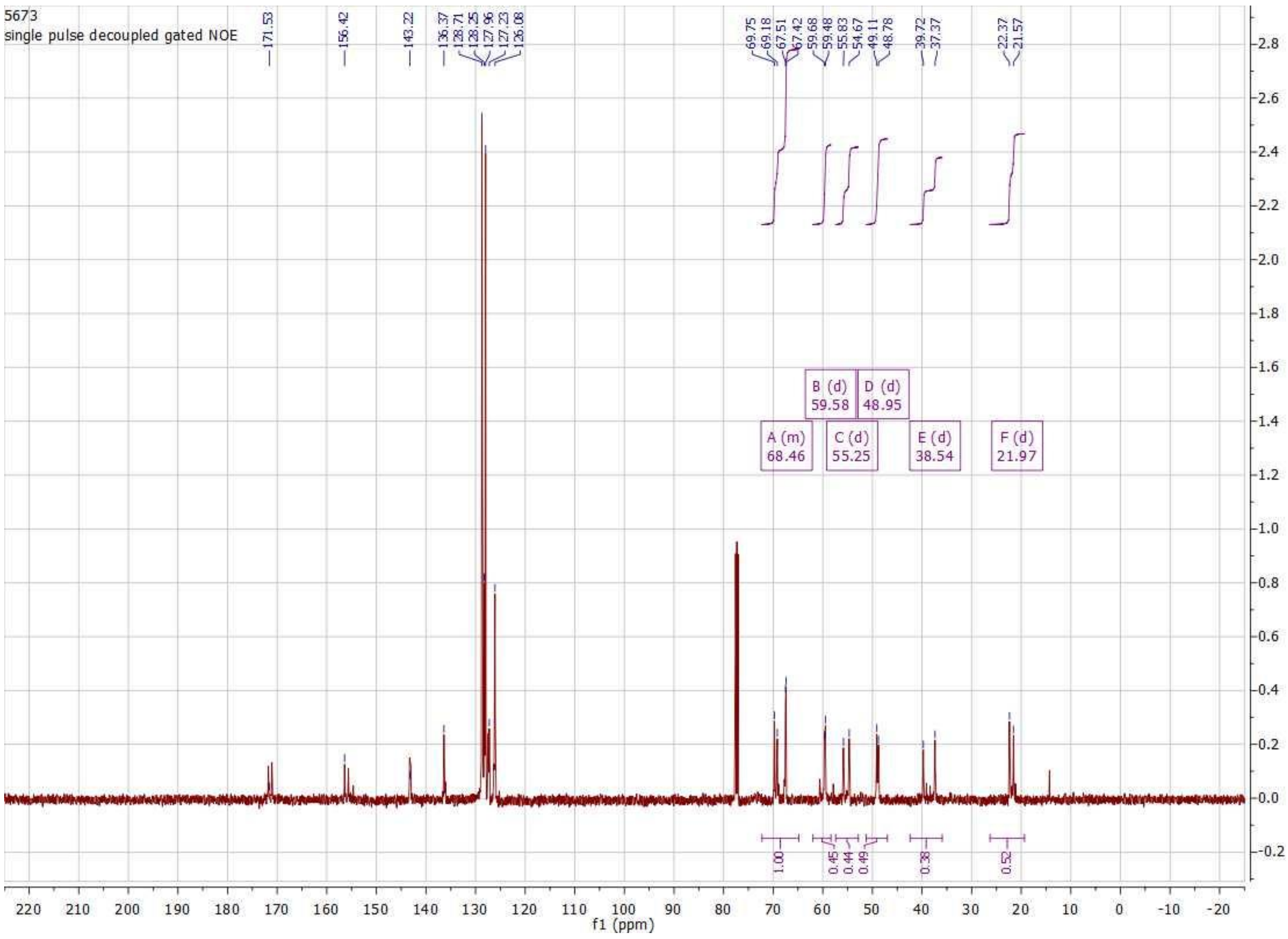

5673
single pulse decoupled gated NOE
171.53
156.42
143.22
136.37
128.71
128.25
127.96
127.23
126.08
69.75
69.18
67.51
67.42
59.68
59.48
55.83
54.67
49.11
48.78
39.72
37.37
22.37
21.57
B (d) 59.58
D (d) 48.95
A (m) 68.46
C (d) 55.25
E (d) 38.54
F (d) 21.97
1.00
0.45
0.44
0.49
0.38
0.52
f1 (ppm)

^{1}H and 13C NMR of (2*S*,4*R*,1´*R*)-**15**.

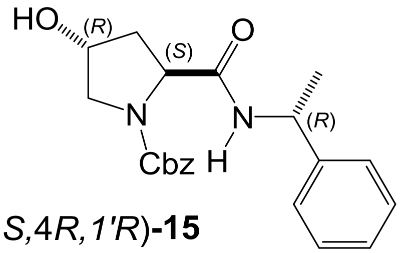

(2*S*,4*R*,*1'R*)**-15**

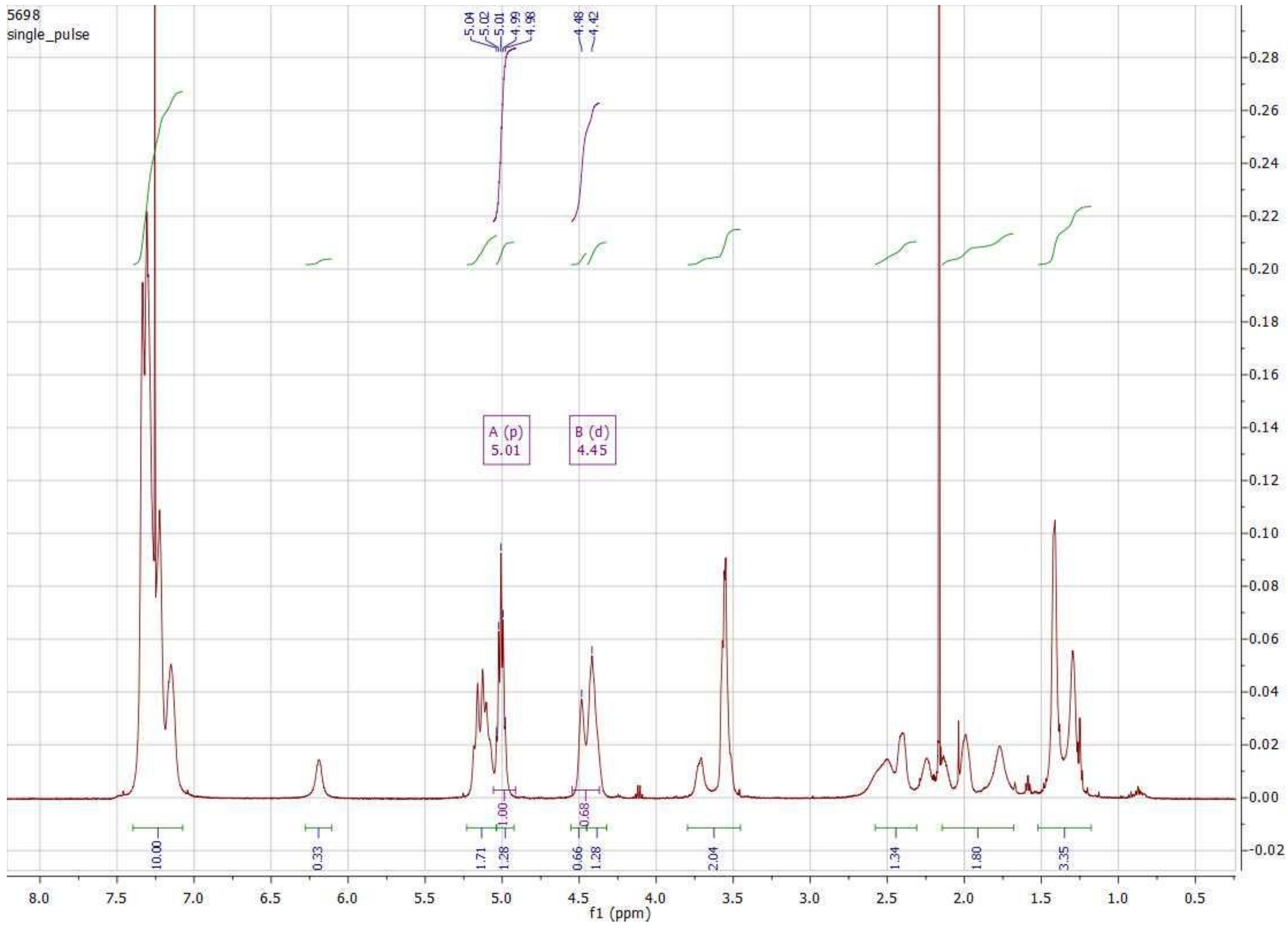

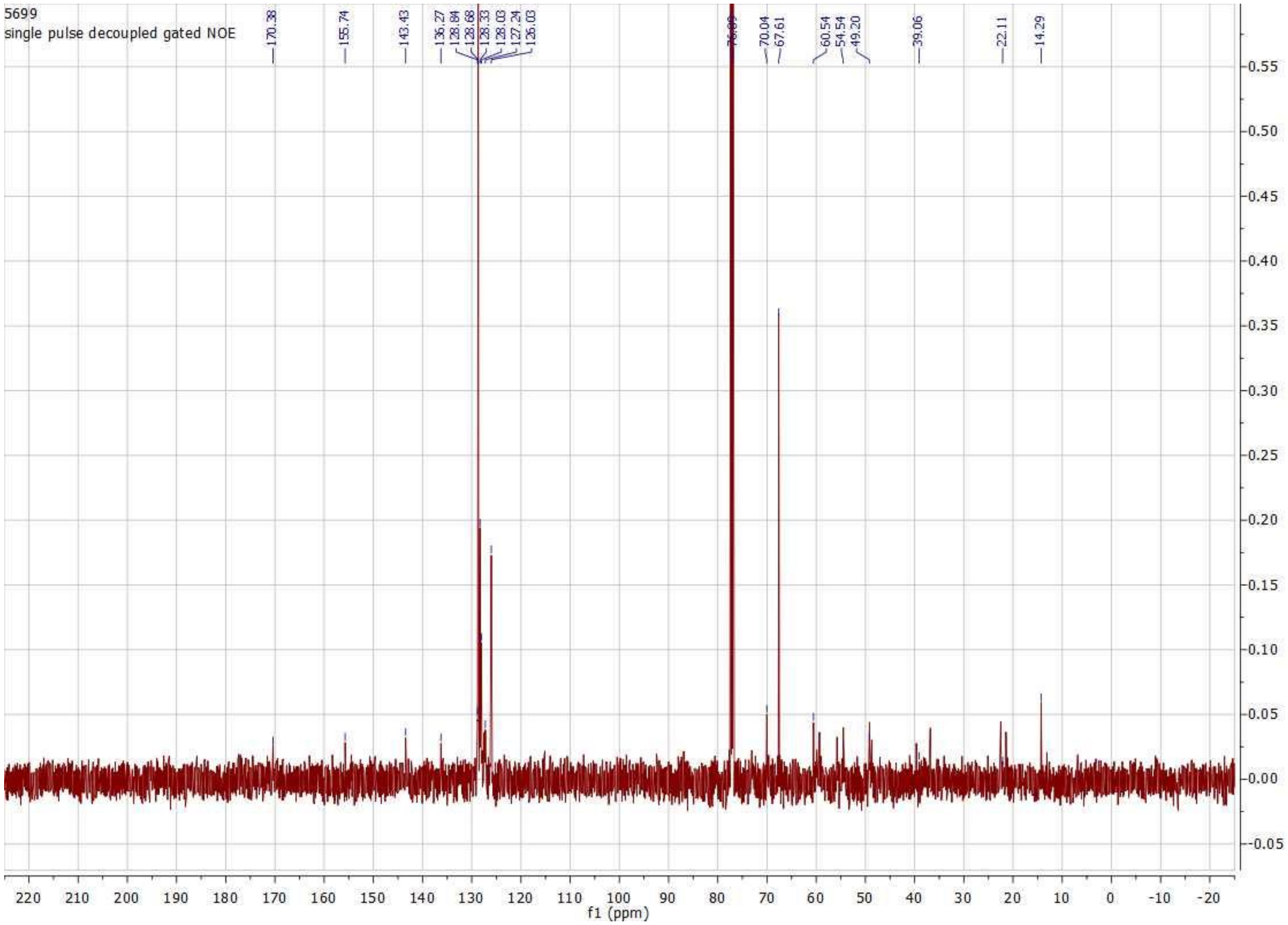
5699
single pulse decoupled gated NOE
170.38
155.74
143.43
136.27
128.84
128.68
128.33
128.03
127.24
126.03
70.04
67.61
60.54
54.54
49.20
39.06
22.11
14.29
f1 (ppm)

^{1}H and ^{13}C NMR of (2*S*,4*R*,*1´S*)-**16**, ^{1}H.

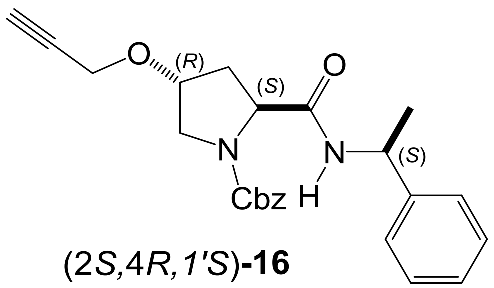

(2*S*,4*R*,*1'S*)-**16**

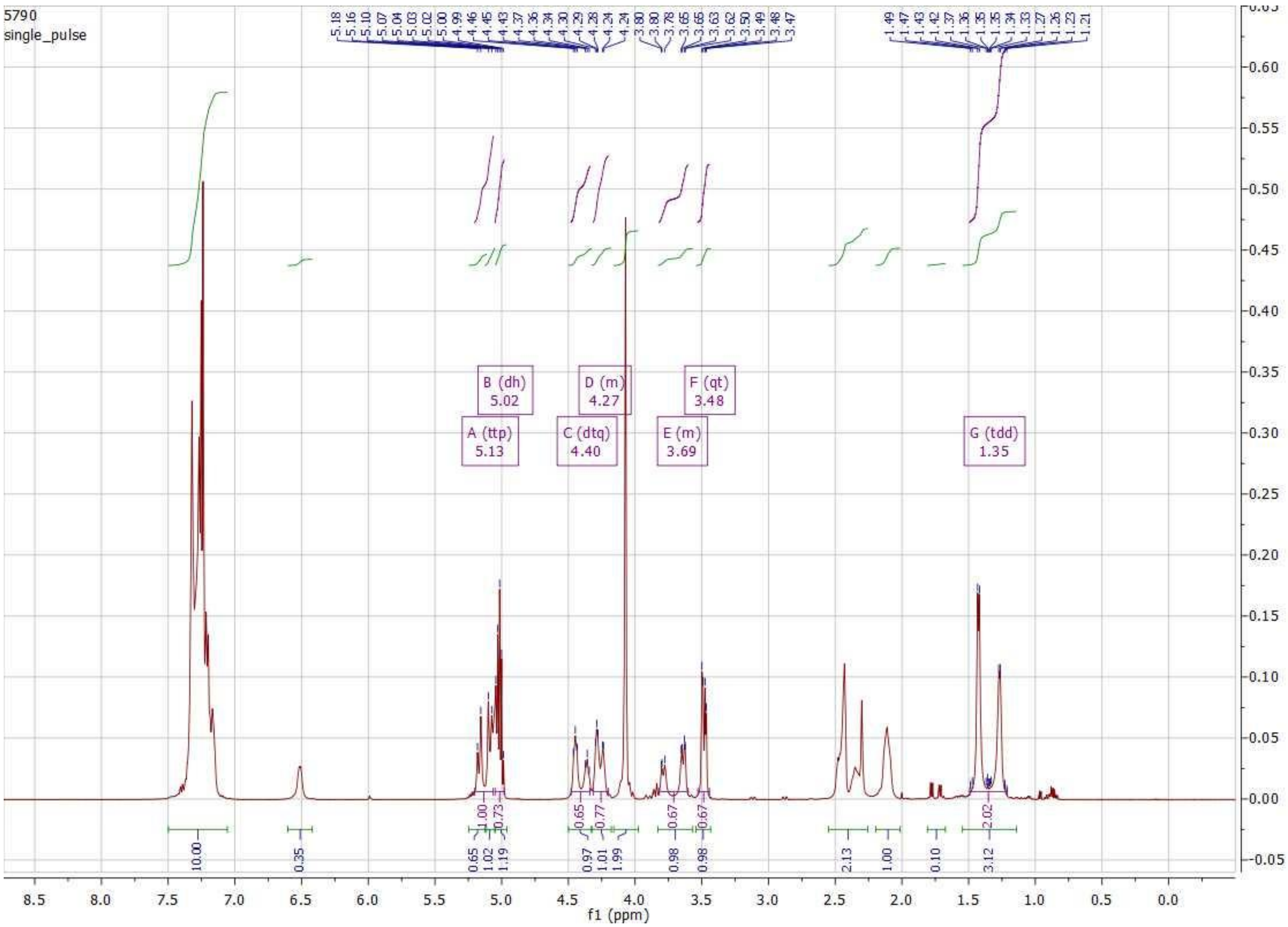

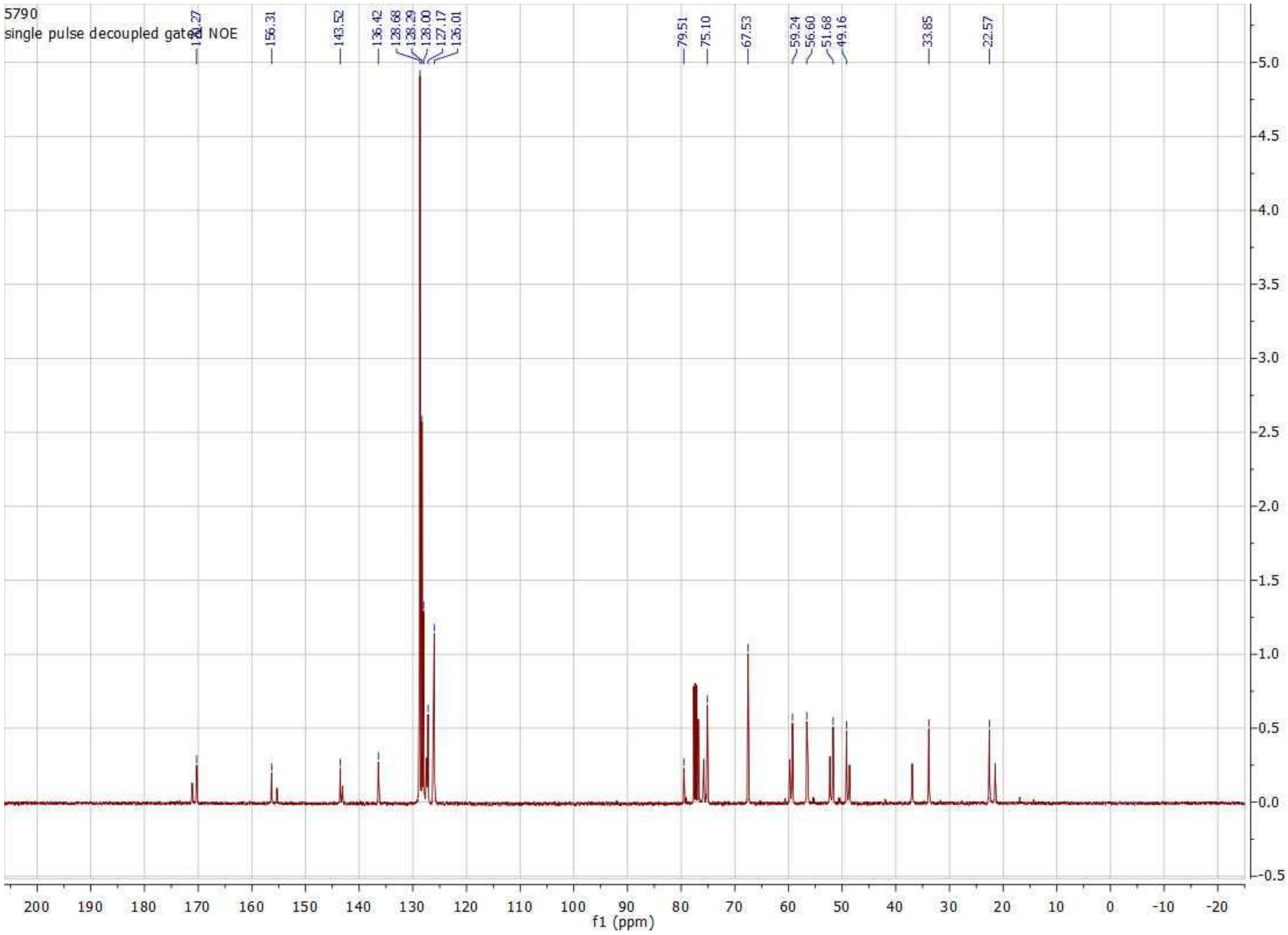

5790
single pulse decoupled gated NOE
156.31
143.52
136.42
128.68
128.29
128.00
127.17
126.01
79.51
75.10
67.53
59.24
56.60
51.68
49.16
33.85
22.57
f1 (ppm)

^{1}H and ^{13}C NMR of (2*S*,4*R*,*1´R*)-**16**.

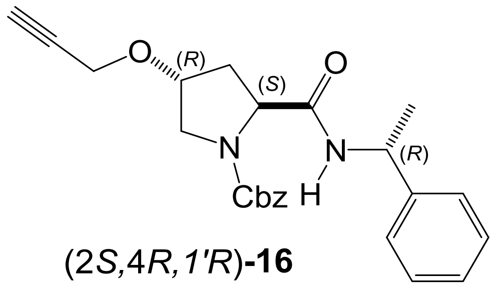

(2*S*,4*R*,*1'R*)-**16**

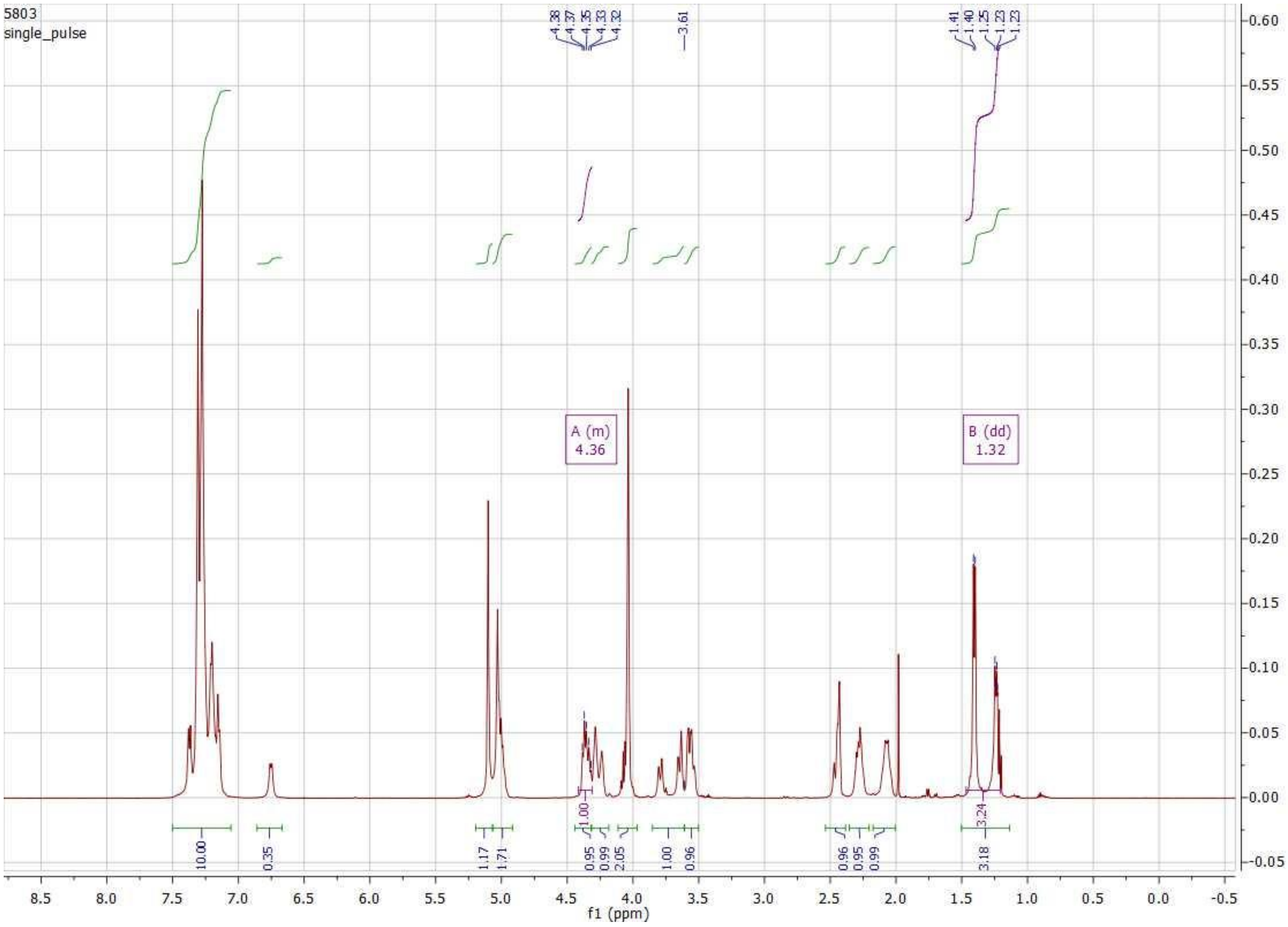

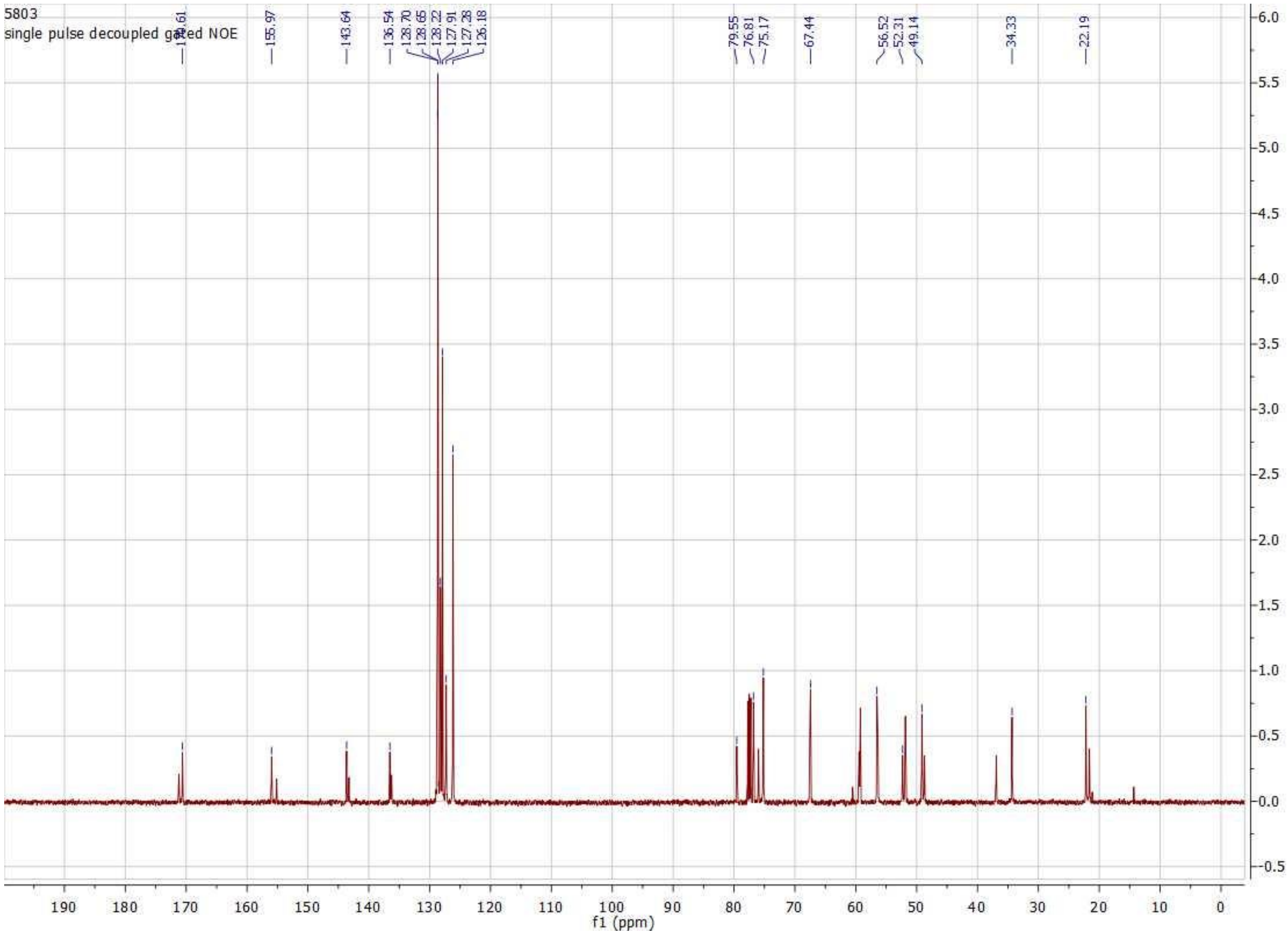

5803
single pulse decoupled gated NOE
170.61
155.97
143.64
136.54
128.70
128.65
128.22
127.91
127.28
126.18
79.55
76.81
75.17
67.44
56.52
52.31
49.14
34.33
22.19
f1 (ppm)

NMR of Aldol products containing (2*S*,1´*R*)**-19 to** (2*S*,1´*R*)**-26 as major product. In all cases, the spectra contain both *syn* and *anti* diastereomers.**

(2*S*,1´*R*)**-19 obtained with benzoic acid as additive**.

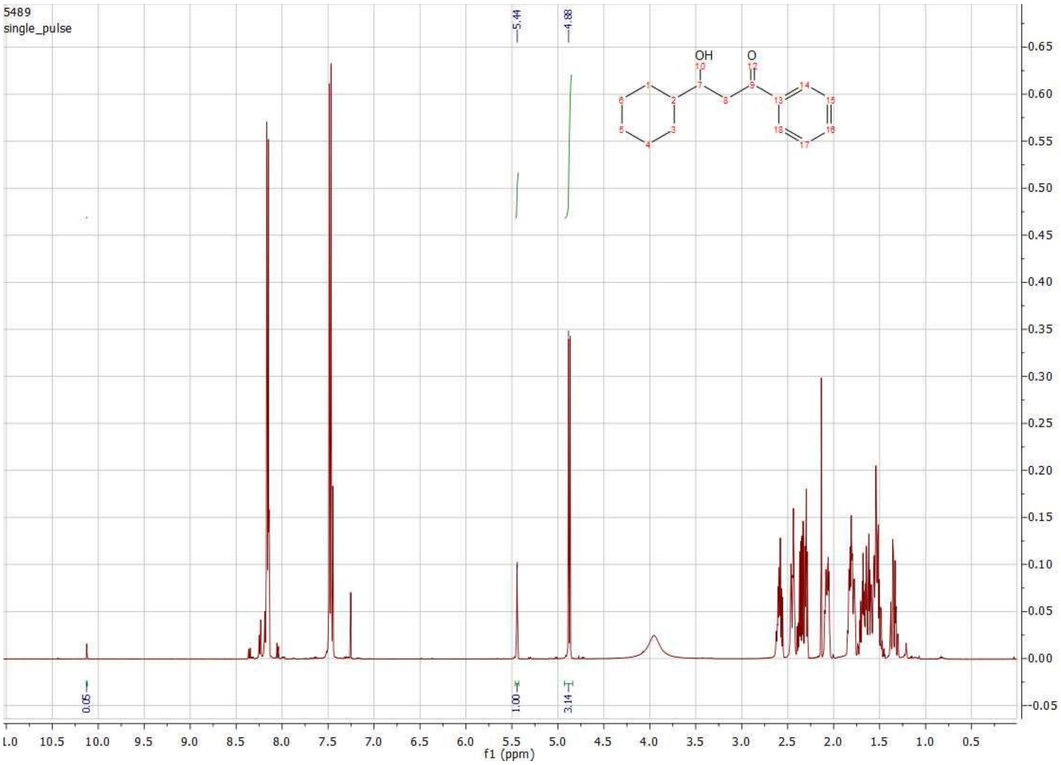

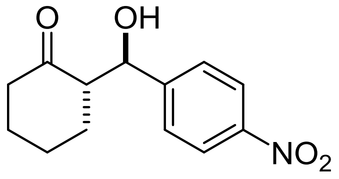

(2*S*,1´*R*)-**19**

(2*S*,1´*R*)**-19 obtained with salicylic acid as additive.**

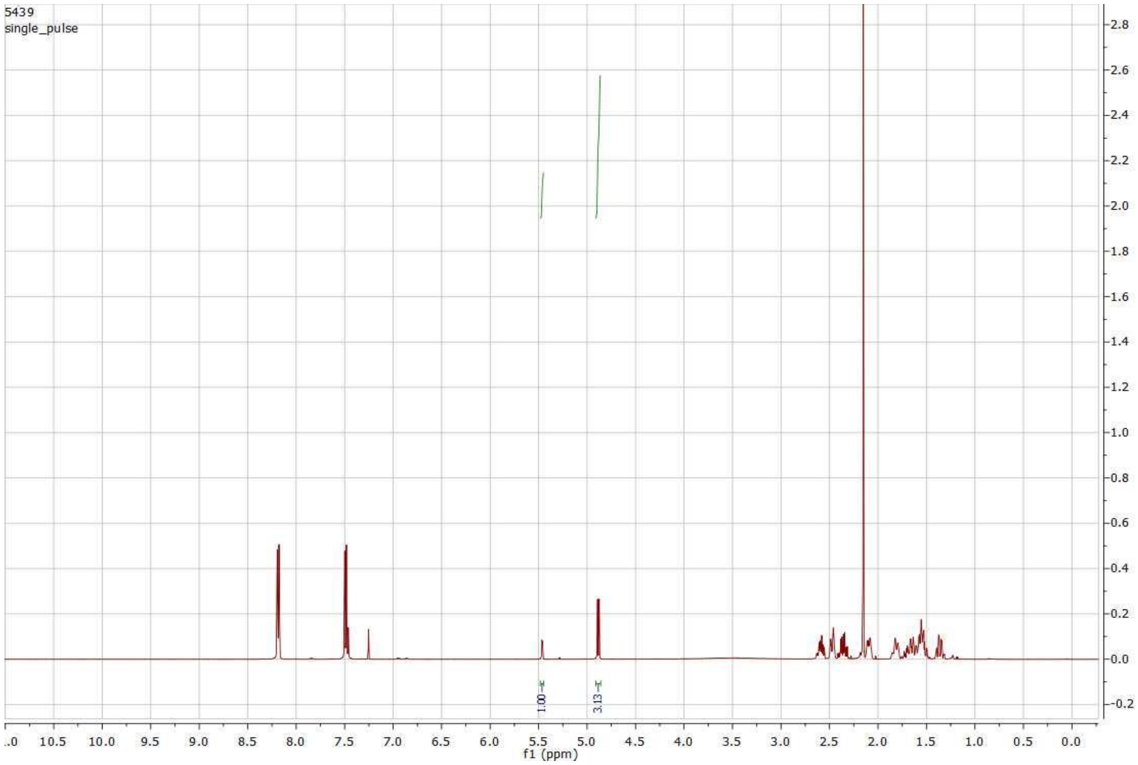

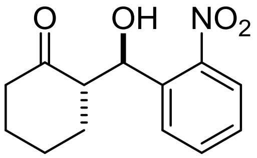

(2*S*,1´*R*)-**20**

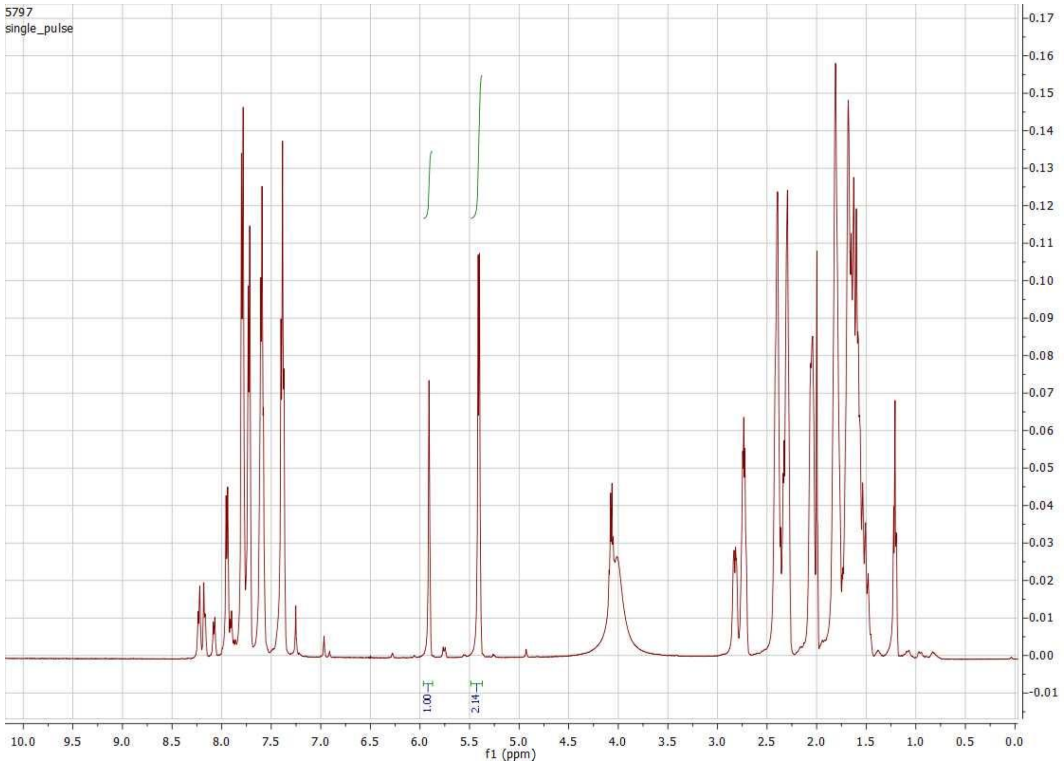

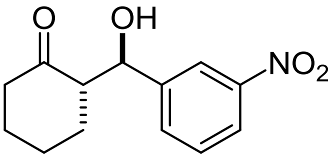

(2*S*,1´*R*)-**21**

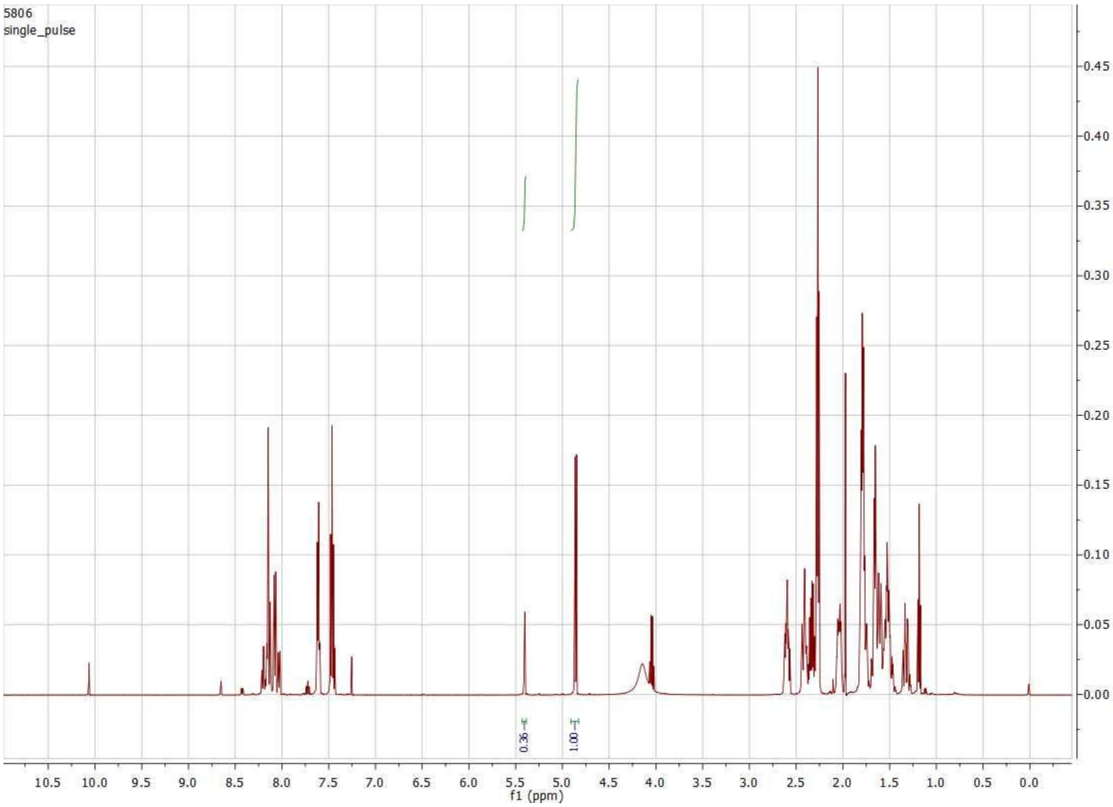

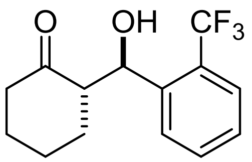

(2*S*,1´*R*)-**22**

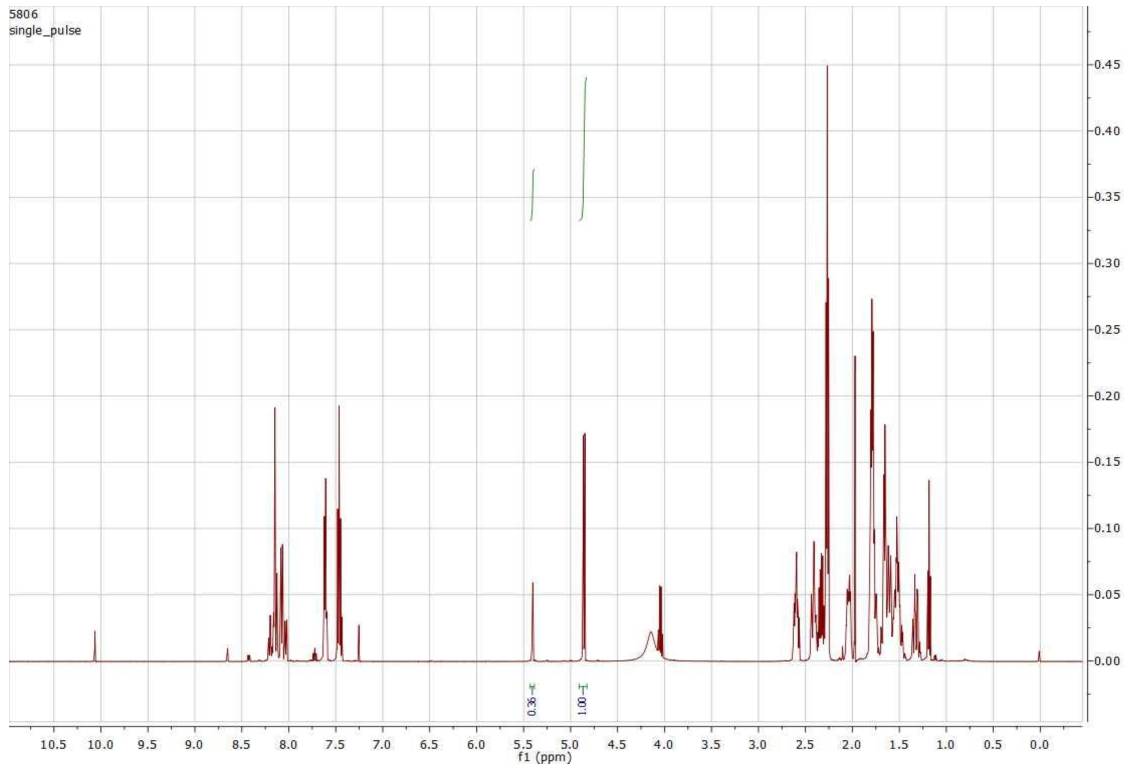

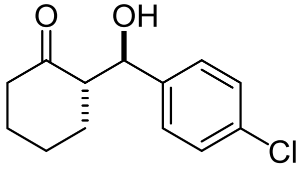
O
OH
Cl

(2*S*,1´*R*)-**23**

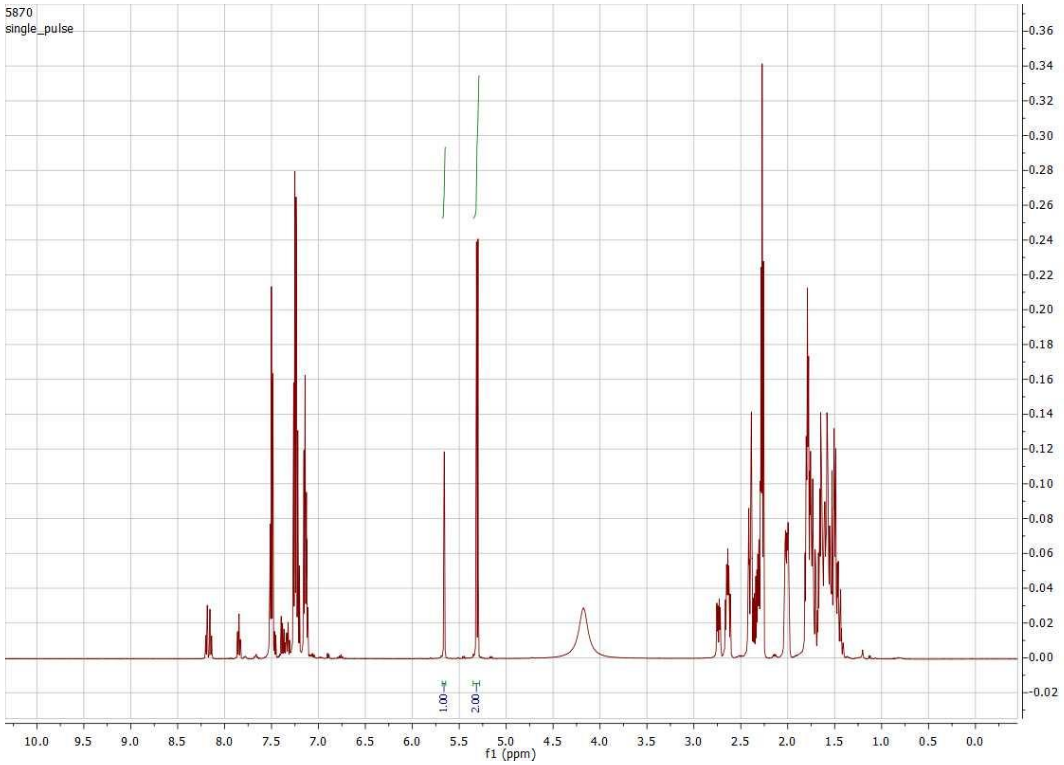
5870
single_pulse
1.00
2.00
10.0 9.5 9.0 8.5 8.0 7.5 7.0 6.5 6.0 5.5 5.0 4.5 4.0 3.5 3.0 2.5 2.0 1.5 1.0 0.5 0.0
f1 (ppm)
0.36
0.34
0.32
0.30
0.28
0.26
0.24
0.22
0.20
0.18
0.16
0.14
0.12
0.10
0.08
0.06
0.04
0.02
0.00
-0.02

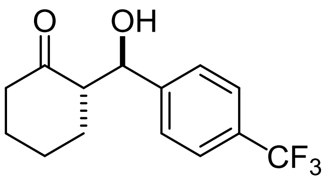

(2*S*,1´*R*)-**24**

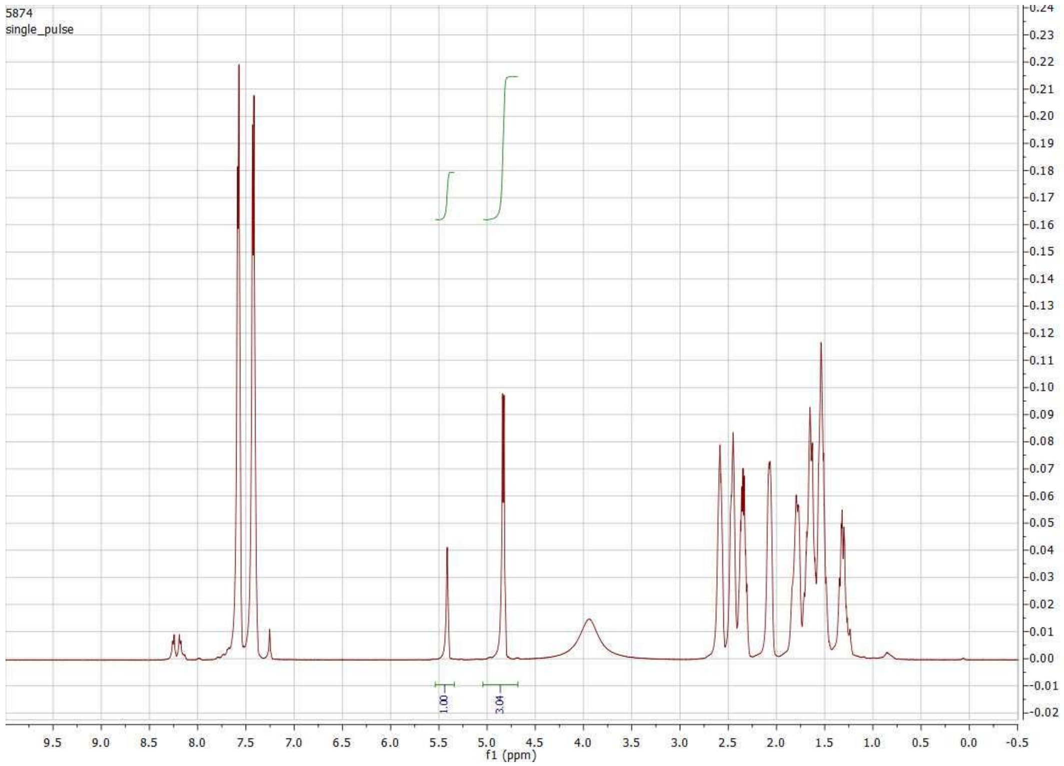

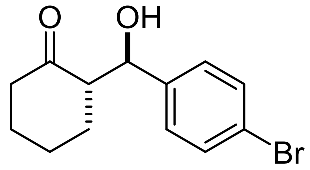

(2*S*,1´*R*)-**25**

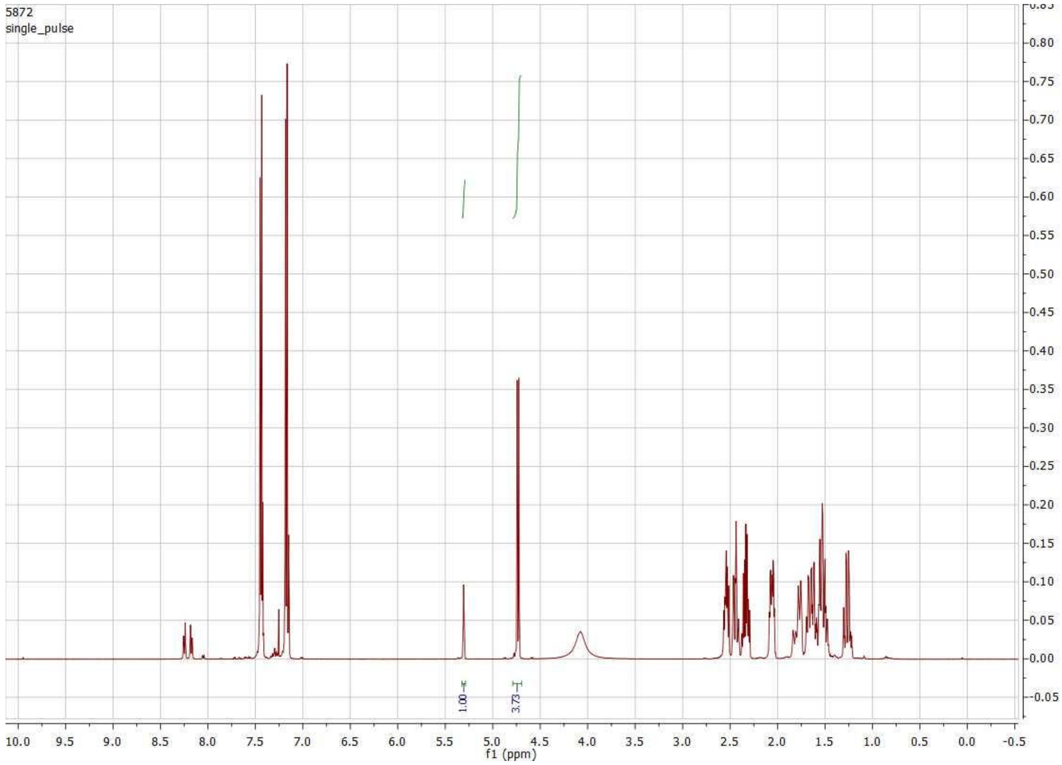

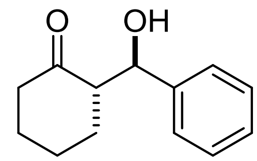

(2*S*,1´*R*)-**26**

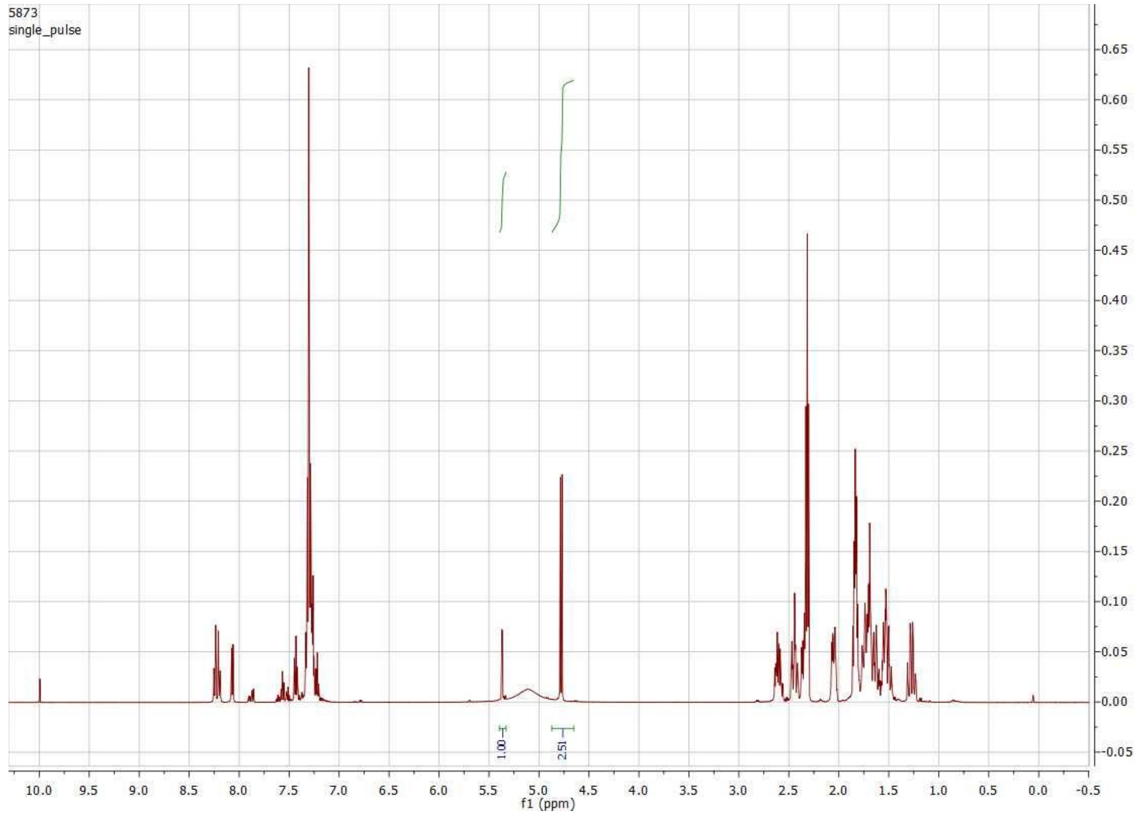

5873
single_pulse
1.00
2.51
10.0 9.5 9.0 8.5 8.0 7.5 7.0 6.5 6.0 5.5 5.0 4.5 4.0 3.5 3.0 2.5 2.0 1.5 1.0 0.5 0.0 -0.5
f1 (ppm)
0.65 0.60 0.55 0.50 0.45 0.40 0.35 0.30 0.25 0.20 0.15 0.10 0.05 0.00 -0.05

Aldol products obtained with the enamine derived from acetone:

^{1}H and ^{13}C NMR of (*R*)**-27**

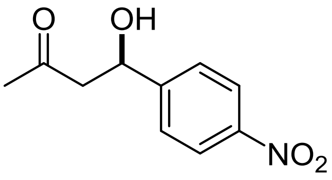

(*R*)-**27**

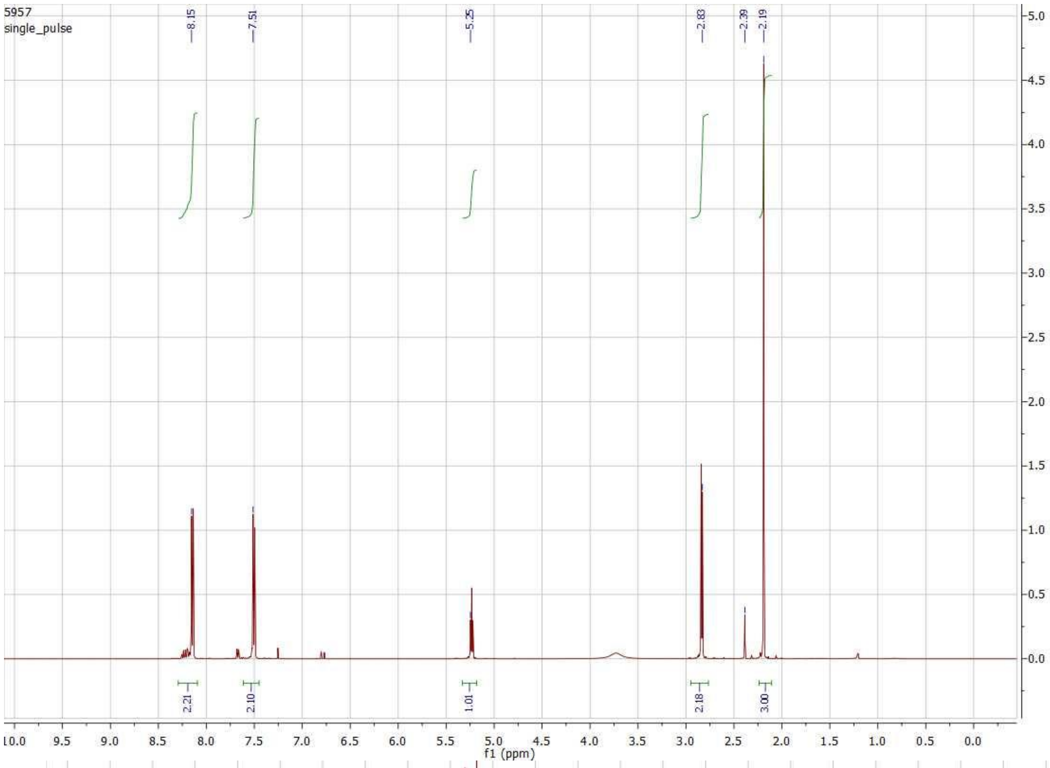

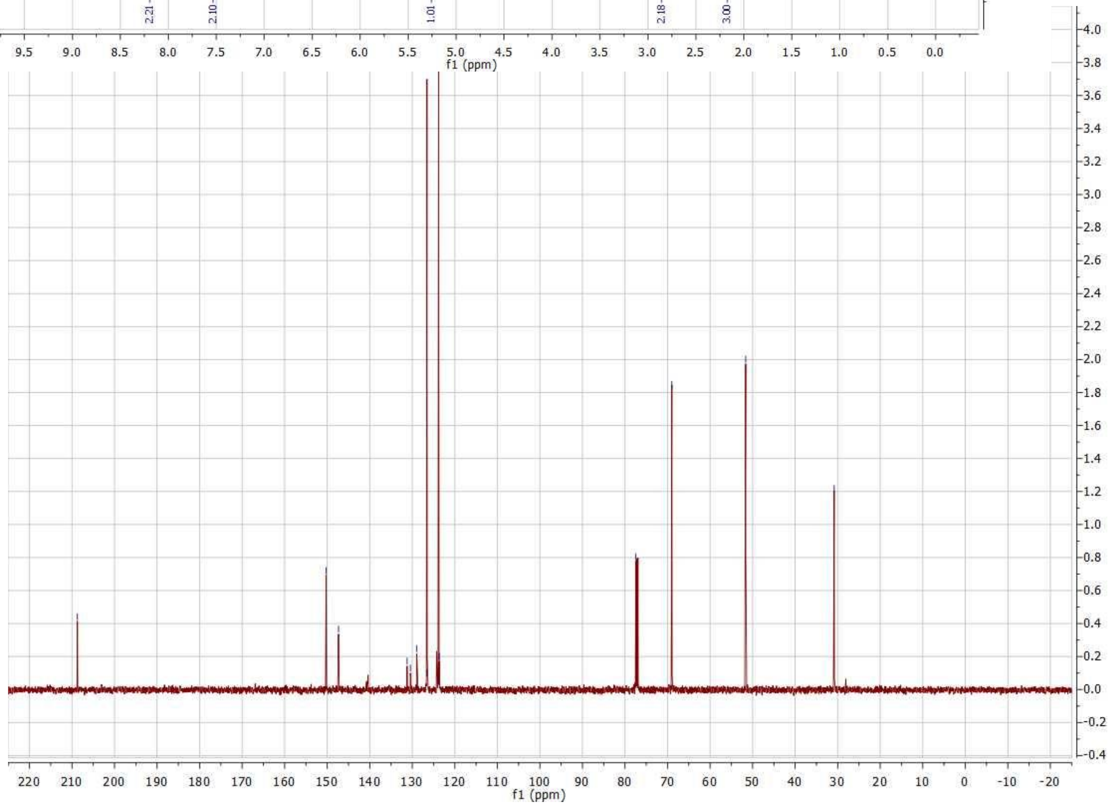

^{1}H and ^{13}C NMR of (*R*)-**28**

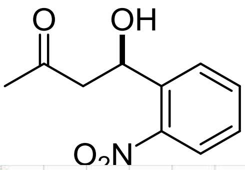

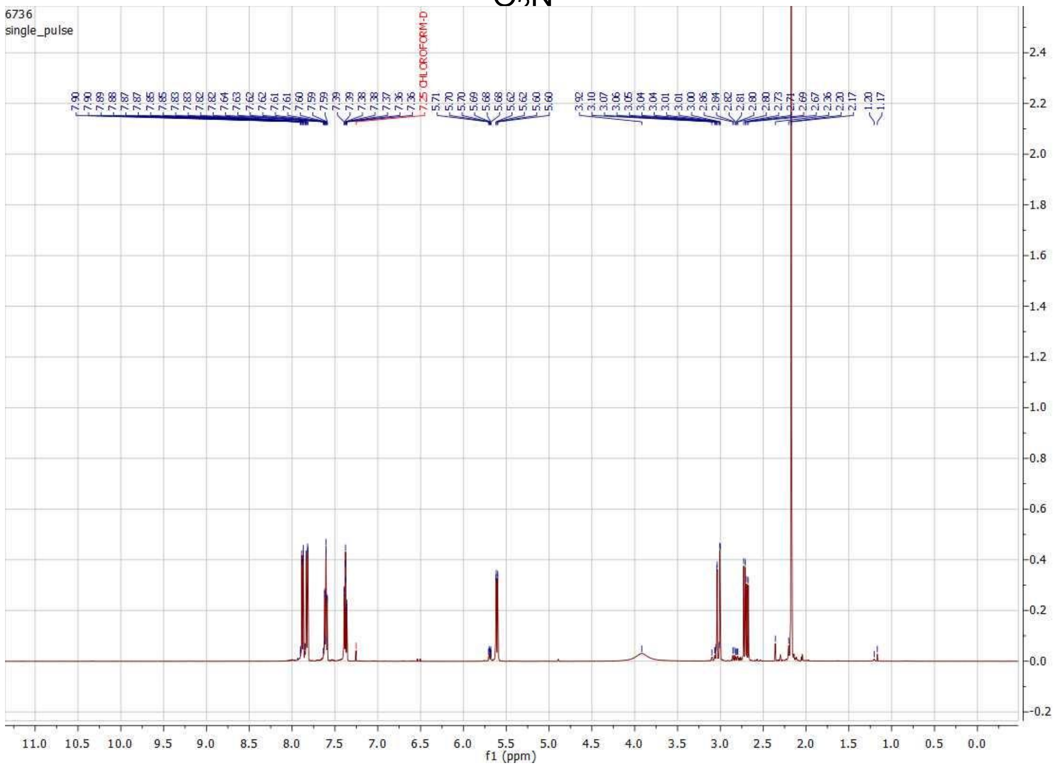

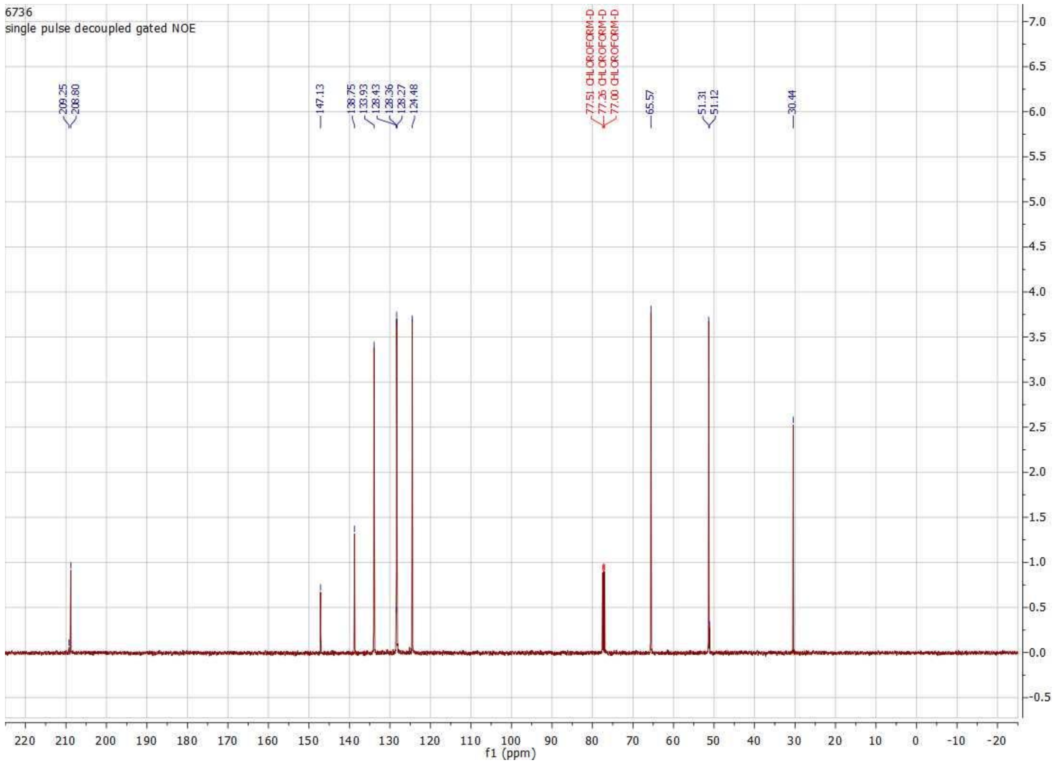

^{1}H and ^{13}C NMR of (*R*)**-29**

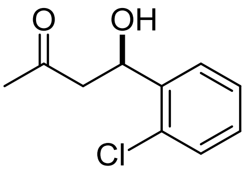

(*R*)-**29**

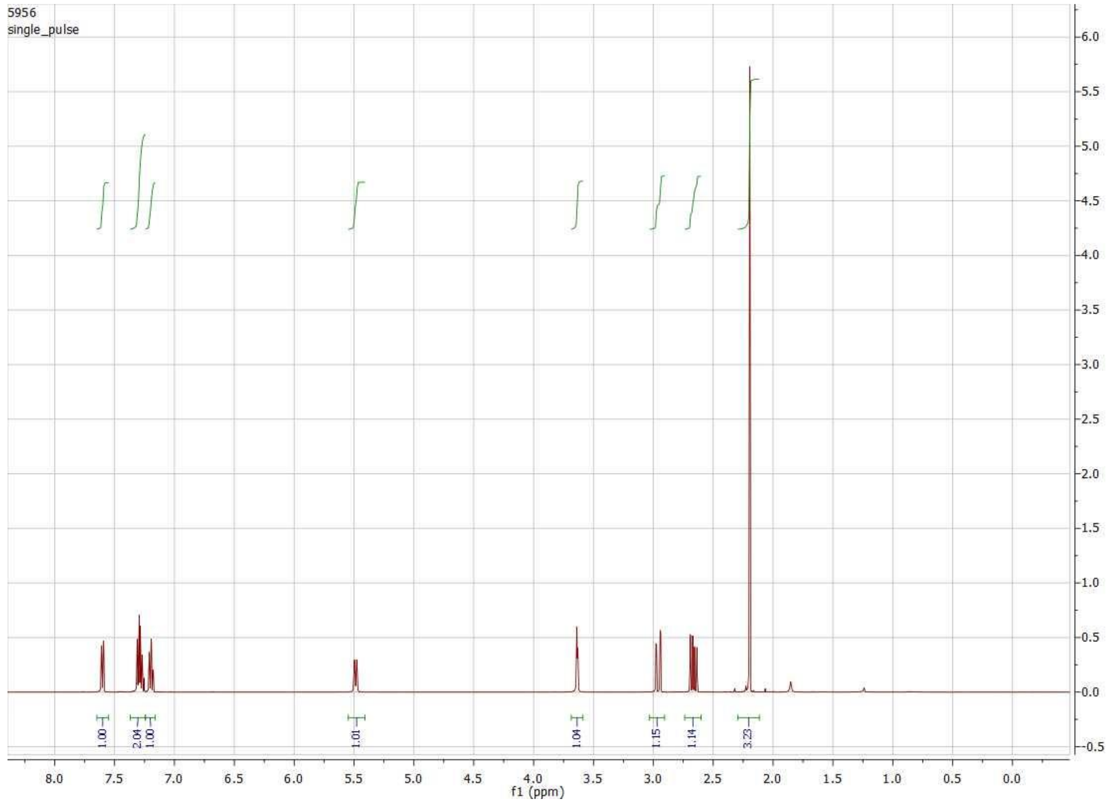

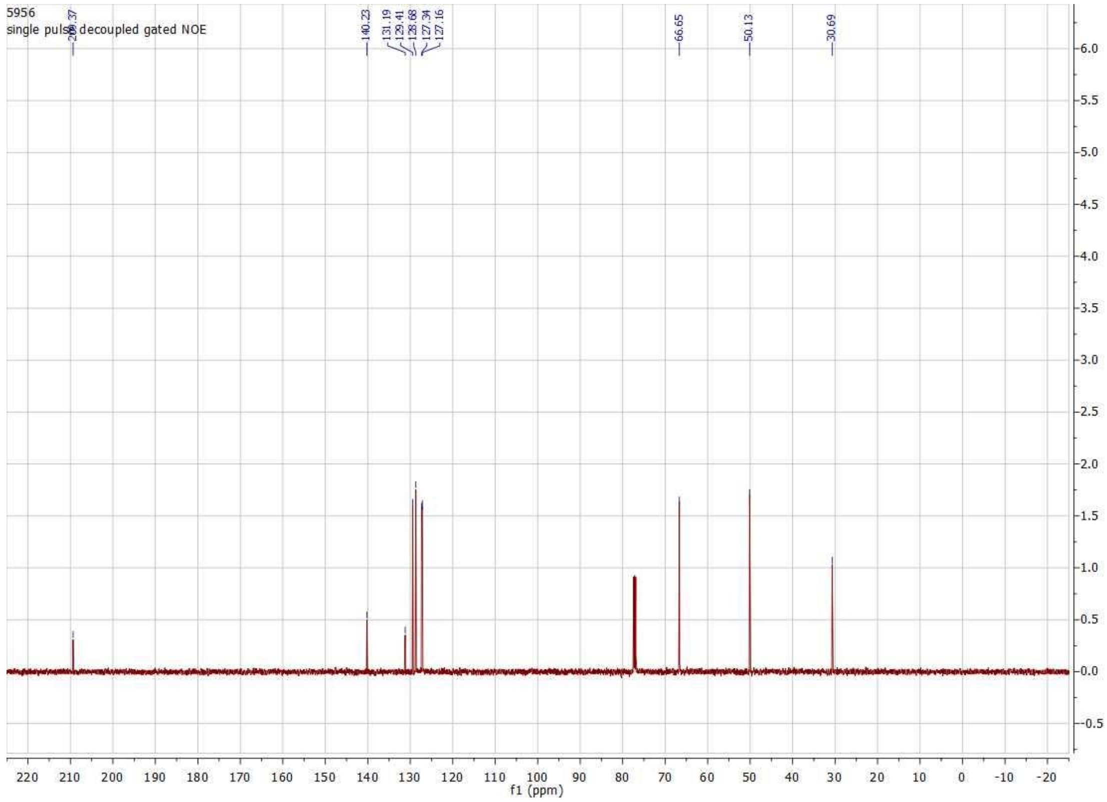

^{1}H and ^{13}C NMR of (*R*)**-30**

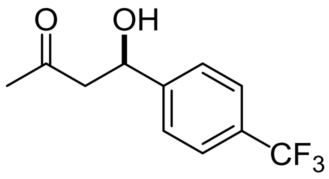

(*R*)-**30**

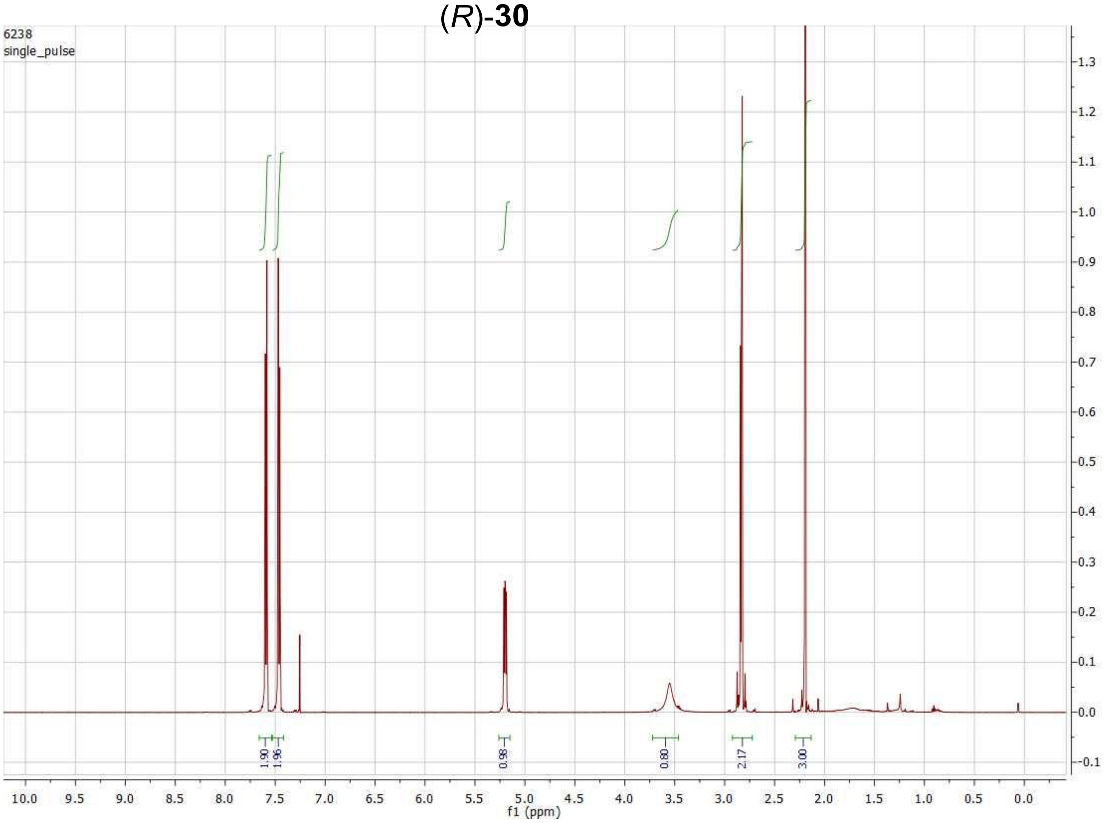

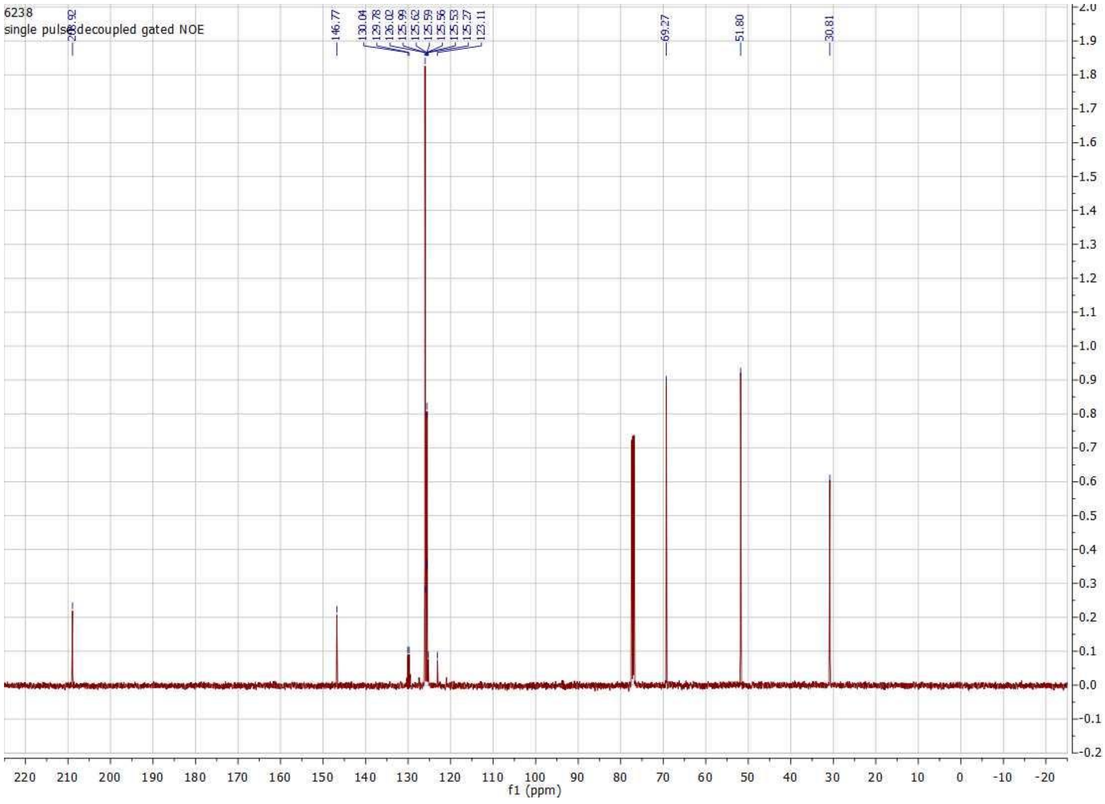

^{1}H NMR of (*R*)**-31**

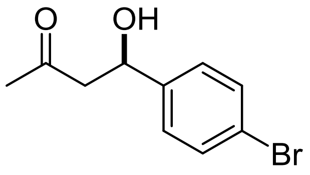

(*R*)-**31**

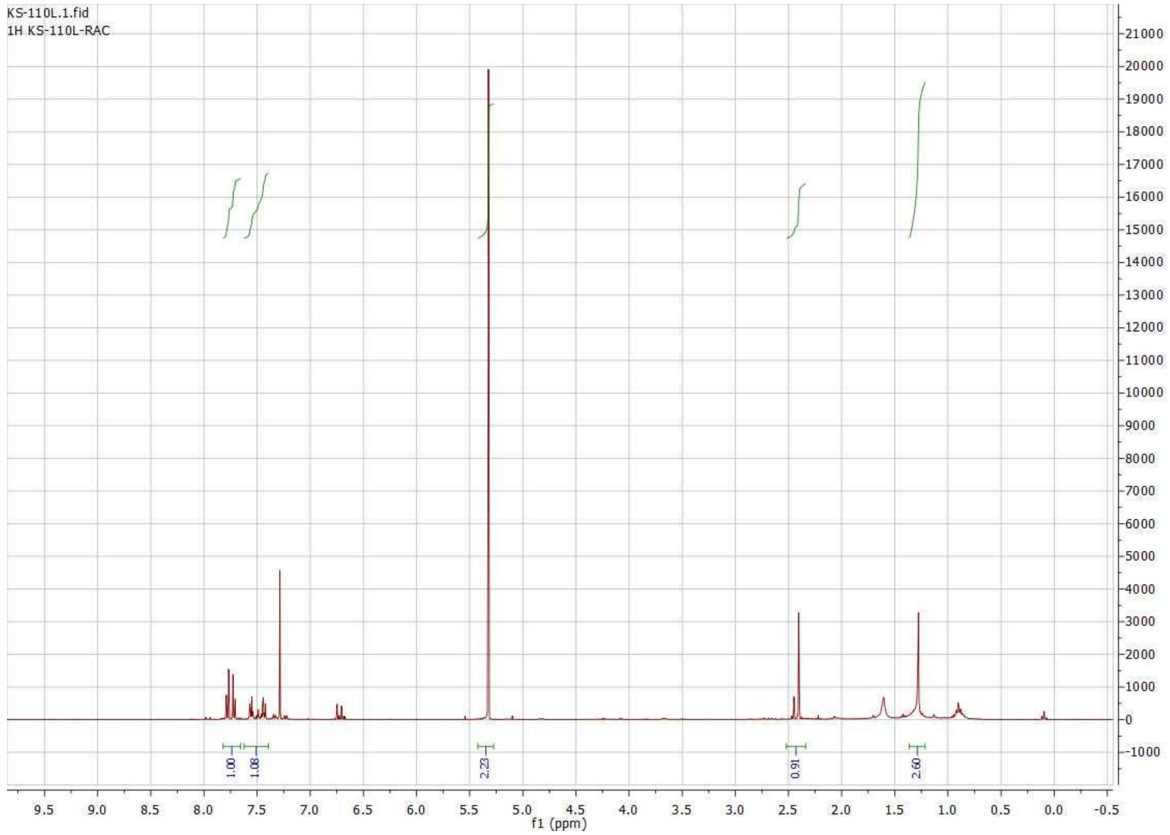

[1]H and ^{13}C of (*R*)**-32**

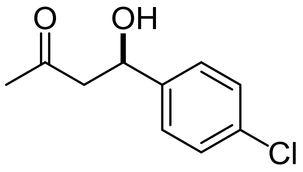

(*R*)-**32**

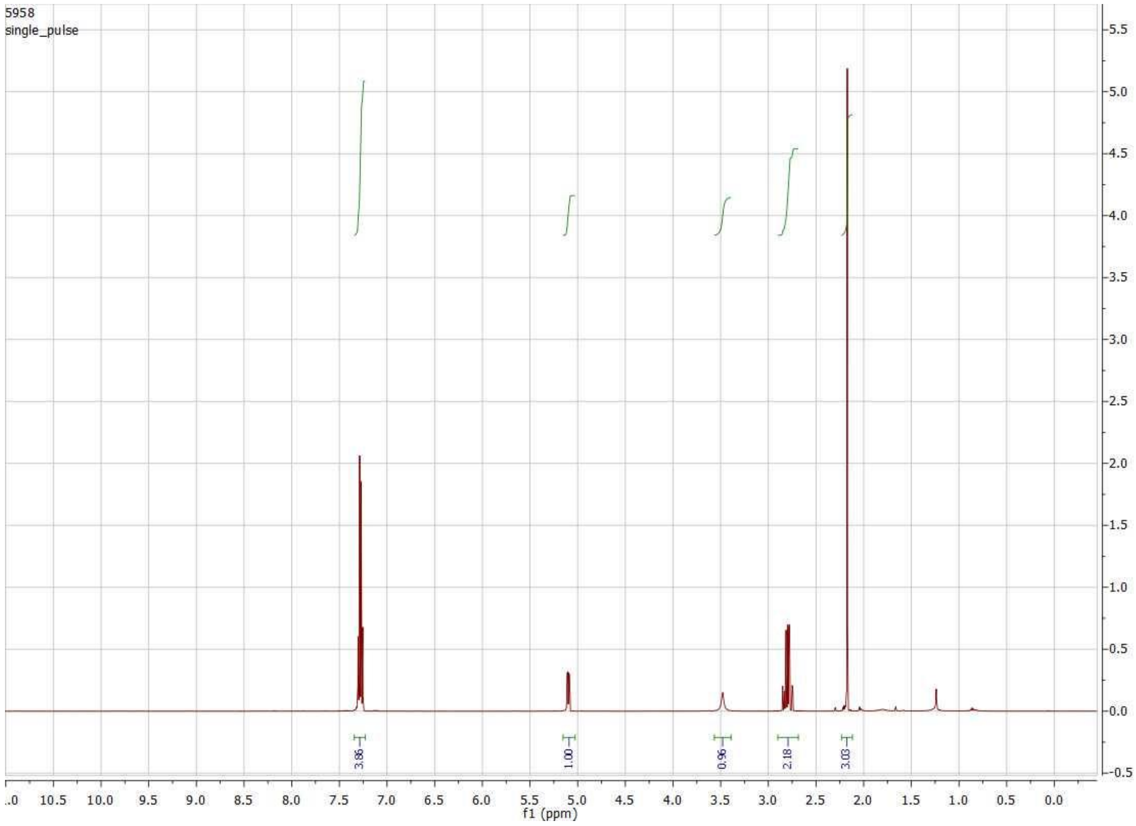

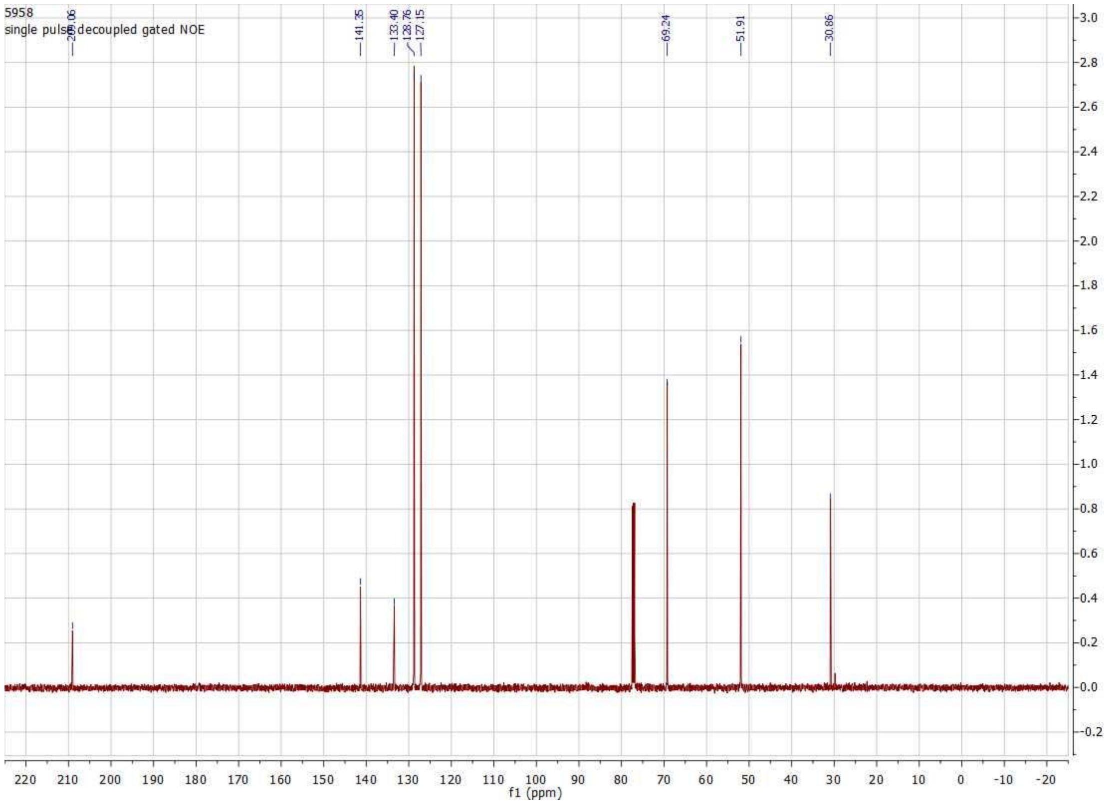

^{1}H and ^{13}C NMR of (*R*)-**33**

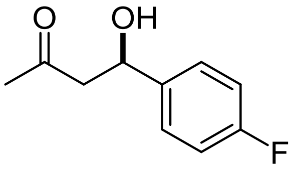

(*R*)-**33**

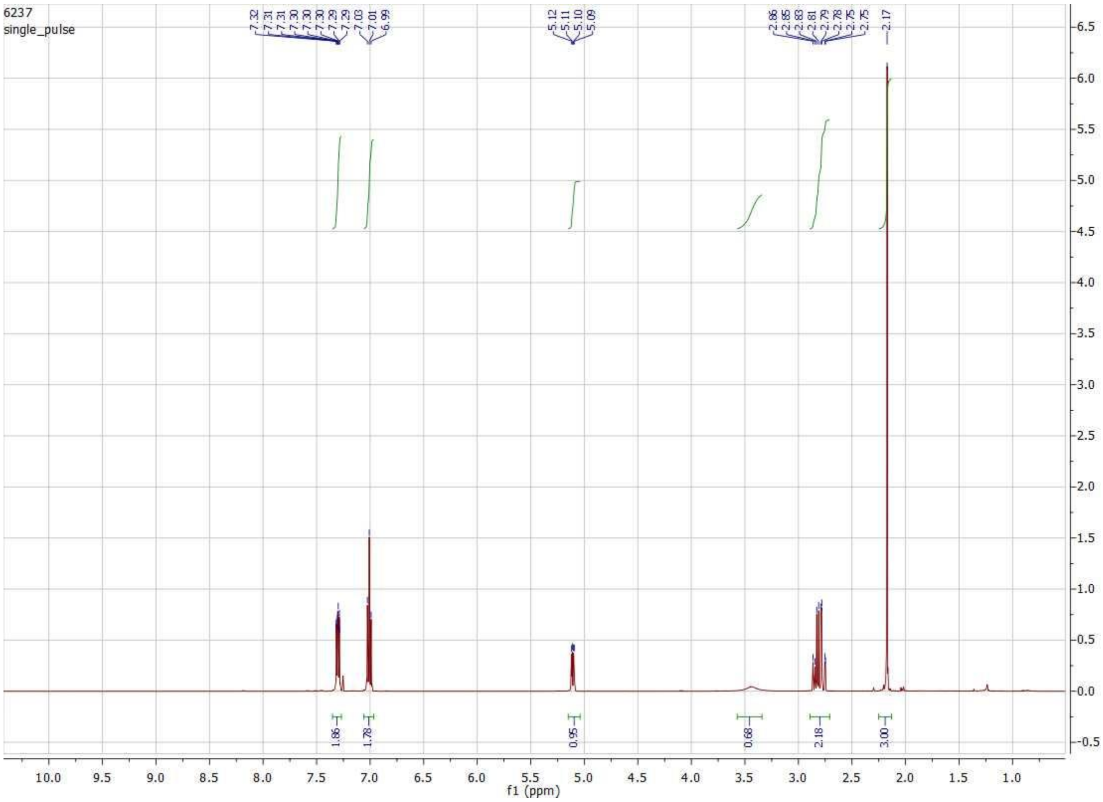

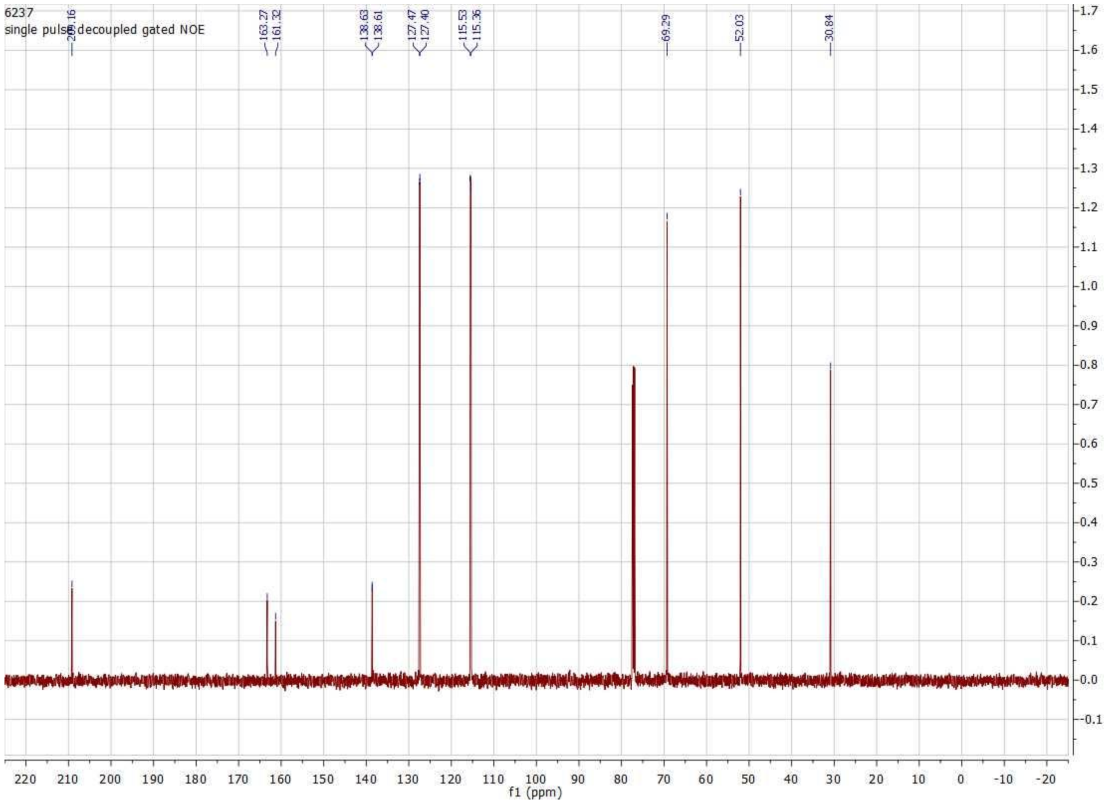

^{1}H and ^{13}C NMR of (*R*)-**34**

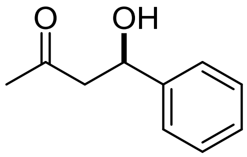

(*R*)-**34**

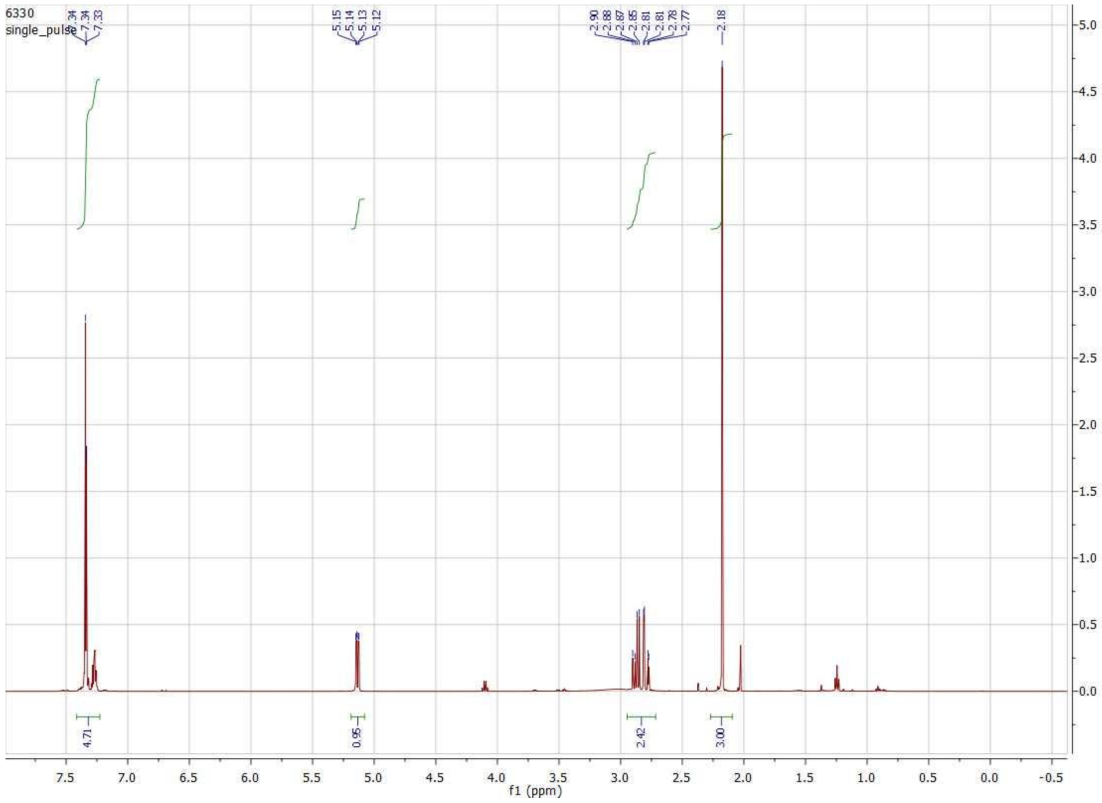

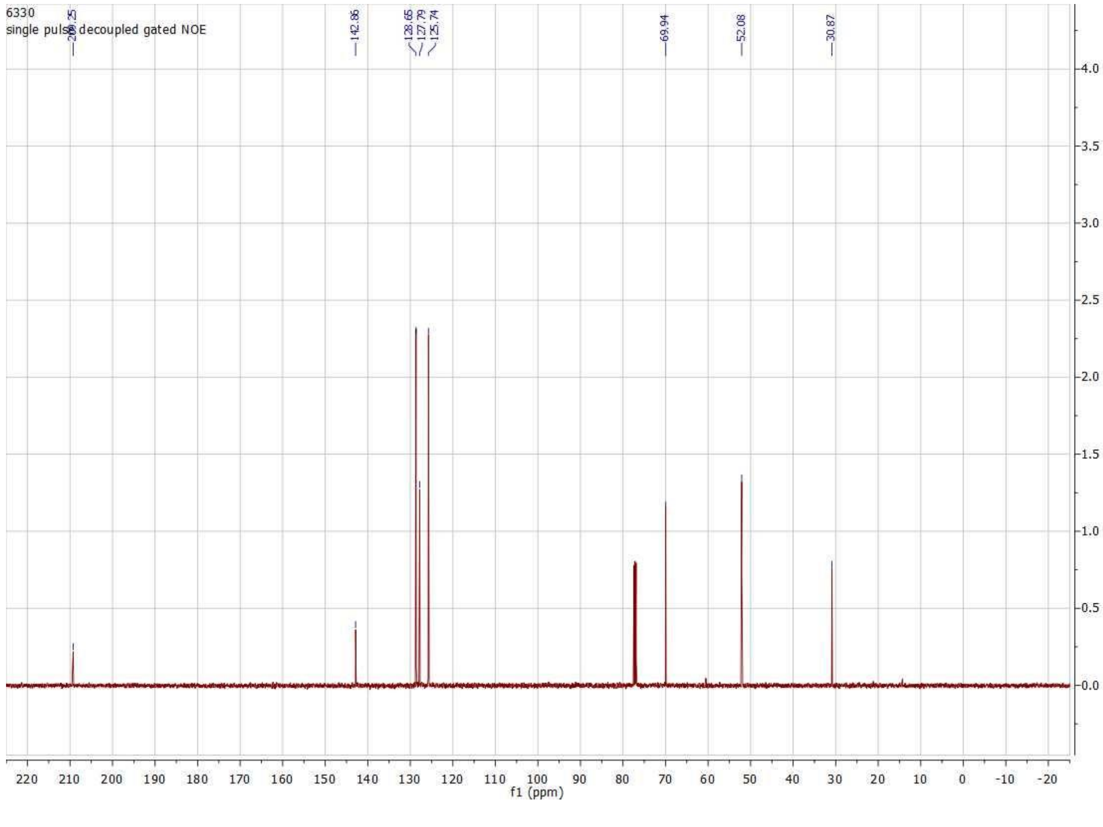

^{1}H NMR of (*R*)-**35**

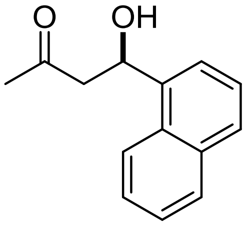

(*R*)-**35**

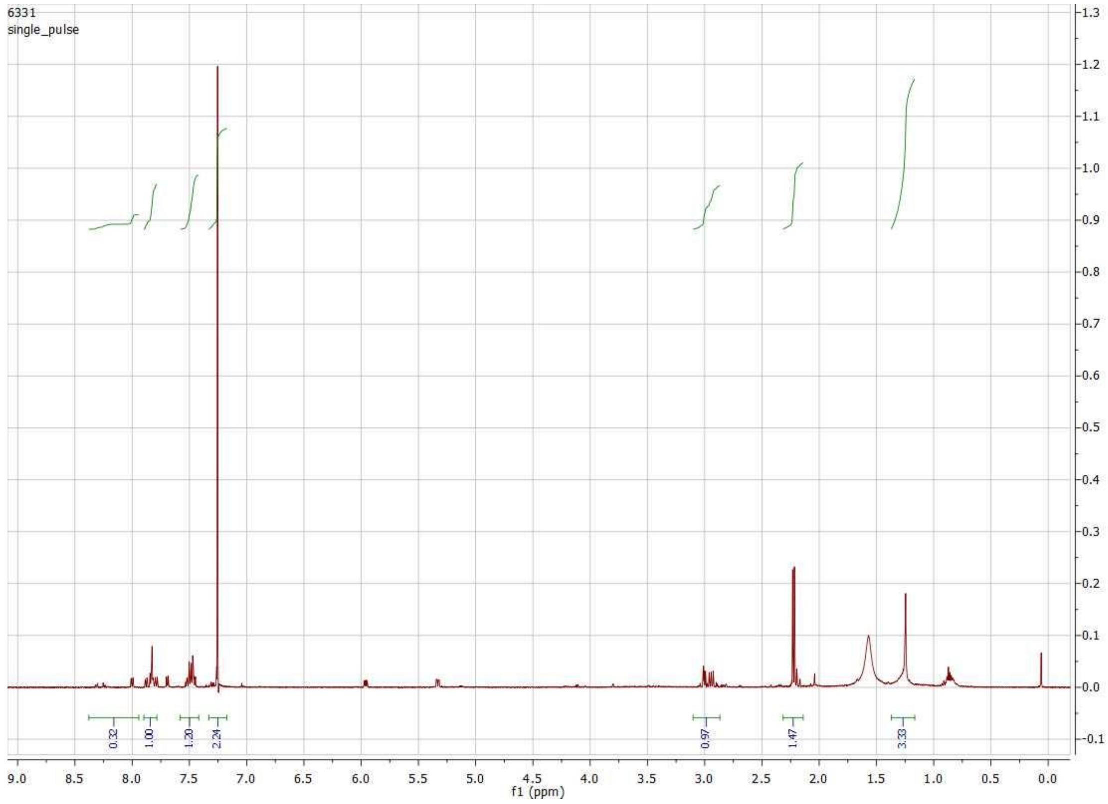

^{1}H and ^{13}C NMR of (*R*)**-36**

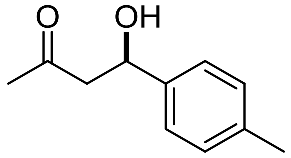

(*R*)-**36**

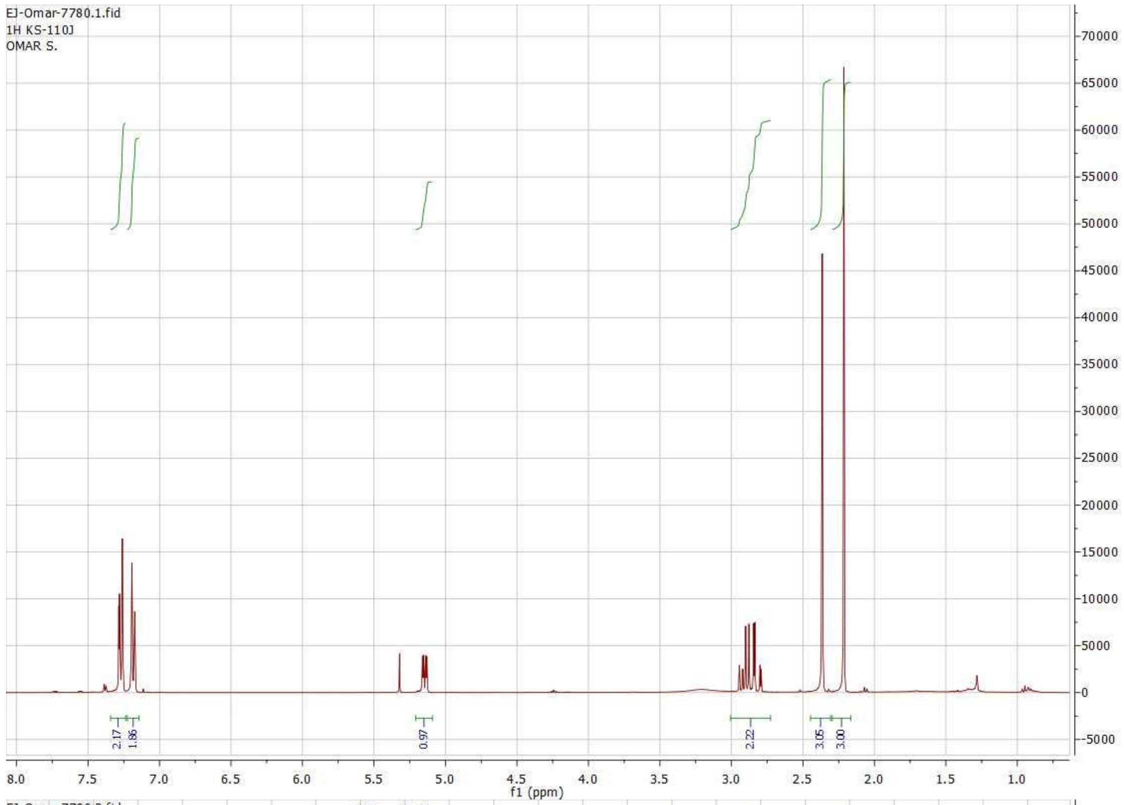

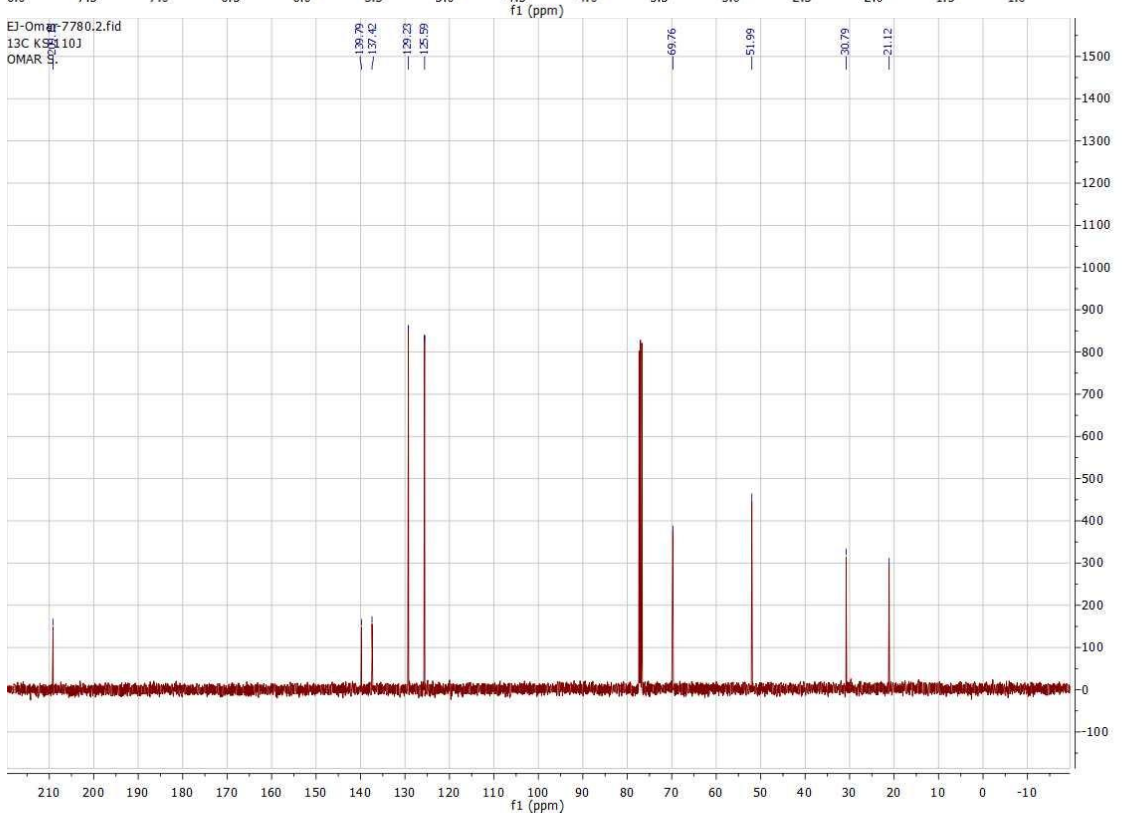

^{13}C NMR of (*R*)-**37**

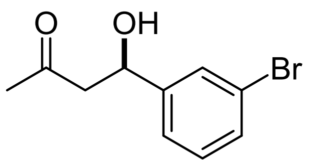

(*R*)-**37**

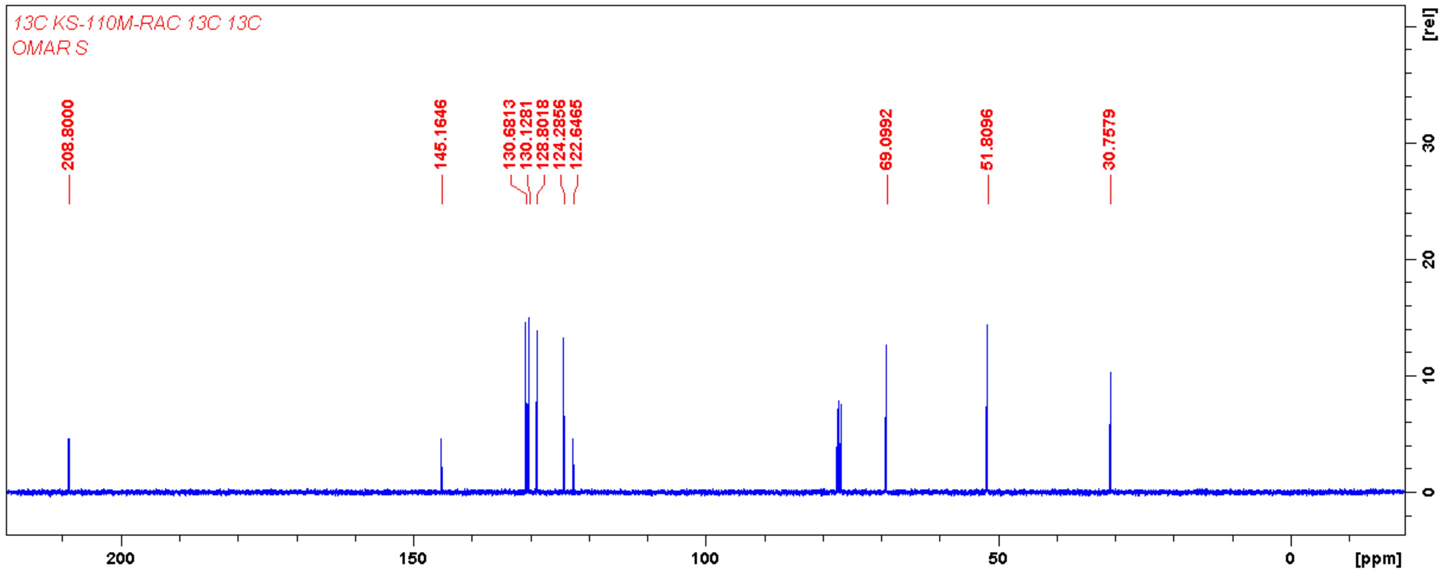

Aldol products with isatins.

^{1}H and ^{13}C NMR of (*R*,*S*)-**38**

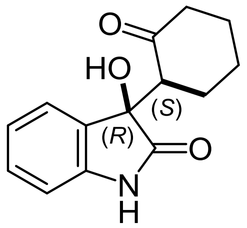

(*R*,*S*)–**38**

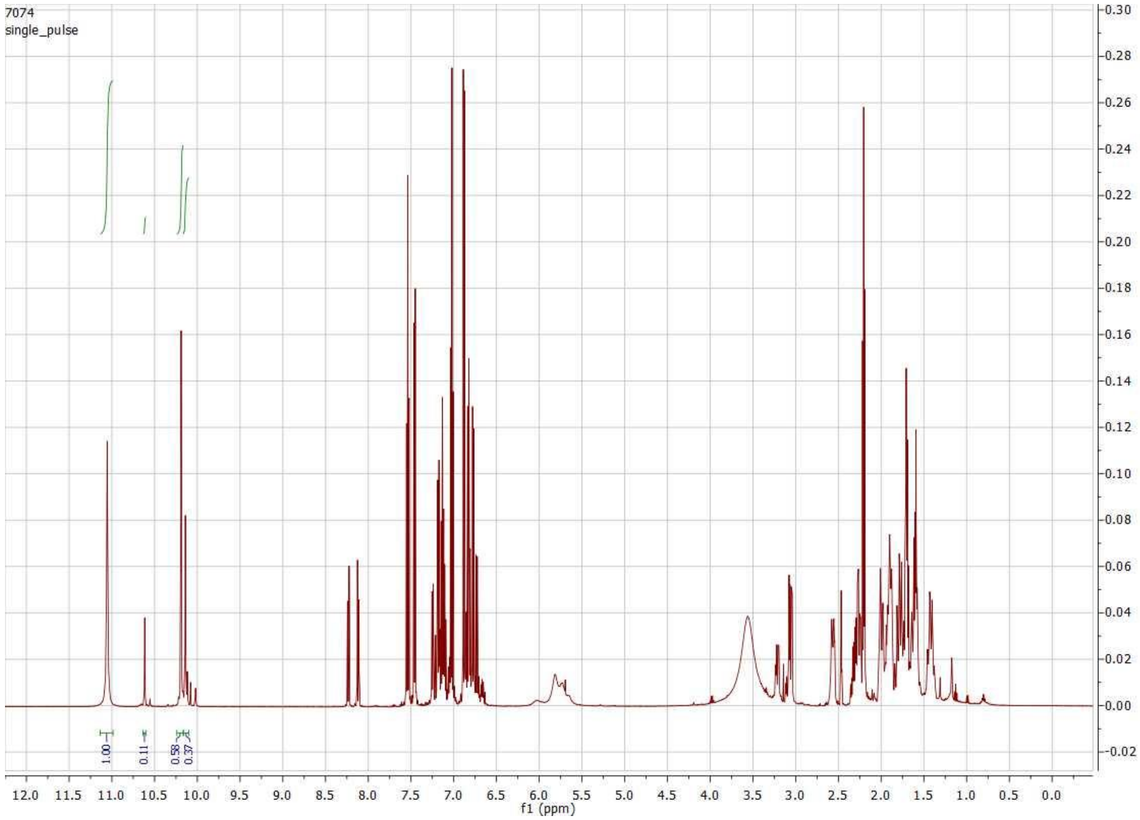

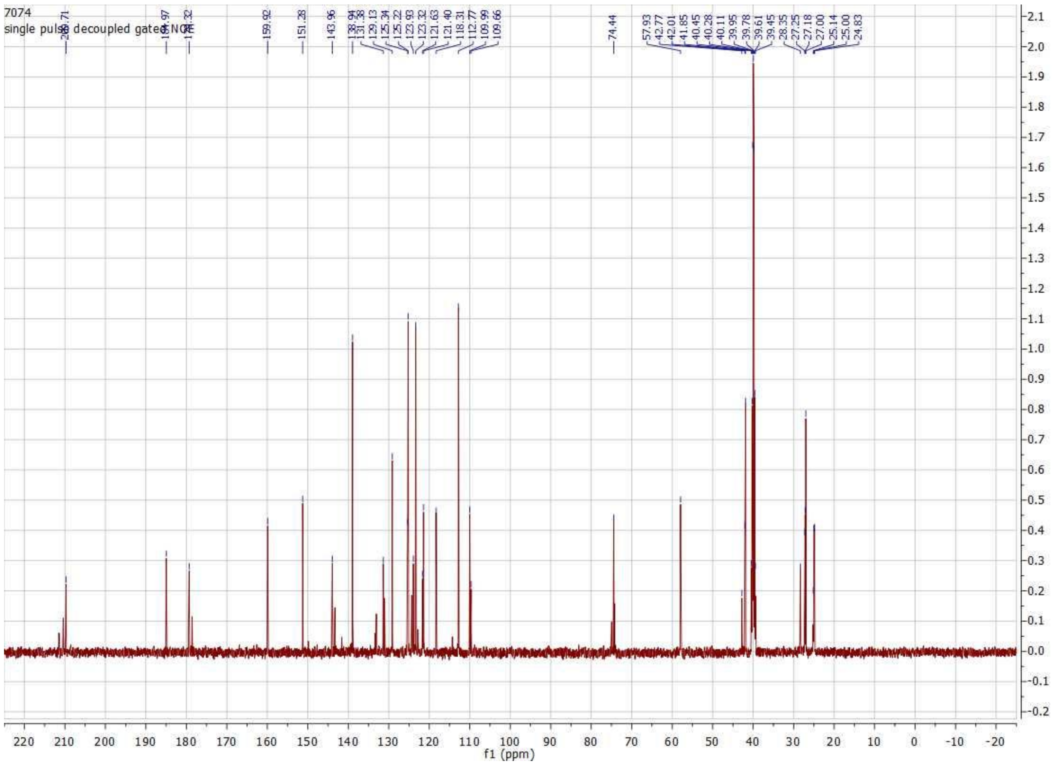

^{1}H and ^{13}C NMR of (*R*,*S*)-**39**

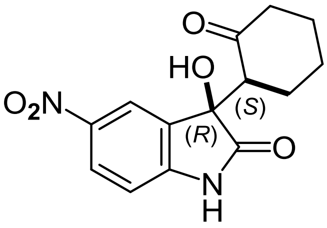

(*R*,*S*)–**39**

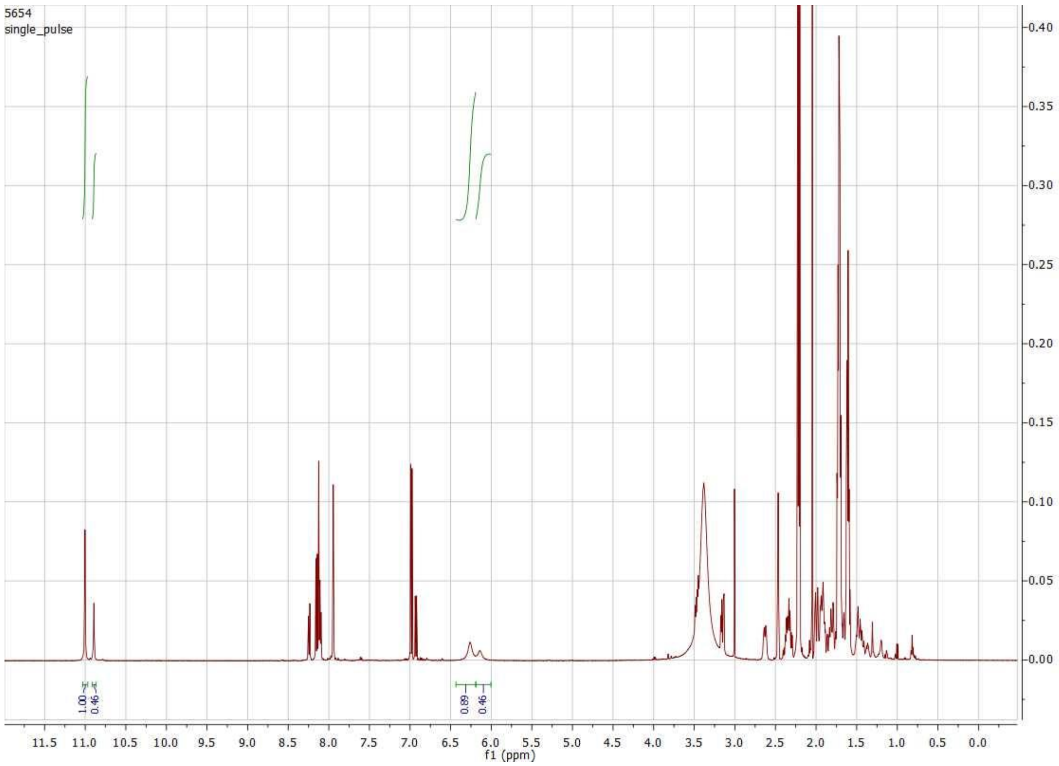

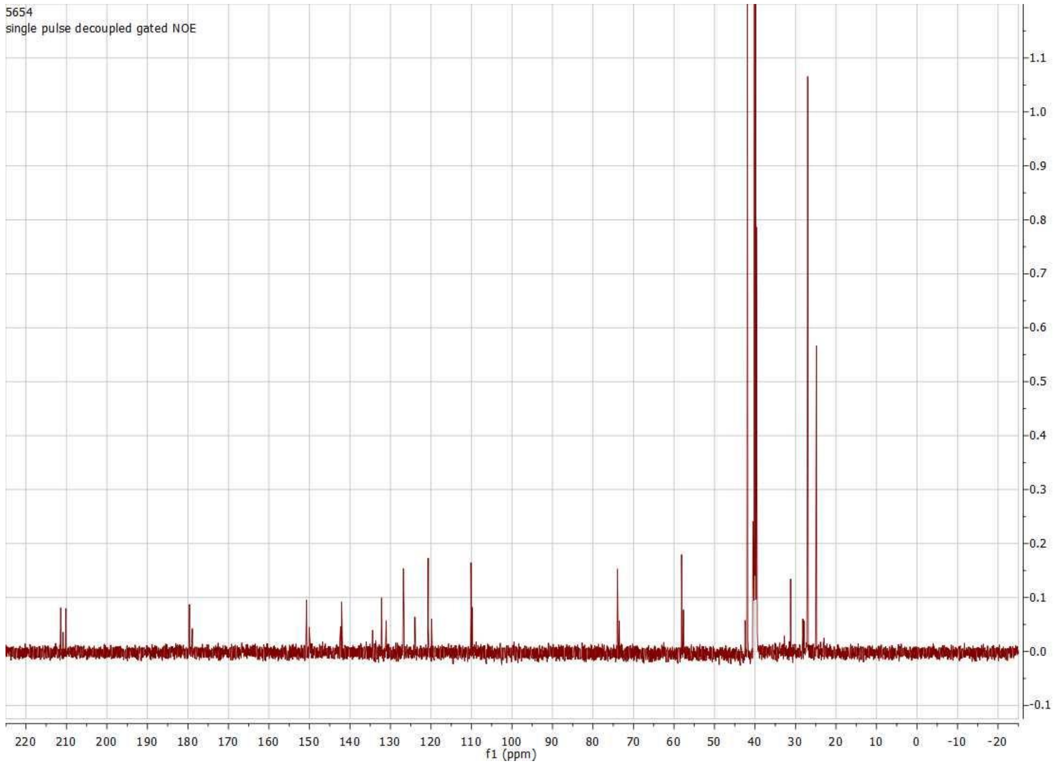

^1H and ^{13}C NMR of (*R*,*S*)-**40**

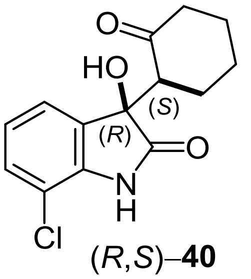

(*R*,*S*)–**40**

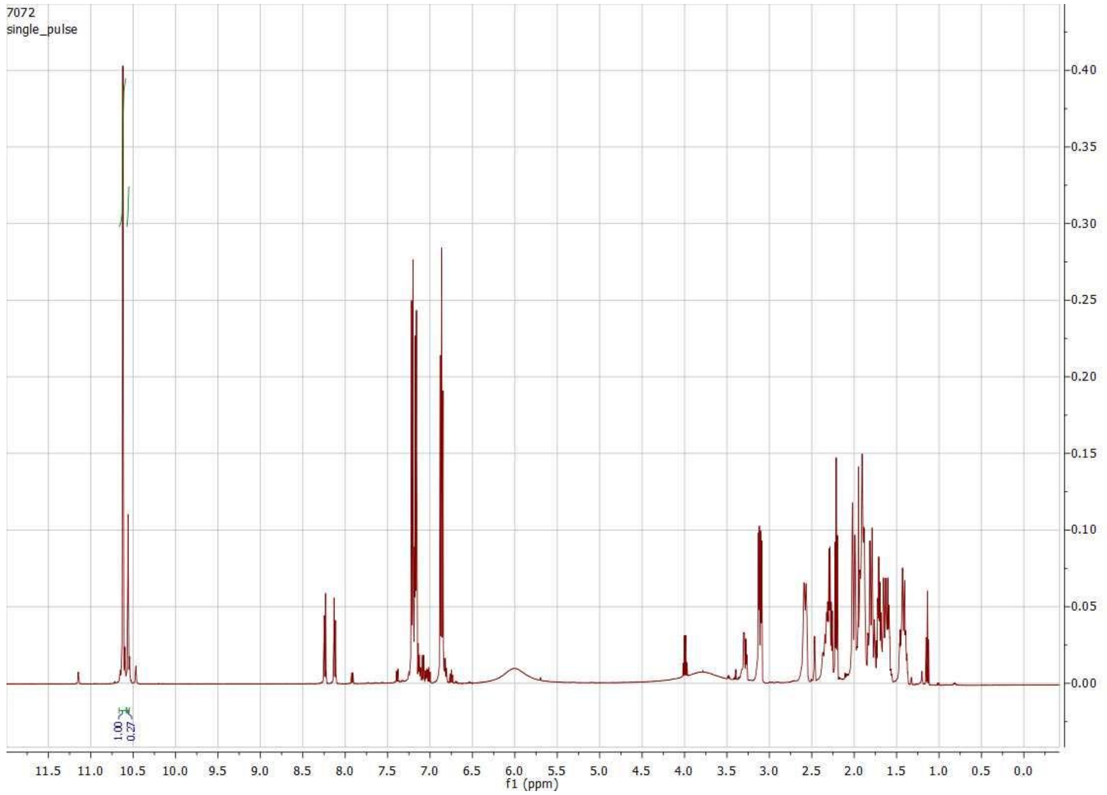

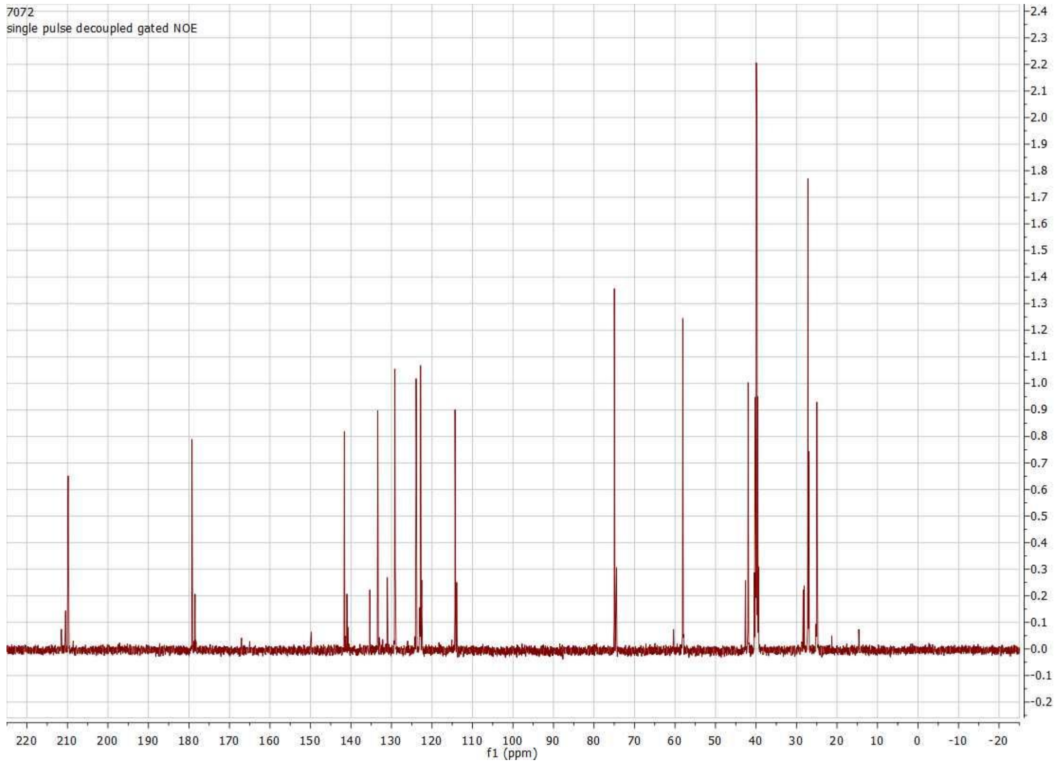

3. Gravimetry assays, TGA and DSG Analysis Data.

a) Gravimetry assays to calculate the amount of catalyst incorporated in the silica support.

via synthesis "in situ"

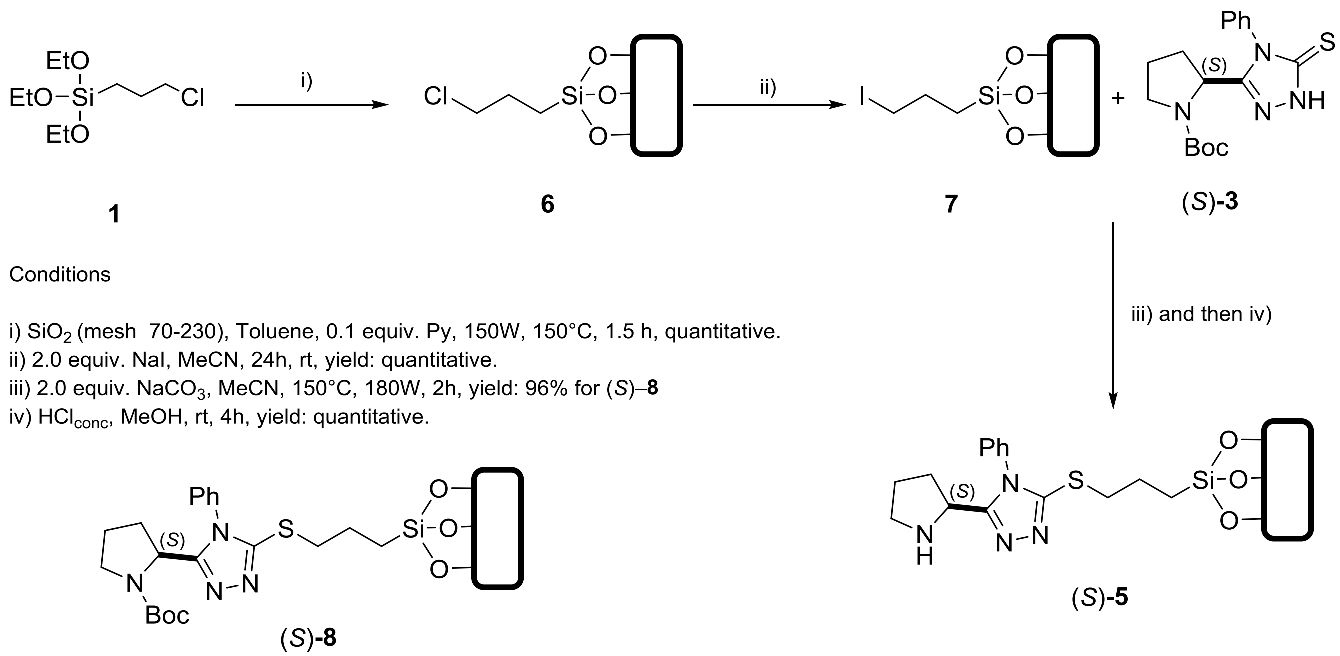

Silica (SiO_2) was activated for 48 hours at 250°C. The goal was to occupy the highest amount of functionalization sites, thus we saturated the silica (2.64 g) with **1** as shown in entry 4. 1.15 mmol of active sites could be functionalized. (Table S1).

Entry	1, mL (mmol)	% Yield
1	0.080 (0.30)	98
2	0.132 (0.50)	98
3	0.264 (1.00)	98
4	0.528 (2.00)	59
5	0.316 (1.2)	96

b) TGA and DSG analysis data

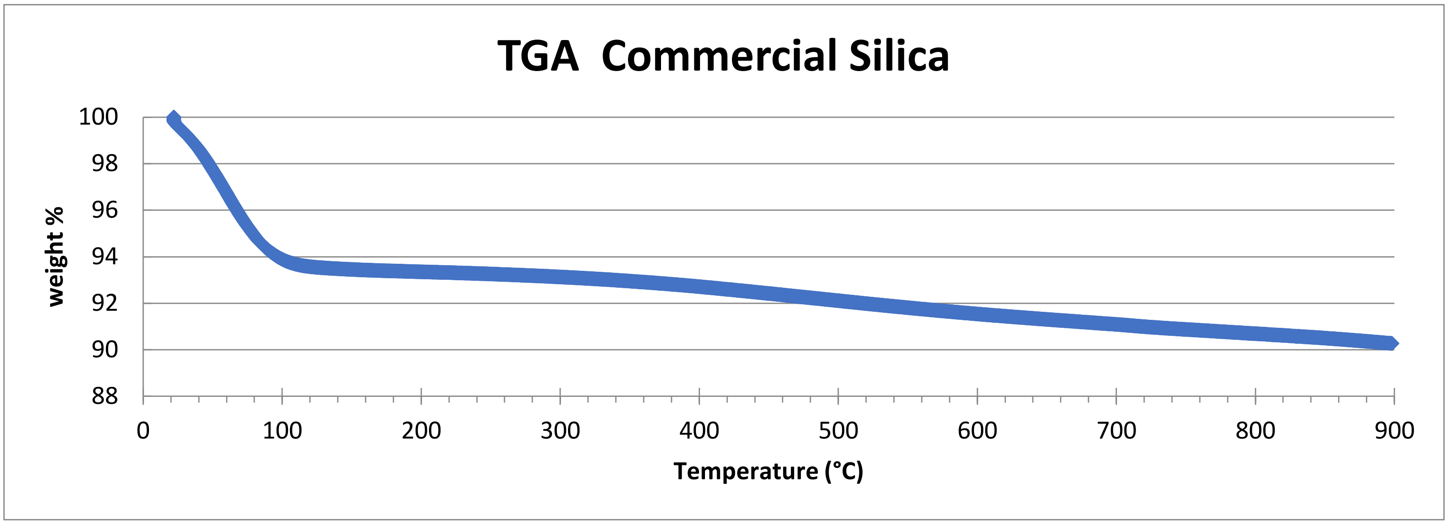

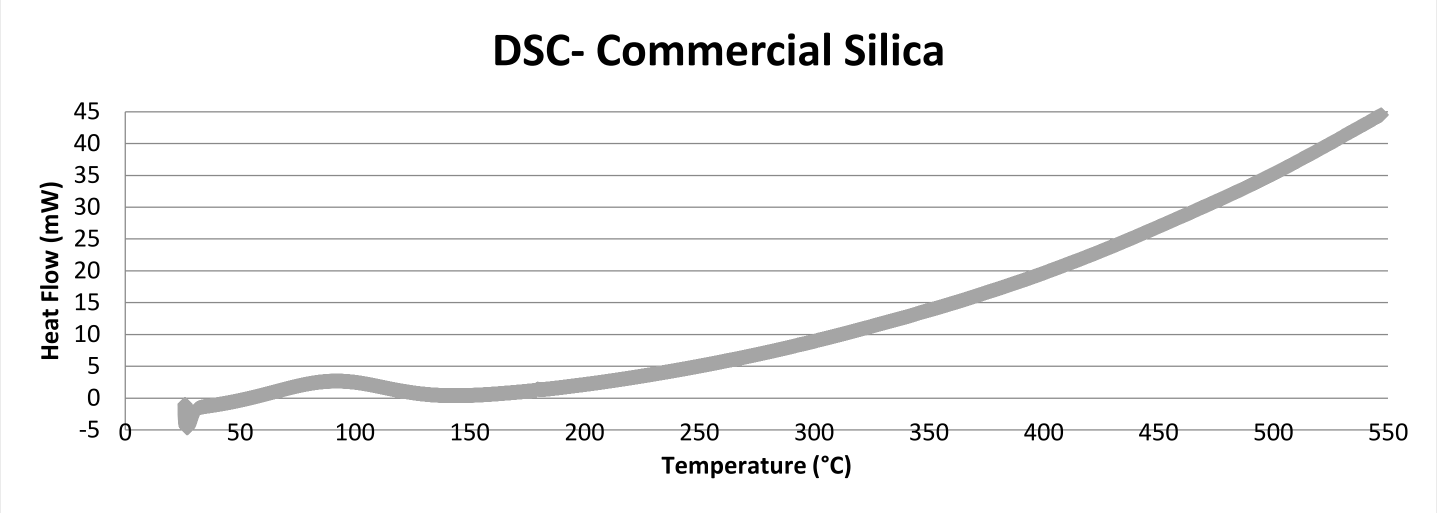

Commercial silica, initial weight, 8.807 mg

(*S*)–**5,** initial weight: 6.313 mg

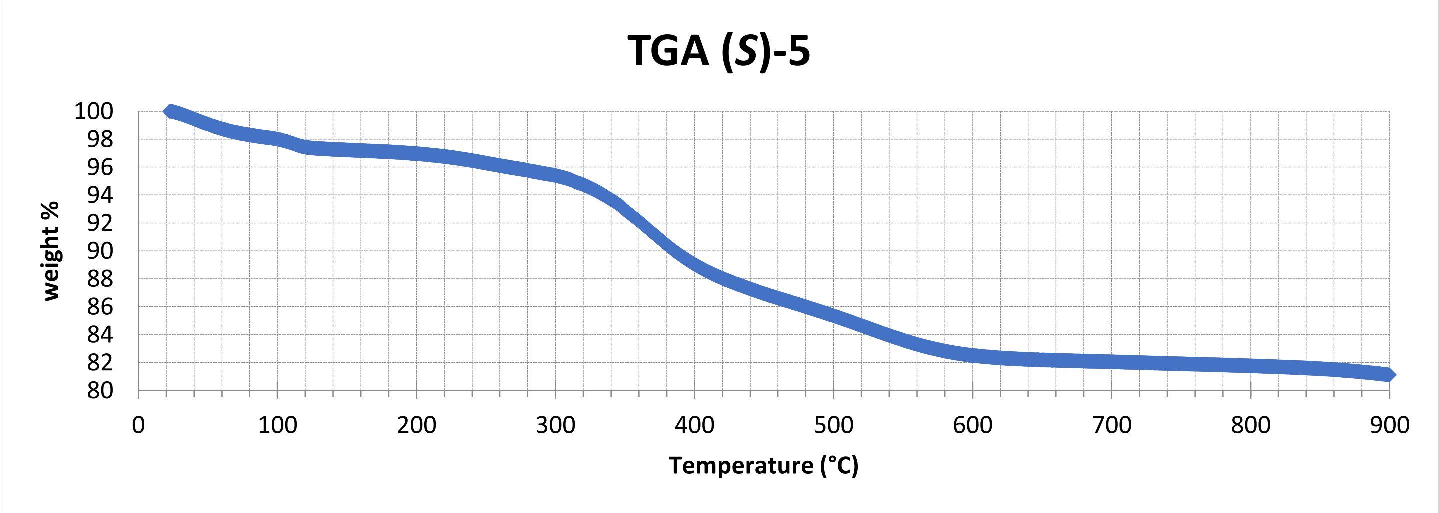

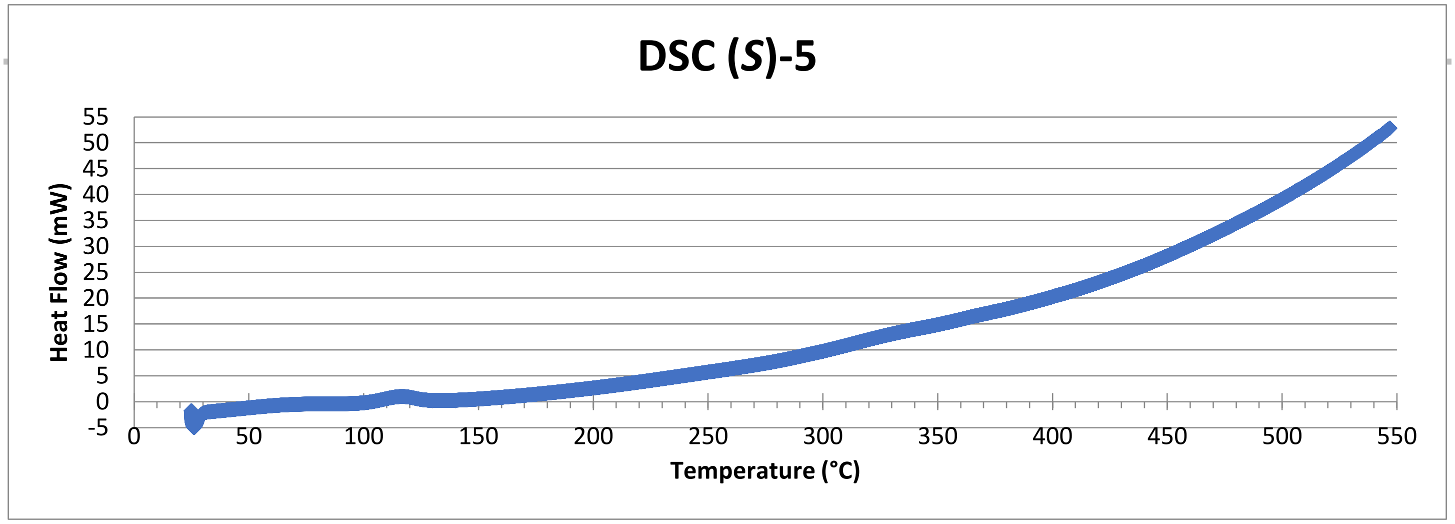
DSC (*S*)-5
Heat Flow (mW)
55
50
45
40
35
30
25
20
15
10
5
0
-5
0
50
100
150
200
250
300
350
400
450
500
550
Temperature (°C)

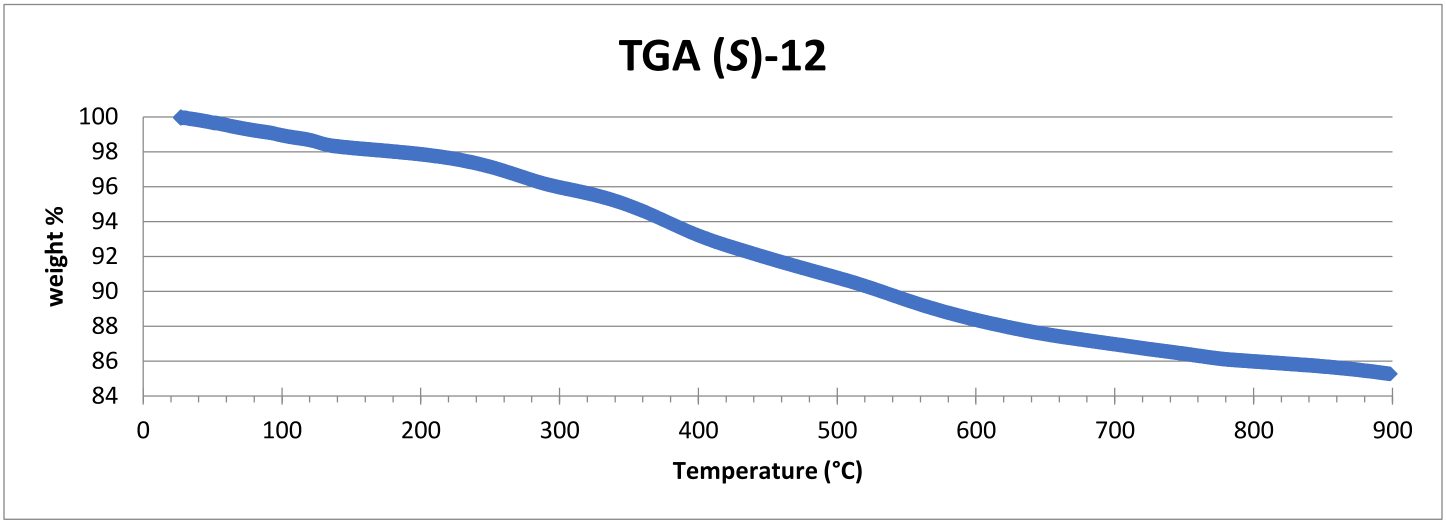
TGA (*S*)-12
weight %
100
98
96
94
92
90
88
86
84
0
100
200
300
400
500
600
700
800
900
Temperature (°C)

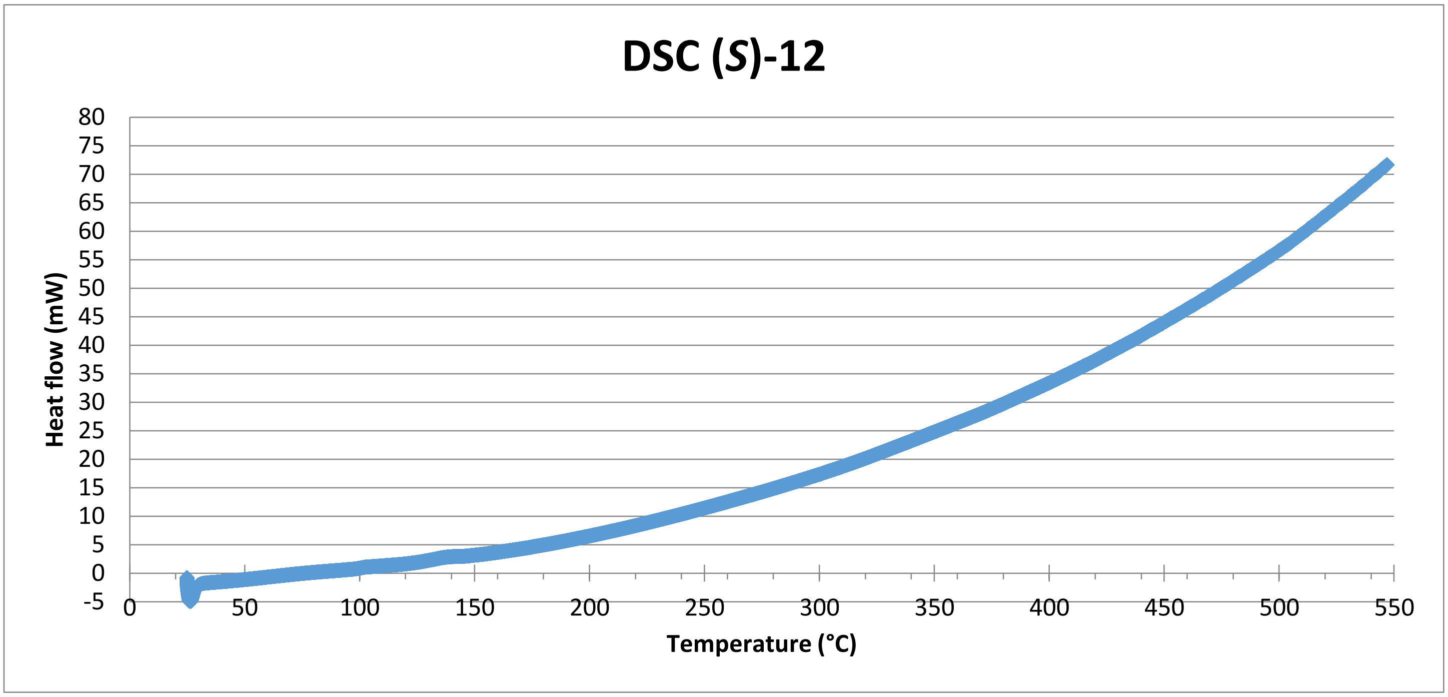
DSC (*S*)-12
Heat flow (mW)
80
75
70
65
60
55
50
45
40
35
30
25
20
15
10
5
0
-5
0
50
100
150
200
250
300
350
400
450
500
550
Temperature (°C)

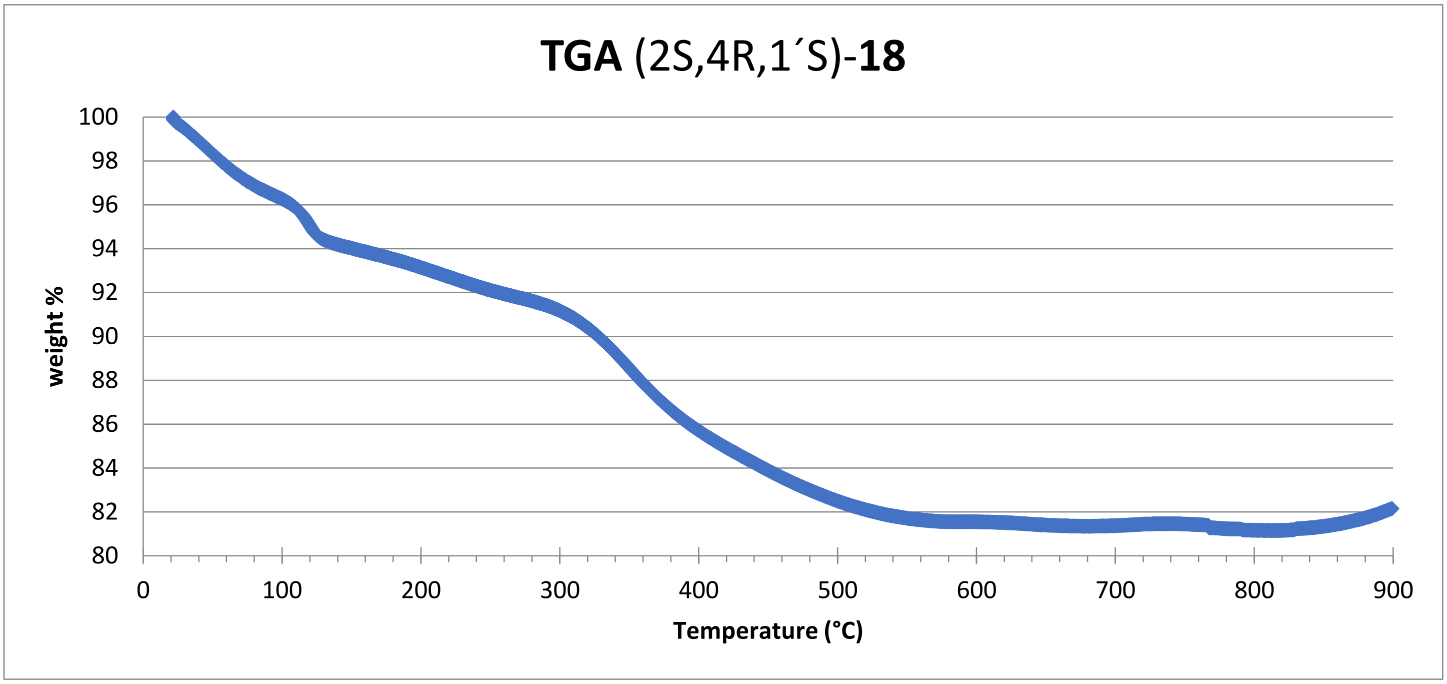
TGA (2S,4R,1´S)-18
weight %
100
98
96
94
92
90
88
86
84
82
80
0
100
200
300
400
500
600
700
800
900
Temperature (°C)

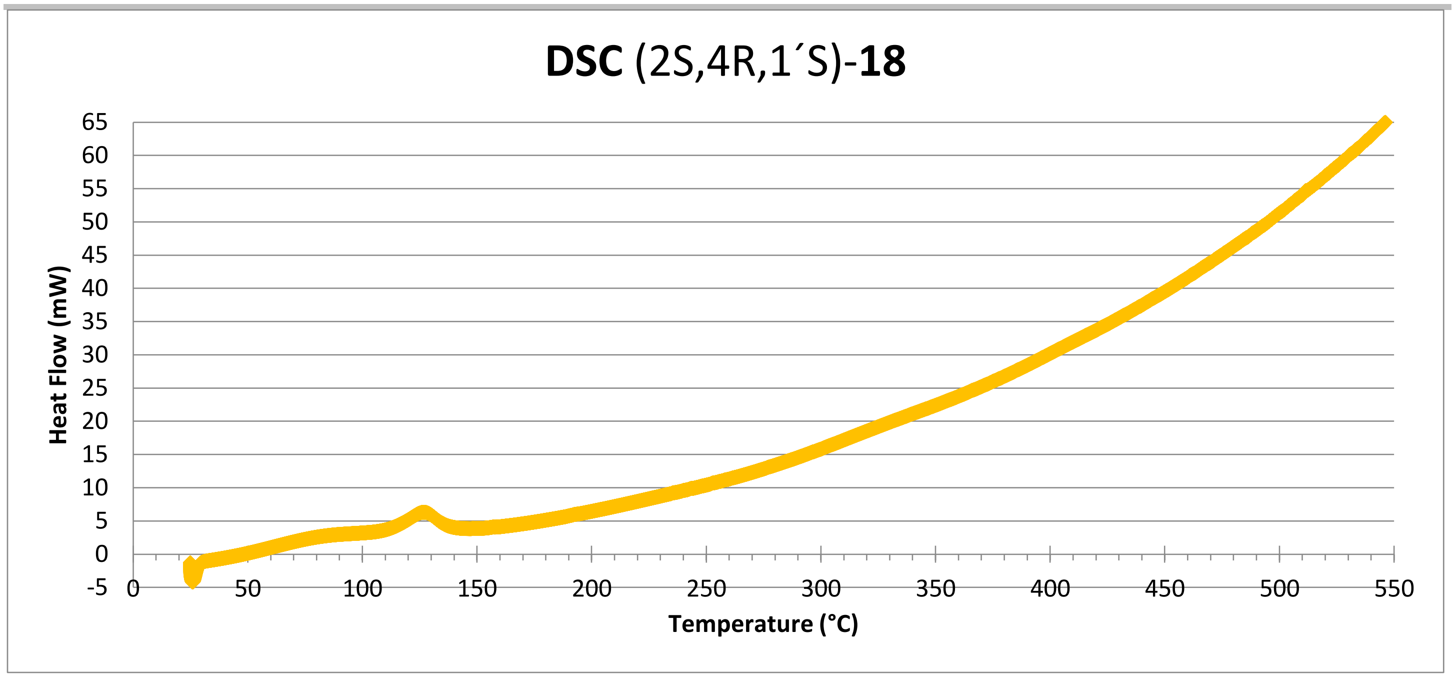
DSC (2S,4R,1´S)-18
Heat Flow (mW)
65
60
55
50
45
40
35
30
25
20
15
10
5
0
-5
0
50
100
150
200
250
300
350
400
450
500
550
Temperature (°C)

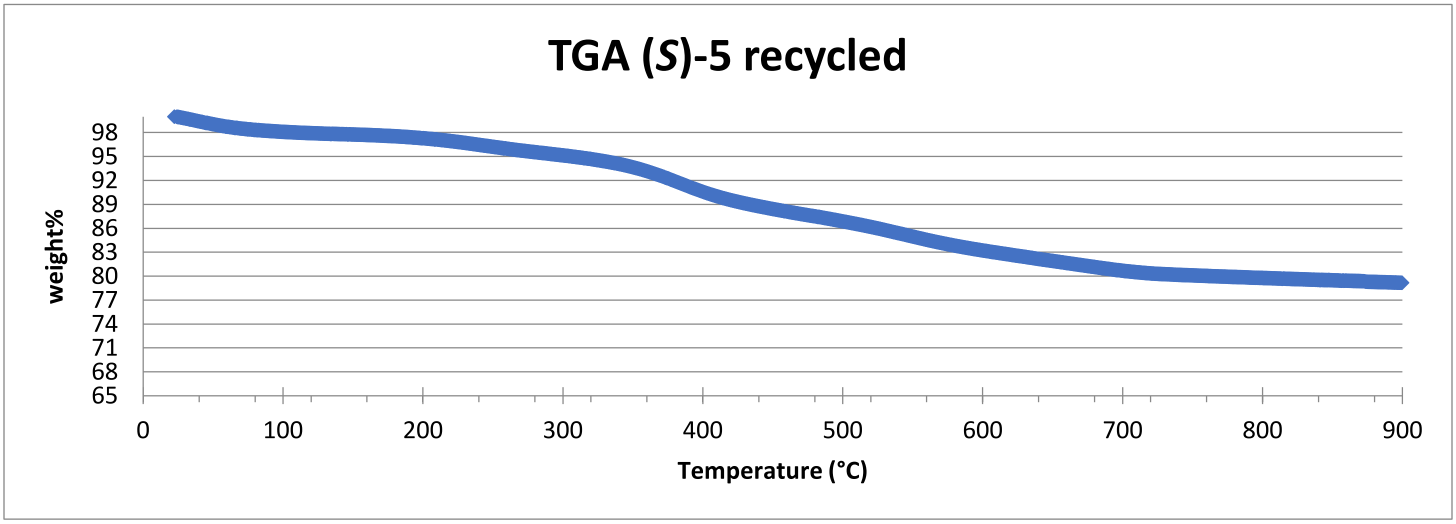
TGA (S)-5 recycled
weight%
98
95
92
89
86
83
80
77
74
71
68
65
0
100
200
300
400
500
600
700
800
900
Temperature (°C)

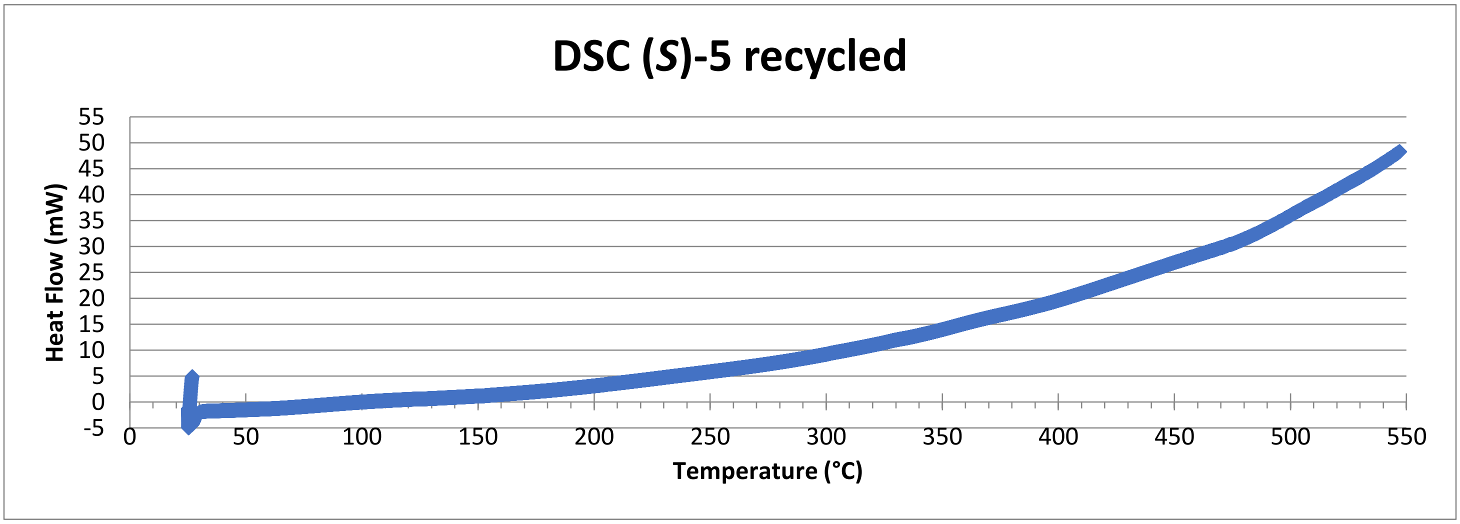
DSC (S)-5 recycled
Heat Flow (mW)
55
50
45
40
35
30
25
20
15
10
5
0
-5
0
50
100
150
200
250
300
350
400
450
500
550
Temperature (°C)

Table S2: TGA analysis for determining organocatalyst amount per gram of silica.

Entry	Compound	Weight of sample (mg)	H_2O % (mg)	Organic % (mg)	organocatalyst bonded to silica ratio, mmol/g, (mg/g)
**1**	Commercial Silica [7]	8.807	6.12, (0.539)	3.61, (0.312)	0.0, (0.0)
2	**(*S*)**–5	6.315	2.05, (0.129)	13.38 (1.068-0.223 = 0.8446)	0.4641, (114.6)
3	**(*S*)**–12	5.014	1.11, (0.056)	13.65, (0.6846-0.177 = 0.507)	0.4245, (48.33)
4	**(2*S*,4*R*, *S*)**–18	3.882	3.735, (0.151)	14.09, (0.5469-0.137 = 0.410)	0.2843, (66.32)
5	**(*S*)**–5 washed and recovery form.	3.463	2.01, (0.0694)	18.92, (0.6554-0.122 = 0.533)	0.5339, (131.95)

Condition in which TGA was carried out:

Temperature range	Heating ratio	Gals fluxing
25 - 900 °C	20 °C/min	25 mL/min

Condition in which DSC was recorded:

Temperature range	Heating ratio	Gals fluxing
25 - 550 °C	20 °C/min	25 mL/min

4.2. FT-IR analysis.

4.1 Commercial Silica, SiO_2

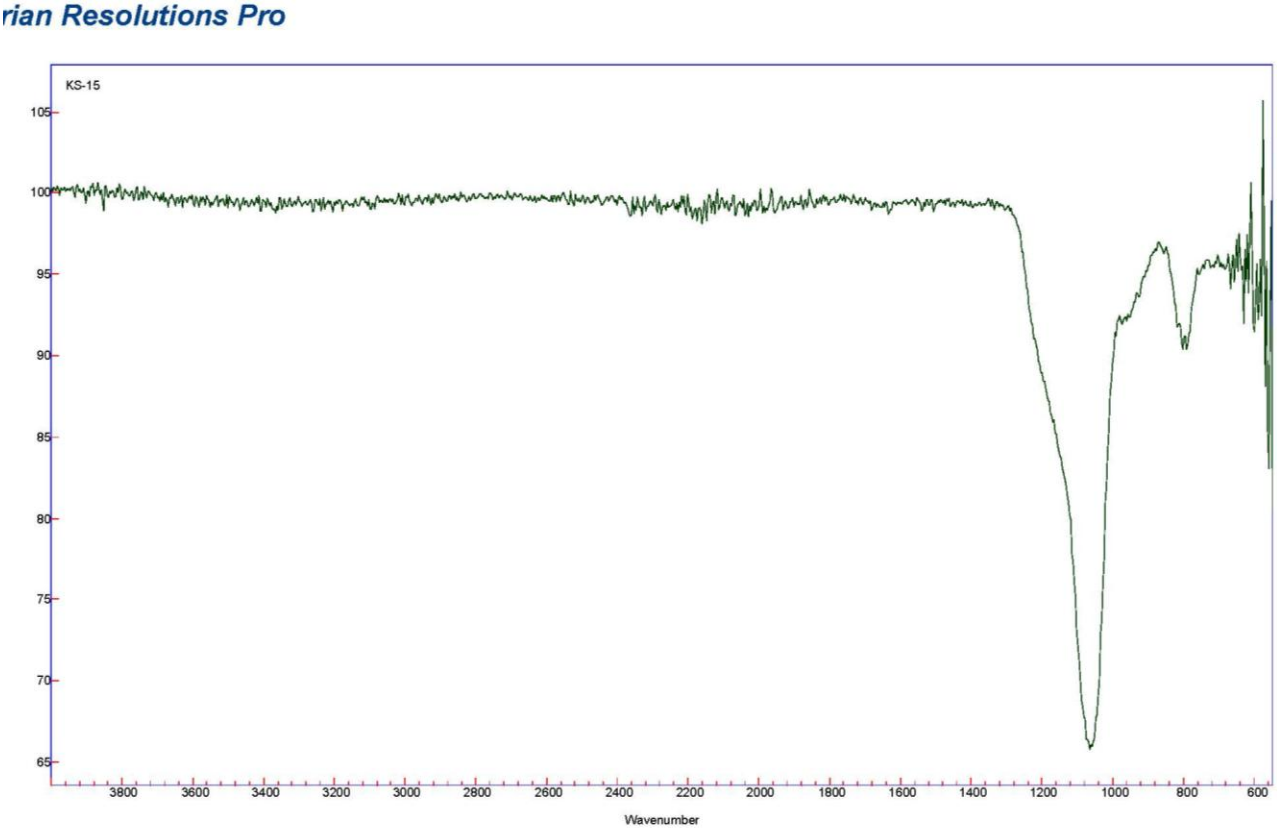

4.2 (*S*)**-5**

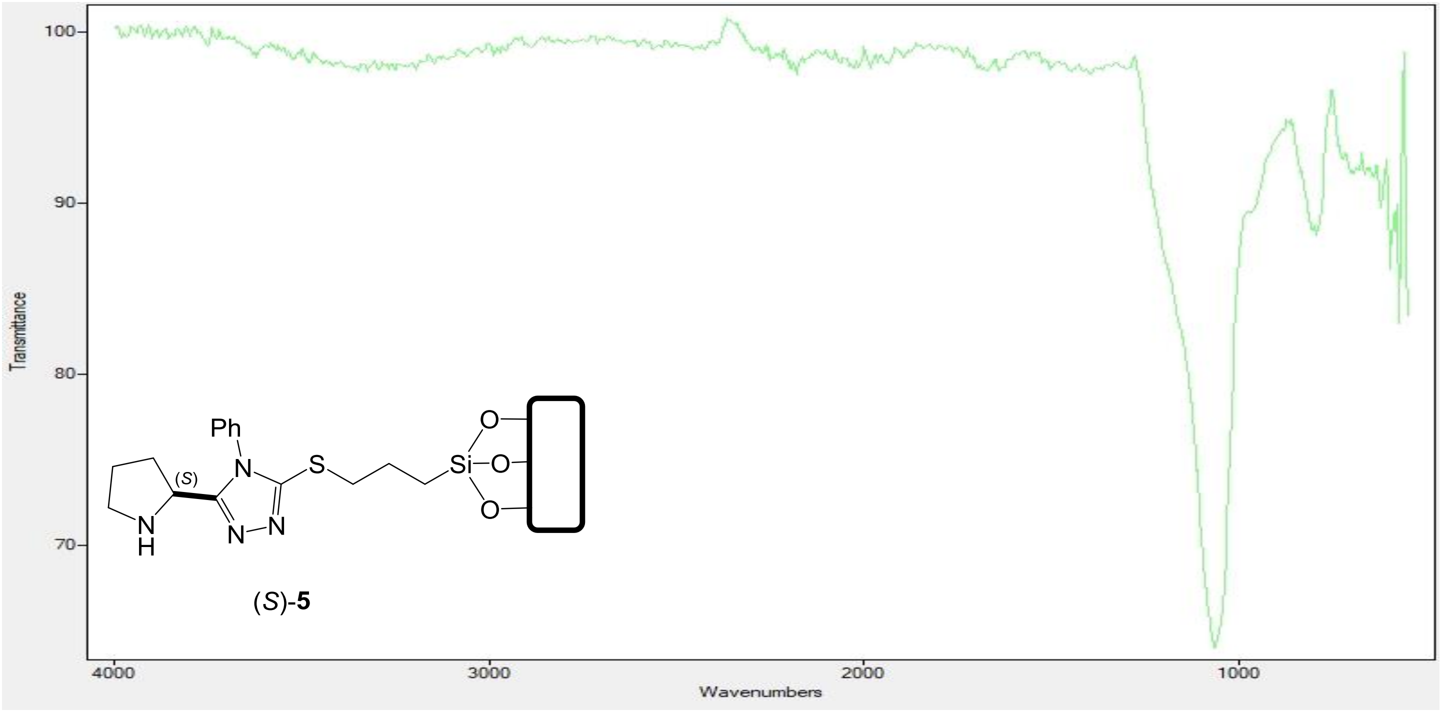

100
90
80
70
Transmittance
4000
3000
2000
1000
Wavenumbers
Ph
N
(S)
S
Si
O
O
O
N
H
N
N
(S)-5

4.3 (*S*)**-12**

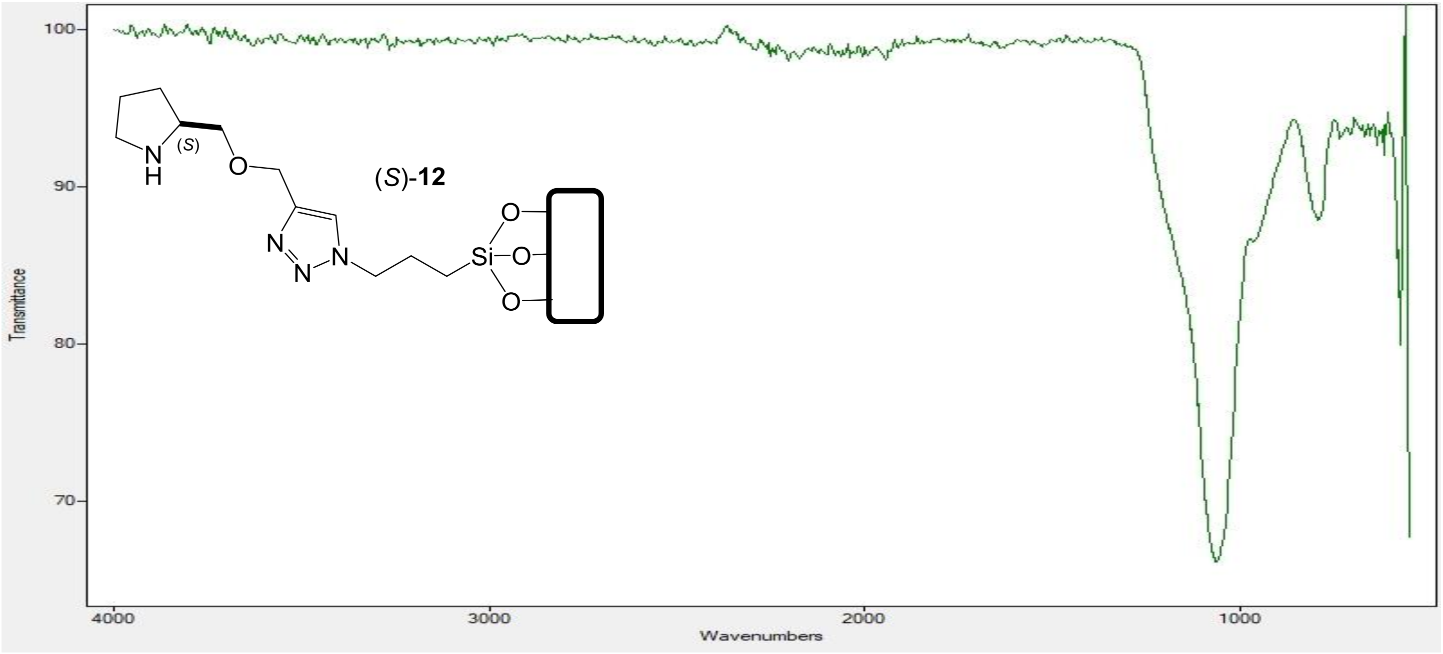

100
90
80
70
Transmittance
4000
3000
2000
1000
Wavenumbers
N
H
(S)
O
(S)-12
N
N
N
Si
O
O
O

4.4 (2*S*,4*R*,1`*R*)- **18**

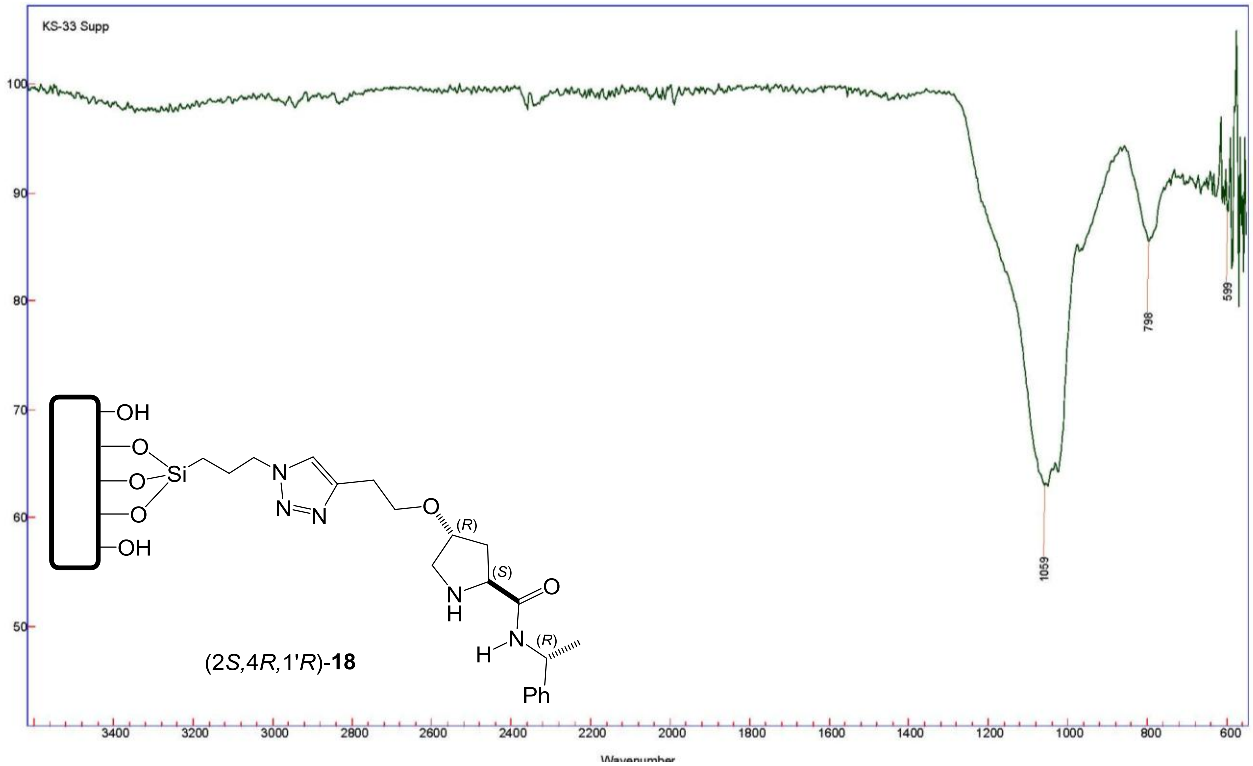

4.5 (2*S*,4*R*,1`*S*)- **18**

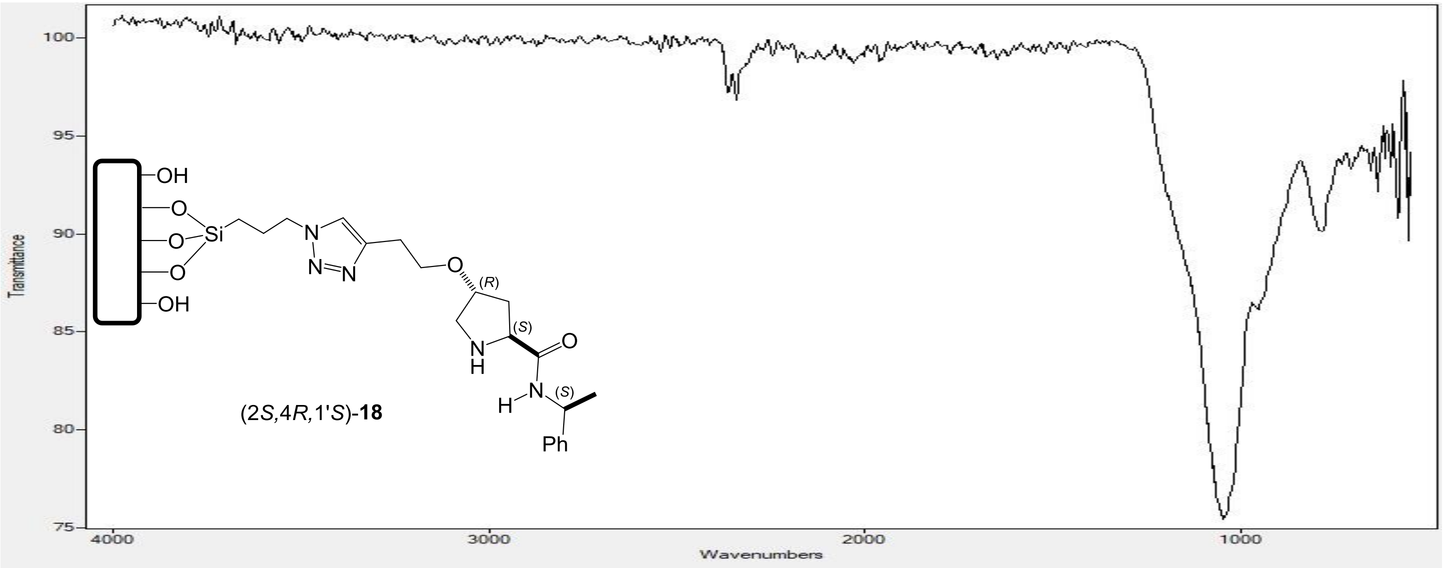

4.6 (*S*)**-5 recycled.**

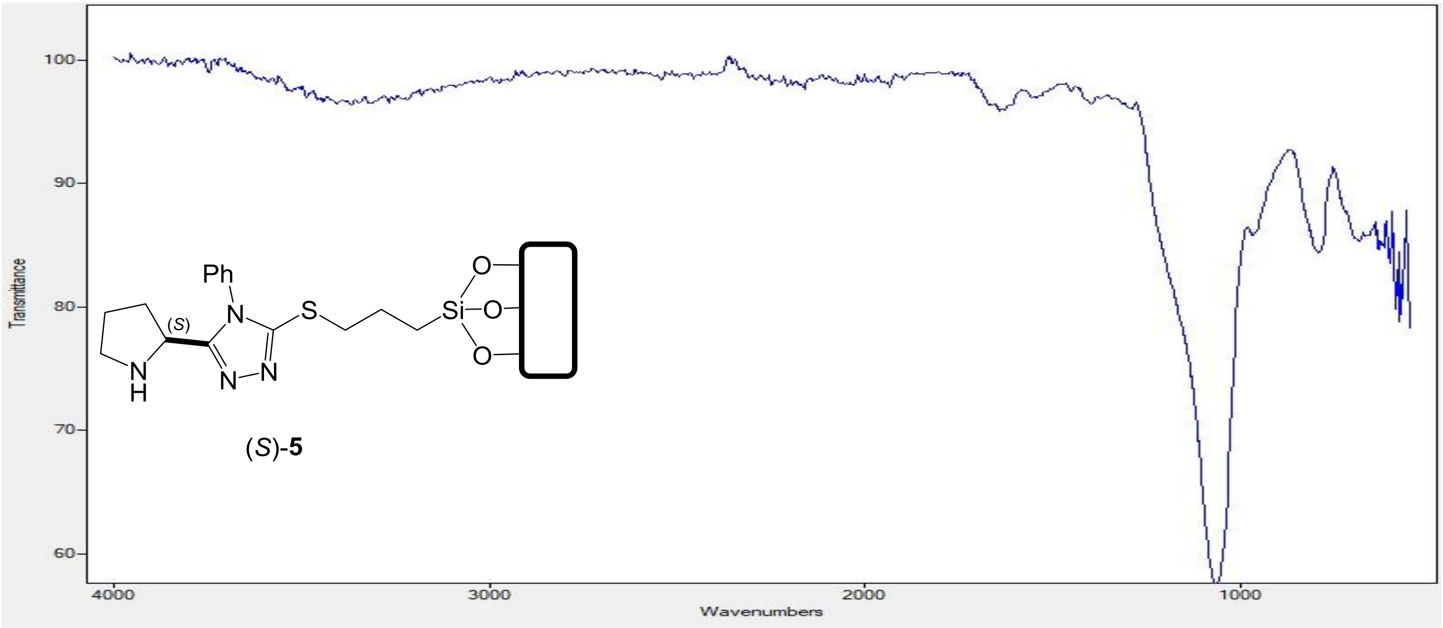

Supplement: Supplementary file 1 [file molecules-25-04532-s001.pdf]
